# How to assess antioxidant activity? Advances, limitations, and applications of in vitro, in vivo, and ex vivo approaches

**DOI:** 10.1186/s43014-025-00326-z

**Published:** 2025-10-29

**Authors:** Fereidoon Shahidi, Amal Samarasinghe

**Affiliations:** Department of Biochemistry, Memorial University of Newfoundland, St. Johns, NL A1C 5S7 Canada

**Keywords:** Oxidative stress, Clinical validation, Omics integration, High-throughput screening, Nanotechnology

## Abstract

Antioxidants are crucial in combating oxidative stress-a key factor in chronic diseases like cancer and neurodegeneration-while also playing vital roles in food preservation, functional food, and nutraceutical development. Their efficacy is assessed through in vitro (e.g., DPPH, FRAP), in situ, ex vivo, and in vivo models, as well as animal and human clinical trials, each offering unique advantages but facing challenges in standardization and physiological relevance. Advances in microfluidics, AI, and nanotechnology are enabling high-throughput screening, while omics integration provides deeper mechanistic insights. This review explores current methodologies, applications in food and pharmaceuticals, and emerging trends, emphasizing the need for rigorous clinical validation and personalized approaches to bridge the gap between laboratory research and real-world antioxidant therapies.

**Graphical Abstract:**

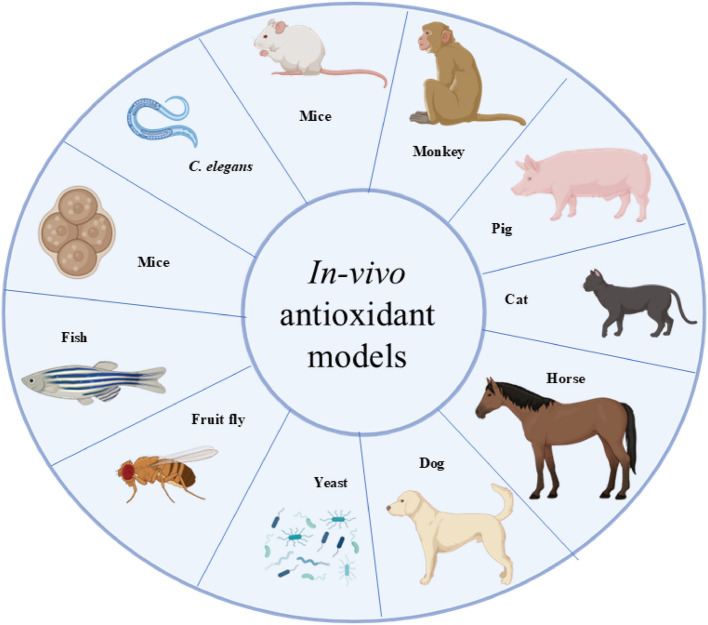

## Introduction

Antioxidants are widely recognized for their ability to prolong the shelf life of food and drugs by neutralizing harmful free radicals. While their effectiveness in stabilizing food and pharmaceutical products is well-established, their impact on human health remains less consistent (Hossain et al., 2022; Janssens et al., 2018). Antioxidants play a crucial role in maintaining health and preventing diseases by combating oxidative stress, which arises from an imbalance between free radical generation and antioxidant levels—an imbalance that creates a favorable environment for prooxidants (Li et al., 2025). The identification of antioxidants involves assessing the free radical-to-antioxidant ratio, as an imbalance indicates oxidative stress (Siddeeg et al., 2021). Oxidation is a critical process for energy production in living organisms (Xie et al., 2016a, b), and reactive oxygen species (ROS) and reactive nitrogen species (RNS) are by-products of normal respiration, metabolism, autoxidation of xenobiotics, and stress (Anraku et al., 2018; Hossain et al., 2022; Sridhar & Charles, 2019). Oxidative stress occurs when there is a disruption between ROS/RNS and the body’s antioxidant defense mechanisms (López-Alarcón & Denicola, 2013), leading to the production of harmful oxidation products such as hydroperoxides and DNA fragments, which cause cellular damage and inflammation (Pinchuk et al., 2012). This oxidative stress is a major contributor to tissue damage and is associated with the pathogenesis of chronic diseases, including cancer, cardiovascular diseases, diabetes mellitus, liver disorders, atherosclerosis, and neurodegenerative diseases (Pinchuk et al., 2012; Siddeeg et al., 2021; Xie et al., 2016a, b).

Peroxyl radicals (ROO•), harmful byproducts of lipid oxidation, negatively impact both thehealth and food quality. Assays measuring the ability of antioxidants to scavenge ROO• via hydrogen atom transfer (HAT) reactions are commonly used to evaluate antioxidant efficacy (Tomaz et al., 2021). Additionally, peroxynitrite, a potent oxidant, plays a significant role in various pathological conditions, including neurodegenerative diseases and inflammatory processes, making its measurement and inhibition critical for understanding biological effects and developing therapeutic interventions (Janssens et al., 2018; Prolo et al., 2024). Research has identified several natural compounds with significant peroxynitrite scavenging activity. For instance, xanthones from *Garcinia mangostana*, such as α-mangostin and γ-mangostin, effectively inhibit the peroxynitrite-induced oxidative damage, highlighting their therapeutic potential (Jung et al., 2006). Similarly, polyphenolic compounds like catechin and quercetin exhibit strong antioxidative properties, including the ability to neutralize peroxynitrite, underscoring the role of dietary antioxidants in mitigating oxidative stress (Jin et al., 2013; Shahidi & Ambigaipalan, 2015). Peroxynitrite's impact on cellular functions has been extensively studied; for example, exposure to peroxynitrite impairs chemotaxis in immune cells, indicating its detrimental effects on immune responses (Janssens & Stoks, 2019). In the context of neuroprotection, compounds that inhibit peroxynitrite-induced neurotoxicity have been identified, emphasizing the importance of developing effective inhibitors to protect neuronal cells from oxidative damage (Wąsik & Antkiewicz-Michaluk, 2017). Overall, peroxynitrite inhibition assays are valuable tools for screening compounds with potential therapeutic applications, particularly in addressing oxidative stress-related diseases. These assays provide critical insights into the mechanisms by which antioxidants neutralize peroxynitrite, contributing to the development of novel strategies for combating oxidative damage in biological systems (Liu et al., 2023). One common approach to assess peroxynitrite inhibition involves spectrophotometric methods, where the scavenging ability of compounds is measured through changes in absorbance. The investigation of natural antioxidants is essential, as synthetic antioxidants, while effective, can cause side effects such as liver damage and carcinogenesis at high levels and under certain conditions (Grzelakowska et al., 2024). Therefore, there is growing interest in exploring novel natural antioxidants that are both effective and safer alternatives to synthetic ones. Comparing the effectiveness of synthetic antioxidants used in the food industry with their residual effects in food and drugs highlights the importance of understanding their bioavailability and accessibility in living organisms. This knowledge helps identify and validate the efficacy of natural antioxidants in combating oxidative stress and related diseases (Xie et al., 2016a, b).

The body employs both enzymatic and non-enzymatic antioxidants, including vitamins C and E, glutathione (GSH), and various phytochemicals, to neutralize ROS and mitigate their harmful effects (Irato & Santovito, 2021). A diet high in antioxidants is inversely associated with the risk of oxidative-stress-related diseases (Vilela et al., 2015), and such diets enhance immune function, particularly in aging populations. For instance, antioxidant-rich diets are linked to a reduced incidence of cancer, likely due to improved immune responses (Kalogerakou & Antoniadou, 2024). Specific antioxidants, such as* N*-acetylcysteine (NAC), have demonstrated the ability to enhance immune cell function in aged mice, suggesting that dietary supplementation with antioxidants can help maintain immune competence during aging (Aldini et al., 2018), which is especially significant given the increased vulnerability of older individuals to oxidative stress and related diseases (Ferreira dos Santos et al., 2021). The role of antioxidants extends beyond scavenging free radicals; they also modulate immune responses. For example, flavonoids, which are abundant in fruits and vegetables, exhibit anti-inflammatory properties and enhance immune regulation by preventing DNA damage and promoting cellular repair mechanisms (Mukherjee et al., 2025). Carotenoids, which must be obtained through the diet, stimulate T-cell and antibody responses, emphasizing their importance in immune health (Berhane et al., 2023). Additionally, antioxidants such as astaxanthin, found in certain algae and other food sources, have been linked to improved immune regulation and anti-inflammatory effects, underscoring their potential in nutraceutical applications (Zhao et al., 2020). The synergistic effects of various antioxidants in a balanced diet provide comprehensive protection against oxidative stress and its associated health risks (Neacșu et al., 2025). The importance of antioxidants in health promotion and disease risk reduction cannot be overstated. As research continues to uncover the complexities of antioxidant interactions and their biological implications, it becomes increasingly evident that a diet rich in these compounds is essential for optimal health and longevity (Zehiroglu & Ozturk Sarikaya, 2019).

Currently, laboratory assessments focus on measuring the total in vivo, in vitro, and ex vivo antioxidant potential and correlating it with expected effects in food, nutraceutical, and pharmaceutical applications (Shahidi & Zhong, 2015). Additionally, in-situ antioxidant capacity detection techniques, particularly those based on electrochemical antioxidant testing methods, are also employed-especially in food and nutraceutical industries-for their rapid, sensitive, and reliable assessment of antioxidant activity. Recently, established antioxidant assays have been used to evaluate total antioxidant capacity (TAC), free radical scavenging activity, reducing power, lipid peroxidation inhibition potential, metal chelation ability, enzyme-based antioxidant activity, and protection of biomolecules in various systems (Siddeeg et al., 2021). Understanding the significance of antioxidant assays is essential for evaluating the mechanisms, effectiveness, and applications of antioxidants. These assays help identify new antioxidants from both synthetic and natural sources, aid in optimizing extraction and processing methods to enhance bioavailability, and support the development of food, pharmaceutical, and cosmetic products. Additionally, they contribute to sustainable sourcing and environmental applications, influence public health regulations, and serve as valuable educational tools (Ahmed, 2025).

The effectiveness of a specific antioxidant source or compound depends on multiple factors, including the food or system environment. In cosmetics, the growing demand for antioxidants is driven by their anti-aging, photoprotective, antimicrobial, and anti-inflammatory properties, as well as their role in preventing oxidative degradation of cosmetic components, such as lipids. However, incorporating antioxidants into cosmetic formulations presents challenges due to their instability and susceptibility to hydrolysis and photodegradation. Therefore, careful consideration of their hydrophilic or lipophilic nature, stability, and interactions with other ingredients is essential (Budzianowska et al., 2025; Silva et al., 2019). Moreover, there is a pressing need for rapid, low-cost analytical methods to measure TAC, as traditional methods often require specialized laboratories and trained personnel. In response, portable, user-friendly, and cost-effective techniques are gaining popularity for on-site evaluation of antioxidant activity in applications ranging from food to cosmetics and specialty products (Silva et al., 2019). Evaluating antioxidants is critical for their application in the nutraceutical, pharmaceutical, and cosmeceutical industries. Antioxidant activity is typically assessed through in vitro*, *in vivo, and ex vivo methods, each offering unique benefits and limitations. In vitro methods are especially favored for their simplicity and cost-effectiveness (Sadeer et al., 2020); however, no single assay can fully capture the complexity of antioxidant mechanisms, as different assays may highlight different aspects of antioxidant behavior (Gulcin, 2020).

In vivo methods assess antioxidant activity within living organisms, offering a holistic perspective on how antioxidants function in biological systems. Traditional animal models such as mice (Swiss, Kunming) and rats (Wistar, Sprague Dawley) are frequently used to evaluate the antioxidant effects of phenolic compounds, often by measuring biomarkers such as (Superoxide dismutase) SOD, glutathione peroxidase (GPx), and oxidative DNA damage markers like 8-OHdG (Lukitaningsih et al., 2019; Veskoukis et al., 2021; Martins et al., 2016). Innovative approaches, such as using developing chicken eggs, have shown promise in mimicking mammalian responses and enabling the study of oxidative stress biomarkers (Alam et al., 2013). Other model organisms, including *Caenorhabditis elegans*, zebrafish (*Danio rerio*), turquoise killifish (*Nothobranchius furzeri*), and fruit fly (*Drosophila melanogaster*), provide powerful platforms for studying oxidative stress, aging, and antioxidant responses due to their genetic tractability and conserved biological pathways (Lin et al., 2023). Ex vivo methods bridge in vitro and in vivo studies by assessing antioxidant activity in tissues or fluids extracted from organisms. For example, saliva and gingival crevicular fluid have been analyzed to evaluate local antioxidant capacity in conditions like periodontal disease (Toczewska et al., 2020), while lipid peroxidation levels (e.g., malondialdehyde, MDA) in blood samples from oxidative-stressed rats offer further insights into systemic antioxidant effects. In vitro methods, widely favored for their simplicity and cost-effectiveness, involve cultured cell lines such as HepG2 to assess antioxidant potential via biochemical assays targeting enzymatic activity or free radical scavenging (Gulcin, 2020; Sadeer et al., 2020). While no single method can fully capture the complexity of antioxidant mechanisms, each-whether in vitro, ex vivo, or in vivo-offers distinct advantages in vitro provides mechanistic insights, ex vivo reflects near-physiological conditions, and in vivo yields real-world biological responses (Matute et al., 2021; Thangaraj, 2016; Weydert & Cullen, 2010). The growing interest in antioxidants has spurred the development of various methods to assess antioxidant activity in individual compounds and complex matrices such as foods, beverages, and body fluids. Though the clinical relevance of TAC in biological fluids remains debated, TAC assays are widely accepted in food science and cosmetic applications (Kut et al., 2022a, b). Additionally, there is increasing demand for rapid, low-cost, and portable antioxidant assays for field applications across the food, pharmaceutical, and cosmeceutical industries (Silva et al., 2019). Ultimately, selecting appropriate antioxidant evaluation methods requires consideration of the research context and the physicochemical nature of the compounds being studied, reinforcing the importance of a multifaceted approach in antioxidant research (Apak et al., 2016; Shahidi & Zhong, 2015).

Given the growing demand for potent and safe antioxidants, both academic and industrial researchers invest considerable resources into identifying novel compounds with superior antioxidative potency compared to conventional ones like vitamins C and E. These efforts aim to develop antioxidants with improved radical-scavenging capacity, better organ distribution, and favorable pharmacokinetics (Janssens et al., 2018). To support this, the availability of assays that are simple, reproducible, cost-effective, high-throughput, and biologically relevant is essential. Numerous methods have been developed to evaluate antioxidant activity, ranging from basic to highly refined techniques, some of which were prioritized for standardization during “The First International Congress on Antioxidants Methods” (Papaefthimiou et al., 2024). In practice, widely used commercial kits offer convenience and reduce irreproducibility, but the absence of a universally accepted gold standard makes established techniques de facto benchmarks, necessitating critical awareness of their limitations for accurate interpretation (Khan et al., 2015). This review justifies a comprehensive evaluation of antioxidant assays across in vitro, in vivo, and ex vivo systems, aiming to clarify their mechanisms, applications, and practical strengths and weaknesses. By guiding the selection of context-appropriate methods, and by addressing emerging trends and innovations, the review supports the design of biologically meaningful, efficient studies-particularly in fields like food science, pharmaceuticals, and nutraceuticals-where antioxidant functionality is key to product performance and health outcomes.

## Comparison of extraction, isolation and purification methods for different antioxidants

Extraction is a critical step in isolating and identifying antioxidants from plant materials, as the method employed significantly impacts yield, solubility, and the efficiency of retrieving bioactive compounds (Lezoul et al., 2020). The efficiency of extraction is influenced by several factors, including temperature, solvent type, extraction time, and sample pre-conditioning. For instance, higher temperatures can enhance the solubility of compounds but may also degrade thermolabile antioxidants. Similarly, the choice of solvent plays a crucial role in determining the polarity and solubility of target compounds, with water and ethanol being commonly used due to their safety and sustainability (Jaimez-Ordaz et al., 2021). Recent studies have emphasized the importance of sample pre-treatment, such as drying, grinding, and particle size in improving extraction yields (Santos et al., 2022). Proper extraction methods are essential for obtaining bioactive compounds with antioxidant properties while preserving their beneficial characteristics. The choice of method depends on the chemical structure of the compounds and the cell matrix (Jaimez-Ordaz et al., 2021). Solvent polarity influences the solubility of bioactives, while extraction techniques, time, and temperature impact the overall efficiency of the process. Extraction methods can be broadly categorized into traditional and advanced techniques, each with unique advantages and limitations (Jaimez-Ordaz et al., 2021; Quitério et al., 2022). Recent advancements in extraction technologies have significantly improved the efficiency, selectivity, and sustainability of these processes.

Conventional extraction methods, based on the principle of'like dissolves like,'are simple in principle and easy to operate but often require large volumes of solvent, extended extraction times, and high temperatures, which can degrade phenolic compounds (Shi et al., 2022). Traditional extraction methods, such as maceration, Soxhlet extraction, solid–liquid extraction, shaking, and heated reflux, have been widely used for decades. Maceration involves soaking plant material in a solvent, allowing bioactive compounds to diffuse into the solvent over time. This method is simple and cost-effective, making it ideal for small and medium enterprises (Lezoul et al., 2020). However, it is a cold extraction method that yields lower amounts of secondary metabolites compared to hot extraction techniques due to slower diffusion rates (Saptarini & Wardati, 2020). Decoction, which uses high temperatures, can either degrade or preserve bioactive compounds by deactivating enzymes (Lezoul et al., 2020). Soxhlet extraction employs continuous solvent cycling to enhance extraction efficiency but may yield lower amounts of certain compounds, such as anthocyanins, due to thermal degradation (Saptarini & Wardati, 2020). Solid–liquid extraction relies on solvents to dissolve target compounds; while shaking and heated reflux methods improve efficiency through mechanical agitation and thermal energy (Jaimez-Ordaz et al., 2021). Hot water extraction involves boiling the materials in water to extract polymers with antioxidant compounds, which is low-cost, simple, and safe but yields low amounts (10–40%) and may damage antioxidant polymers due to high temperatures (Mohanta et al., 2023). Acid extraction uses acidic solutions to break down cell walls and release polysaccharides, achieving high extraction rates but potentially damaging antioxidant polymers and breaking glycosidic bonds. For example, polysaccharides from *Cordia dichotoma* fruits were extracted using 1% HCl, yielding 86.24% polysaccharide content (Mohanta et al., 2023). Alkali extraction, which uses alkaline solutions to dissolve cell wall materials, offers high yields and shorter extraction times but may break glycosidic bonds and affect polysaccharide structure and color, making it suitable for acidic polysaccharides and those containing uronic residues (Mohanta et al., 2023). Reflux extraction, which involves continuous evaporation, condensation, and recycling of the solvent, offers shorter operation times and lower solvent volumes but may degrade phenolic compounds due to heating during evaporation (Shi et al., 2022). Similarly, Soxhlet extraction, a modified reflux method, is suitable for bulk extraction but may also degrade phenolic compounds due to heat exposure (Shi et al., 2022). For instance, polysaccharides from *Lycium barbarum* fruits were extracted with a 5.87% yield, showing antioxidant, immunomodulatory, and neuroprotective activities (Mohanta et al., 2023). Reflux extraction, which uses heat to increase the diffusion of secondary metabolites, yields higher amounts than maceration but is less efficient than percolation (Saptarini & Wardati, 2020). Percolation, a cold extraction method involving the continuous flow of solvent through plant material, produces the highest yield among traditional methods, as it allows complete extraction of secondary metabolites at ambient temperature (Saptarini & Wardati, 2020). Despite their widespread use, traditional methods often require large amounts of solvents, extended extraction times, and high temperatures, which can degrade thermolabile compounds and reduce antioxidant activity (Chemat et al., 2020).

In contrast, advanced extraction methods, such as microwave-assisted extraction (MAE), ultrasound-assisted extraction (UAE), and supercritical fluid extraction (SFE), have gained popularity due to their efficiency, reduced solvent consumption, and shorter extraction times. MAE utilizes microwave energy to heat the solvent and plant matrix, facilitating the release of bioactive compounds. This method enhances solvent penetration and reduces extraction time, making it particularly effective for thermostable compounds. Studies have shown that MAE yields higher antioxidant activity compared to traditional methods (Lezoul et al., 2020; Mirzadeh et al., 2020). For example, polysaccharides from *Camptotheca acuminata* were extracted with an 8.61% yield using MAE (Mohanta et al., 2023). UAE employs ultrasonic waves to create cavitation bubbles, which disrupt cell walls and enhance the release of intracellular compounds. This method is effective at lower temperatures, making it suitable for heat-sensitive antioxidants. One of the prominent techniques discussed is UAE, which has been shown to significantly improve the extraction efficiency of antioxidants. For instance, Elnour et al. (2018) noted that UAE, along with other modern techniques such as microwave-assisted extraction, has led to higher yields of antioxidant compounds compared to traditional methods. Similarly, Younas et al. (2021) demonstrated that UAE combined with response surface methodology optimized the extraction conditions for antioxidants from *Artemisia annua*, resulting in enhanced recovery of bioactive components. This aligns with findings by Ghazzawi et al. (2021), who emphasized that the choice of extraction solvent and technique directly influences the antioxidant capacity of the extracts. UAE offers advantages such as short extraction time, reduced solvent consumption, and high extraction yield (Chemat et al., 2020; Lezoul et al., 2020; Yang et al., 2021a, b). For instance, polysaccharides from mulberry fruits were extracted with a 3.13% yield using UAE (Mohanta et al., 2023). Enzymolysis, which uses enzymes to hydrolyze cell walls and release polysaccharides, offers high efficiency and gentle conditions but is limited by the high cost of enzymes. Ultrasound-assisted enzyme extraction (UAEE) was used to extract polysaccharides from *Cucurbita moschata* fruits with a 4.33% yield (Mohanta et al., 2023). UAEE which combines ultrasound and enzymatic treatment, was identified as the most efficient method for extracting polyphenols from *Ascophyllum nodosum* using alcalase enzyme for 3 h, UAEE achieved the highest extraction yield of 1.913 g phloroglucinol equivalents (PGE) per 100 g DW of macroalgae. This is energy-efficient, reduces extraction time, and minimizes solvent use compared to conventional techniques (Meng et al., 2023).

Homogenization-assisted extraction (HAE) relies on strong shear forces to increase molecular motion, facilitating material breakdown and dispersion. While HAE enhances extraction efficiency, prolonged homogenization can cause oxidative degradation due to polyphenol oxidase (PPO) activity (Yang et al., 2021a, b). Combination methods, such as homogenization-ultrasound-assisted extraction (HUAE) and ultrasound-homogenization-assisted extraction (UHAE), synergistically improve extraction efficiency by maximizing polyphenol release while minimizing oxidation (Yang et al., 2021a, b). SFE uses supercritical fluids, such as carbon dioxide (CO₂), to selectively extract target compounds. The tunable properties of supercritical fluids, such as density and solubility, allow for precise control over the extraction process, resulting in high-purity extracts (Santos et al., 2022). Enzymatic-assisted extraction (EAE) is an emerging technique that uses enzymes to break down cell walls and release bioactive compounds. This method is particularly effective for extracting polysaccharides and other high-molecular-weight compounds. EAE operates at low temperatures, preserving bioactivity, and has been shown to produce polysaccharides with better antioxidant effects compared to MAE (Mirzadeh et al., 2020). Additionally, EAE is considered environmentally friendly, as it reduces the need for organic solvents and operates under mild conditions (Yang et al., 2021a, b).

Experimental designs, such as Box-Behnken and central composite designs, have been widely used to optimize extraction conditions. These designs allow researchers to evaluate the effects of multiple factors, such as temperature, time, solvent concentration, and sample amount, on extraction efficiency. For example, a study on *Decatropis bicolor* optimized extraction conditions using these designs and found that key factors influencing extraction efficiency included temperature, time, sample amount, and extraction method. The models developed in the study had correlation coefficients (R^2^) of ≥ 0.85, indicating high reliability (Jaimez-Ordaz et al., 2021). Advanced methods such as UAE and MAE achieve comparable or higher yields at lower temperatures and shorter times. UAE, based on the principle of cavitation bubble implosion, releases phenolic compounds through surface peeling, erosion, and particle breakdown. It offers short operation times, low costs, and small solvent volumes but may generate heat that degrades phenolic compounds (Shi et al., 2022). MAE, which uses microwave energy to heat solvents, achieves optimal results at 30–40% power, 1–2 min, and 2% sample concentration, yielding high antioxidant activity values (> 870 mg ET/100 g DPPH and > 260 mg EFe^2^⁺/100 g FRAP). However, it has high energy costs and may degrade phenolic compounds if not carefully controlled (Shi et al., 2022). The French press method has emerged as one of the most efficient techniques, requiring the lowest sample amount (0.34%) and shortest extraction time (5 min). This method achieved the highest antioxidant activity (1.75 g ET/100 g DPPH and 1.25 g EFe^2^⁺/100 g FRAP) and total phenolic content (7.50 g EGA/100 g) compared to other methods. Its efficiency is attributed to high pressure and mechanical disruption of cell walls, which enhances the release of bioactive compounds (Jaimez-Ordaz et al., 2021). Pressurized liquid extraction (PLE), which extracts samples under high temperature and pressure for 5–15 min, offers shorter operation times, small solvent volumes, and good repeatability but may degrade phenolic compounds due to heating (Shi et al., 2022). SFE, which uses supercritical CO₂, operates at low temperatures with high selectivity and inertness but requires careful co-solvent selection and has high costs (Shi et al., 2022). Subcritical water extraction uses water at temperatures above the boiling point but below the critical point to extract polysaccharides, offering energy efficiency and high extraction rates (Mohanta et al., 2023). Pulsed electric field extraction (PEF), which uses high-voltage pulses to destroy membrane structures, offers short operation times and low temperatures but faces challenges such as reversible membrane changes and air bubbles that reduce efficiency (Shi et al., 2022). EAE, which uses enzymes like cellulase, Viscozyme and Alcalase to decompose cell membranes, operate at low temperatures but requires long operation times (1–4 h) and careful control to avoid impurities and enzymatic hydrolysis aids in breaking down cell walls, enhancing the release of bioactives (Shi et al., 2022).

UAEE, combining ultrasound and enzymatic treatment, was identified as the most efficient method for extracting polyphenols from *Ascophyllum nodosum* using Alcalase for 3 h, UAEE achieved the highest extraction yield of 1.913 g phloroglucinol equivalents (PGE) per 100 g dry weight (DW) of macroalgae. This method is energy-efficient, reduces extraction time, and minimizes solvent use compared to conventional techniques (Meng et al., 2023).

Water-based extraction methods, such as the French press, are environmentally friendly and mimic traditional infusion practices. These methods avoid the use of organic solvents, making them safer and more sustainable. Additionally, water-based methods are particularly effective for extracting polar compounds, such as phenolic acids and flavonoids, which are often associated with antioxidant activity (Santos et al., 2022). Ultrafiltration, which uses semipermeable membranes to separate polysaccharides, offers high capacity and low cost but is sensitive to salts and organic contamination (Mohanta et al., 2023).

Overall, the choice of extraction method depends on the specific requirements of the study, including the target compounds, desired yield, and the need to preserve bioactivity while minimizing environmental impact. Extracts are intricate mixtures composed of various natural compounds with differing polarities. Consequently, additional separation and purification steps are necessary to obtain pure bioactive compounds with antioxidant properties (Popova & Bankova, 2023). A range of techniques has been identified for isolating or purifying bioactive compounds with antioxidant activity. Traditional high-performance liquid chromatography with ultraviolet detection (HPLC–UV) shows limited sensitivity for oxidizable phenolics, and isolated component analyses fail to capture synergistic effects (Abedelmaksoud et al., 2025). In contrast, high performance liquid chromatography (HPLC) coupled with electrochemical detection (ECD) provides superior selectivity for redox-active phenolic moieties through their oxidation-prone phenolic hydroxyl groups. When combined with liquid chromatography-tandem mass spectrometry (LC–MS/MS) for structural elucidation and multivariate statistical analysis, this approach enables comprehensive quality assessment and identification of bioactive antioxidant markers in complex matrices like food and herbal medicines (Guiard & Gotti, 2024). Other key separation and characterization techniques include membrane filtration, solid-phase extraction (SPE) followed by gas or liquid chromatography (GC/LC), capillary electrophoresis (CE), countercurrent chromatography (CCC), and centrifugal partition chromatography (CPC), offering a versatile toolkit for antioxidant analysis (Susanti et al., 2024; Zhang et al., 2018). These techniques enable the isolation of antioxidant compounds with high precision and efficiency, contributing to the development of natural antioxidant-based products (Susanti et al., 2024). Table 1 summarizes the different methods employed for the isolation and purification of antioxidants, including the sample sources, target compounds, techniques used, stationary phases/columns, elution systems, yields, purity levels, and key findings.

**Table 1:** Summary of isolation and purification techniques for antioxidants

Sample Source	Target Compound(s)	Technique Used	Stationary Phase/Column	Elution System	Yield (%)	Purity (%)	Key Findings	Reference
Medium Pressure Liquid Chromatography (MPLC)								
*Saxifraga atrata* (dry plant)	Bergenin (phenolic acid glycoside)	MPLC (two-step)	1. Polyamide column (15 × 460 mm) 2. MCI GEL® CHP20P (15 × 460 mm)	1. Water/ACN gradient 2. Isocratic (5% ACN, 60 min)	0.67% (714.2 mg from 180 g)	> 99%	High-purity bergenin isolated using cost-effective MPLC with polyamide and MCI GEL® CHP20P	Dang et al., b
*Saxifraga atrata*	Ethyl gallate, 11-O-galloylbergenin, rutin, isoquercitrin (DPPH inhibitors)	MPLC + HPLC (RP-C18/HILIC)	MCI GEL® CHP20P (49 × 460 mm)	Methanol/water	11.9% recovery	> 95%	Combined MPLC-HPLC with online DPPH detection efficiently isolated antioxidant phenolics	Dawa et al., 2021
*Saxifraga sinomontana*	3-methoxy-4-hydroxyphenol-(60-O-galloyl)−1-O-β-D-glucopyranoside (1) and others (2–4)	MPLC	Not specified	Not specified	0.6	> 95%	Isolated phenylpropanoid glycosides (1–2) and phenolic acids (3–4) with potent antioxidant activity	Dang et al., 2021a
High Performance Liquid Chromatography								
Banana leaves (*Musa balbisiana*)	Rutin	Sephadex + Semi-prep RP-HPLC	Sephadex column → RP-HPLC column	Not specified (RP-HPLC conditions)	3.24	98.4%	Semi-prep RP-HPLC increased rutin purity from 74–84% to 98.4%	Yingyuen et al., 2020
*Chaerophyllum bulbosum extract*	Quercetin-3-O-β-D-glucopyranoside	Recycle HPLC	GS-320 column	100% Methanol	0.011	High purity	Isolated flavonoids showed superior DPPH•/ABTS• + scavenging vs. BHA/α-tocopherol. Recycle HPLC improved separation efficiency	Molo et al., 2021
*Hippocrepis emerus flowers*	Quercetin-3-O-α-L-rhamnopyranosyl-(1 → 6)-β-D-glucopyranoside	Prep-HPLC	Not specified	15–25% acetonitrile gradient (45 min)	0.517	High purity	Compound exhibited 1.6 × higher DPPH activity than quercetin and 2 × higher than ascorbic acid; strong OH radical/CUPRAC activity (IC50s provided)	Schmitt et al., 2020
Counter-current chromatography (CCC) systems								
*Weinmannia trichosperma *bark	Isoastilbin, neoisoastilbin, neoastilbin	CPC(Centrifugal partition chromatography)	Liquid stationary phase	HEMWAT (1:9:1:9, hexane/ethyl acetate/methanol/water)	Not Mentioned (NM)	NM	Isolated compounds showed potent antioxidant activity (DPPH, ABTS, FRAP)	Barrientos et al., 2020; Zhao et al., 2020
*Malus hupehensis*	Avicularin, phloridzin, sieboldin	DES-HSCCC (Deep Eutectic Solvent High-Speed Counter-Current Chromatography)	Liquid stationary phase	DES: choline chloride/glucose/water/ethyl acetate (1:1:2)	NM	> 92%	DES-HSCCC is eco-friendly; avicularin showed 87.54% OH radical scavenging at 100 mg/L	Cai et al., 2021; Lee et al., 2019
*Castanopsis chinensis*	Chinensin D, chinensin E	HSCCC + column chromatography	Liquid stationary phase	Hexane/ethyl acetate/methanol/water (1:6:3:4)	NM	93.0%, 95.7%	HSCCC improved purity of antioxidant phenolic glycosides	Wang et al., 2023
Sweet orange peel	Sinensetin, nobiletin, tangeretin, etc	HPCCC	Liquid stationary phase	Hexane/ethyl acetate/methanol/water (normal & reverse phase ratios)	1.08–14.25%	96.6–100%	HPCCC achieved high purity polymethoxyflavones rapidly	Xu et al., 2019
*Achyrocline satureioides*	Quercetin, luteolin, 3-O-methylquercetin	HPCCC + HPLC	Liquid stationary phase	Hexane/ethyl acetate/methanol/water (0.8:1.0:0.8:1.0)	60–90.2%	97–97.5%	High recovery of antioxidant flavonoids	Bianchi et al., 2019
Hydrophilic Interaction Liquid Chromatography								
*Saxifraga tangutica*	Hyperoside, luteolin-glucoside, trifolin	2D HILIC/RPLC(Two-Dimensional Hydrophilic Interaction Liquid Chromatography/Reversed-Phase Liquid Chromatography)	Not specified	Not specified	NM	> 95%	Successful separation of flavonoid glycosides	Dang et al., 2018
*Salvia prattii*	Caffeic acid, ethyl rosmarinate, methyl rosmarinate, rosmarinic acid	2D HILIC/RPLC	Not specified	Not specified	1.09–8.90	> 98%	Efficient isolation of phenolic acids	Dang et al., 2016
*Arenaria kansuensis*	Tricin, homoeriodictyol, luteolin	2D RP/HILIC (HPLC-DPPH-guided)	Not specified	Not specified	NM	> 98%	Isolation of flavonoid compounds	Cui et al., 2017
Column Chromatography								
*Endopleura uchi*	Bergenin	Silica gel chromatography	Silica gel LC60A (70–200 μm)	Chloroform/ethanol (7:3) isocratic	5.4% (leaves), 5.73% (twigs), 6.09% (bark)	NM	Successful isolation from different plant parts	Muniza et al., 2020
*Alseodaphne semecarpifolia* Nees	Icariin, baicalein	Silica gel chromatography	Silica gel	Gradient: n-hexane → ethyl acetate → petroleum ether → chloroform	Icariin (1.34%), Baicalein (1.23%)	NM	Gradient elution improved separation efficiency	Chethankumara et al., 2021
*Boesenbergia rotunda*	2′,4′-dihydroxy-6-methoxychalcone, 5-hydroxy-7-methoxyflavanone, 5,7-dihydroxyflavanone	Silica gel chromatography	Silica gel	Hexane–ethyl acetate (6:4)	0.125%, 0.35%, 0.2%	NM	Chalcone and flavanones isolated with varying yields	Atun et al., 2018
*Perilla frutescens (L.)* Britt	Ferulic acid, luteolin, apigenin, caffeic acid, rosmarinic acid	Mixed-phase chromatography	Silica gel + Sephadex LH-20	Chloroform:methanol (varying ratios); 90% methanol (for Sephadex)	0.042%, 0.033%, 0.042%, 0.024%, 0.16%	NM	Combined silica gel and Sephadex improved separation. Rosmarinic acid had the highest yield	Lee & Cho, 2021
Pomelo peels	Naringin	Sephadex LH-20 chromatography	Sephadex LH-20	30% ethanol	NM	95.7 ± 0.23%	High-purity naringin isolated	Shangguan et al., 2023
Mulberry fruit* (Morus alba L.)*	(2R)-eriodictyol, quercetin	Sephadex LH-20 chromatography	Sephadex LH-20	Methanol/water (50:50 → 70:30)	0.006%, 0.0004%	NM	Very low yields but successful isolation	Xu et al., 2020
Supercritical Fluid Chromatography (SFC)								
Turmeric	Curcumin, Demethoxycurcumin, Bisdemethoxycurcumin	Preparative SFC	NM	CO₂ + organic modifier	76.6 (mean recovery)	97.9, 91.1, 94.8	Efficient single-step separation with high purity and recovery	Song et al., 2015
*Fructus Cnidii*	Osthole, Imperatorin	Semi-preparative SFC	NM	CO₂ + organic modifier	19.6, 24.4	98.9, 98.2	Effective separation of coumarins	Zhang et al., 2017a, b, c
Molecularly Imprinted Technology								
Broccoli	Sinapic acid	Bulk polymerization (MIP)	4-vinyl pyridine (functional monomer), EGDMA (crosslinker)	DMSO (porogen), methanol-acetic acid (9:1) for elution	NM	High selectivity	MIP showed 90% binding affinity for sinapic acid, with lower affinity for analogues (ferulic acid: 60%, caffeic acid: 11%). Scatchard plot indicated heterogeneous binding sites	Hosny et al., 2018
*Asafoetida*	Sesquiterpene coumarins	Dummy template MIP (DMIP)	Methacrylic acid (MMA) as functional monomer	NM	3 × peak area increase after DMISPE	Improved clean-up	DMIP (using 7-hydroxycoumarin as dummy template) enhanced selective extraction of sesquiterpene coumarins	Eidi et al., 2020
*Rosmarinus officinalis* L	Rosmarinic acid	Molecularly Imprinted Solid Phase Extraction (MISPE) using bulk polymerization	NM	NM	49.11 ± 4.58 mg/g	NM	Adsorption capacity: 15.49 mg/g	Saad et al., 2021
*Spina Gleditsiae*	Quercetin	Pickering emulsion polymerization	NM	NM	NM	NM	Adsorption capacity: 0.521 mg/g	Sun et al., 2019a, b
*Carthamus tinctorius* L., *Abelmoschus manihot*	Myricetin	Precipitation polymerization (MISPE)	NM	NM	79.82–84.32%	NM	Adsorption capacity: 11.80 mg/g	Wan et al., 2018
*Salvia officinalis *leaves	Rosmarinic acid	Ultrasound-Assisted Dispersive Solid Phase Extraction(UA-DSPE)	NM	NM	77.80%	NM	Ultrasonic-assisted DSPE improved efficiency	Alipour et al., 2021)
High-Performance Thin Layer Chromatography (HPTLC)								
Japanese knotweed rhizome bark extract	(+)-catechin, (-)-epicatechin, (-)-epicatechin-gallate, procyanidin B1-B3, proanthocyanidin B dimer gallate, emodin, emodin-8-O-glucoside, emodin-8-O-malonyl-glucoside	Offline multidimensional HPTLC	1 st dimension: Preparative TLC silica gel 2nd dimension: HPTLC cellulose & silica gel 3rd dimension: HPTLC silica gel	Not specified	Not specified	Not specified	- Successful isolation of flavan-3-ols, proanthocyanidins, and anthraquinones - Post-chromatographic derivatization with DMACA for identification - HPTLC-MS used for characterization - Advantages: Fast separation, in-situ derivatization, minimal sample prep	Golfakhrabadi et al., 2021; Jug et al., 2021

The extraction and purification processes are critical in determining the antioxidant capacity of isolated compounds. For instance, the purification of polyphenols from *Limnocharis flava* demonstrated that purified extracts exhibited stronger antioxidant activity compared to crude extracts, emphasizing the importance of effective isolation techniques (Sudirman et al., 2023). For the purification ethyl acetate was used for liquid–liquid partition, achieving the highest polyphenol content (73.85 g PGE/100 g extract). This fraction contained 74% polyphenols with a molecular weight (MW) greater than 10 kDa (Meng et al., 2023).

Microbial sources, such as endophytic fungi associated with plants like *Vitex payos*, have also been identified as producers of phenolic compounds with strong antioxidant properties, attributed to their redox properties and free radical scavenging abilities (Sibanda et al., 2018). Similarly, the fermentation of squid pen by *Paenibacillus sp.* yielded homogentisic acid, which displayed significant antioxidant and anti-inflammatory activities, highlighting the potential of microbial fermentation in enhancing antioxidant yield (Wang et al., 2016a, b). In the separation of polyphenols, molecular weight cut-off (MWCO) filtration was used for isolating high molecular weight polyphenols which are predominant in A. *nodosum* (Meng et al., 2023).

Antioxidant peptides from animal sources, such as tilapia frame protein, have shown promising results, particularly those containing cysteine, which exhibit strong antioxidant activity by directly interacting with free radicals (Fan et al., 2012). The generation of antioxidant peptides from meat proteins is an area of growing interest, as these compounds can be incorporated into functional foods for their health benefits (Zhu et al., 2023). Microalgae, rich in bioactive compounds, have also gained attention for their antioxidant properties, with recent studies focusing on optimizing extraction and purification methods to maximize efficacy (Pereira et al., 2023). The sustainable cultivation of microalgae further supports their potential for large-scale antioxidant production, benefiting both health and environmental sustainability. The HPLC–DAD assay is a critical tool for the purification and analysis of antioxidants and as an authentic example betanin, a natural pigment from beetroot, allowing for the separation and quantification of betanin and its isomer isobetanin based on their distinct retention times and absorption at 536 nm. This assay is significant for ensuring the purity and stability of betanin, which can be preserved for up to 275 days at − 30 °C, making it suitable for use as a natural food additive. For example, the assay demonstrated that purified betanin maintained its structural integrity and antioxidant properties, with a yield of 4% from fresh beetroot juice, highlighting its potential in functional foods and nutraceuticals (Vieira Teixeira da Silva et al., 2019).

Walnut protein has been explored as a sustainable source of antioxidant peptides, with ultrafiltration and Sephadex G-25 chromatography used for purification, followed by Nano LC–MS/MS for peptide identification (Zhong et al., 2024). Similarly, ultrafiltration membranes are commonly used to separate protein hydrolysates and polysaccharides based on molecular weight, though this method can be time-consuming and may reduce polysaccharide yield (Mohanta et al., 2023). Gel filtration chromatography, reverse-phase high-performance liquid chromatography (RP-HPLC), and macroporous resin are additional techniques employed for peptide purification (Mohanta et al., 2023). For instance, antioxidant peptides from chicken blood hemoglobin were purified using dextran gel chromatography after ultrafiltration, with the < 3 kDa fraction exhibiting the highest antioxidant activity (Wang et al., 2024).

Polysaccharides are often purified using column chromatography, including cellulose column chromatography, anion exchange chromatography, gel chromatography, and affinity chromatography (Mohanta et al., 2023). For example, crude polysaccharides from *Lonicera japonica* Thunb. were extracted using hot water, deproteinized via the Sevag method, and purified using DEAE-52 column chromatography and gel filtration chromatography (Liu et al., 2020). Similarly, yak casein hydrolysate (YCH) was fractionated using ultrafiltration, with the < 3 kDa fraction (YCH-4) showing the highest antioxidant activity due to its superior free radical scavenging and metal chelation properties (Liu et al., 2020). Additionally in the gel filtration chromatography Sephadex LH-20 was used to separate polyphenols based on polarity. Less polar polyphenols exhibited the highest antioxidant activities (Meng et al., 2023).

A combination of ultrafiltration, gel filtration, and RP-HPLC is often used for efficient purification. For example, water hyacinth leaf protein hydrolysate was purified using macroporous resin, gel filtration, and RP-HPLC to isolate antioxidant peptides like Phe-Phe-Glu and Leu-Phe (Xue et al., 2019). Similarly, polysaccharides from *Tuber sinense* were purified using DEAE-52 and Sephadex G-100 columns, with the final purified polysaccharide (PTS-A) analyzed for purity using HPLC (Zeng et al., 2020a, b). High-speed counter-current chromatography (HSCCC) guided by online DPPH-HPLC analysis has also been used to isolate antioxidant compounds from *U. laetevirens*, identifying isovitexin, isoorientin, and apigenin-6,8-di-C-β-D-glucopyranoside as key compounds (Li et al., 2023).

Enzymatic hydrolysis, using proteases like trypsin, pepsin, alkaline protease, and papain, is another effective method for generating antioxidant peptides. Alkaline protease has been shown to produce peptides with strong antioxidant activity (Xue et al., 2019). For example, yak casein hydrolysate was produced using alcalase and trypsin, with the < 3 kDa fraction (YCH-4) exhibiting the highest antioxidant activity (Liu et al., 2020). Pan et al. (2020) reviewed various purification techniques for peptidic antioxidants, including enzymatic hydrolysis, gastrointestinal digestion, and microbial fermentation, highlighting their potential in releasing bioactive peptides.

The advanced purification techniques, such as ultrafiltration, chromatography, and enzymatic hydrolysis, are essential for isolating and characterizing antioxidant compounds from diverse sources. These methods not only enhance the efficacy of antioxidant extraction but also support their application in functional foods and pharmaceuticals, contributing to health and sustainability initiatives.

## Chemical characterization of antioxidant compounds

In the chemical characterization of antioxidants, ultra-high performance liquid chromatography coupled with tandem mass spectrometry (UHPLC-MS/MS) analysis is a powerful tool for identifying and characterizing low-molecular-weight phenols and polyphenols in *Ascophyllum nodosum*, such as 4-hydroxybenzaldehyde and caffeic acid. Additionally, molecular weight and polarity separation techniques, including MWCO filtration and Sephadex LH-20 chromatography, reveal that polyphenols with molecular weights of 1–3 kDa and less polar fractions exhibit the highest antioxidant activities, as demonstrated by their superior ABTS, DPPH, and FRAP values (Meng et al., 2023). Additionally, ultra-high performance liquid chromatography coupled with mass spectrometry (UHPLC/MS) analysis identified key antioxidant compounds in *D. tortuosum* extract, such as phenolic acids, flavonoids (e.g., flavones, flavanones, and flavanols), and carotenoids, which collectively contributed to its significant ROS scavenging and anti-oxidative stress properties (Rodríguez et al., 2023). The chemical characterization of *South American Fabaceae Desmodium tortuosum* involved UHPLC/MS analysis to identify phenolic compounds, fourier transform infrared spectroscopy (FTIR) to detect functional groups like hydroxyl and carboxylic acids, nuclear magnetic resonance (NMR) spectroscopy to confirm glycosidic linkages and monosaccharide residues, high-performance anion-exchange chromatography with pulsed amperometric detection(HPAEC-PAD) to determine monosaccharide composition, gel permeation chromatography to measure molecular weight, atomic force microscopy (AFM) to analyze surface morphology, and TGA to assess thermal stability, providing a comprehensive understanding of its antioxidant properties (Rodríguez et al., 2023). Similarly, the chemical characterization of antioxidants in nine *Hypericum* species native to Greece, including endemic species like *H. cycladicum*, *H. fragile*, and *H. delphicum*, was conducted using liquid chromatography coupled with quadrupole time-of-flight high-resolution mass spectrometry (LC/Q-TOF/HRMS) analysis to identify and quantify compounds such as hypericin, quercetin, and flavonoids, while their antioxidant activity was evaluated through in vitro scavenging assays, providing a comprehensive understanding of their chemical composition and potential applications in functional foods and pharmaceuticals (Kakouri et al., 2023). In the chemical characterization of antioxidant compounds, high-performance liquid chromatography–electrospray ionization mass spectrometry(HPLC–ESI/MS) analysis of propolis from three Canadian regions (Saskatchewan, Ontario, and British Columbia) identified 21 compounds, including 18 polyphenols such as benzyl caffeate, pinocembrin, sakuranetin, and pinobanksin-3-acetate, with Ontario propolis showing the highest concentrations of key polyphenols, while British Columbia propolis contained unique compounds like gallic acid and chrysin (Al Naggar et al., 2016).

The importance of chemical characterization after extraction, purification, and isolation steps lies in its ability to provide detailed insights into the structural composition, functional groups, and molecular properties of the isolated compounds, which are critical for understanding their bioactivity and antioxidant potential (Rawat et al., 2023). This step ensures the purity, stability, and bioavailability of the compounds, enabling accurate assessment of their antioxidant properties through assays like DPPH, ABTS, and FRAP. By identifying specific compounds such as phenolic acids, flavonoids, and carotenoids, chemical characterization helps establish a structure–activity relationship, which is essential for optimizing their use in functional foods, nutraceuticals, and pharmaceuticals. Without this step, the efficacy and safety of the antioxidants cannot be fully validated, limiting their potential applications in combating oxidative stress-related diseases.

## Synthesis, encapsulation and delivery systems of antioxidant nanoparticles

Daré and Lautenschlager (2025) explored the growing potential of antioxidant nanoparticles in addressing oxidative stress-related diseases. They emphasized how these nanomaterials can enhance the bioavailability, stability, and targeted delivery of antioxidants. The review covered various nanoparticle types-including Prussian blue, gold, and cerium oxide-that possess intrinsic antioxidant properties via mechanisms such as redox activity and ROS scavenging. Their therapeutic applications were particularly noted in conditions like cardiovascular diseases and neurotoxicity, where they demonstrate protective effects against oxidative damage. However, the authors also highlighted concerns about the toxicity risks associated with nanoparticle physicochemical properties, calling for further research to ensure their safety and clinical efficacy. Similarly, Cortesi and Sguizzato (2024) reviewed a broad range of antioxidant nanoparticle applications across biomedicine, cosmetics, and the food industry. Their focus was on lipid-based and polymeric nanoparticles, which significantly improve the solubility, stability, and targeted release of antioxidants such as resveratrol, α-bisabolol, and vitamin E. The review introduced innovative formulations-including solid lipid nanoparticles (SLNs), nanoemulsions, and inorganic nanoclusters-demonstrating efficacy in managing conditions like inflammation, diabetes, and neurodegenerative disorders. The authors underscored how nanoparticle design parameters, such as surface functionalization and lipid composition, directly impact therapeutic outcomes, offering versatile strategies for controlled antioxidant delivery.

The integration of nanotechnology into antioxidant research has revolutionized the field. Nanoparticles exhibit unique physicochemical properties-such as high surface area-to-volume ratios, tunable surface chemistry, and catalytic activity-making them highly effective in ROS scavenging and oxidative stress mitigation. These advantages have led to their widespread application in biomedical and therapeutic contexts. Notably, green synthesis methods, particularly those using plant extracts, are gaining traction due to their sustainability and the incorporation of natural phytochemicals that enhance antioxidant activity (Mohanta et al., 2017; Peng et al., 2024). Table 2 summarizes key findings from these studies, emphasizing the diverse roles of nanoparticles in antioxidant assays.

**Table 2:** Key findings of nanoparticles in antioxidant assays

Type of Nanoparticle	Key Findings	References
**Metal Nanoparticles**		
Gold (Au) NPs	Biomedical applications of nanoparticles include their use in biosensors, drug delivery, and cancer therapy, as well as their antioxidant activity. Gold nanoparticles (AuNPs), when functionalized with antioxidants (e.g., Trolox), show enhanced radical scavenging activity and can mimic antioxidant enzymes, providing protection against oxidative stress. Additionally, AuNPs functionalized with cowpea protein are utilized for electrochemical biosensors, further expanding their potential in biomedical applications	Cortesi & Sguizzato, 2024; Sundaram Sanjay & Shukla, 2021; Konopko et al., 2019
Silver (Ag) NPs	Scavenges free radicals, reduces oxidative stress, and protects cells from damage. Silver nanoparticles (AgNPs) synthesized from plant extracts exhibit strong antioxidant properties. These nanoparticles effectively scavenge free radicals and reduce oxidative stress, which contributes to diabetic wound healing	Sundaram Sanjay & Shukla, 2021; Kumar et al., 2020; Cortesi & Sguizzato, 2024
Zinc (Zn) NPs	Wound Healing: Promotes angiogenesis and collagen synthesis while reducing oxidative stress Antimicrobial Coatings: Scavenges ROS and inhibits microbial growth, showing potent activity against E. coli and strong ROS scavenging effects Cancer Therapy: Induces ROS-mediated apoptosis in cancer cells	Cortesi & Sguizzato, 2024
Copper (Cu)	Copper nanoparticles (CuNPs) synthesized from plant extracts exhibit significant antioxidant, antimicrobial, and antidiabetic properties. CuNPs are capable of inhibiting lipid peroxidation and reducing oxidative stress, which is particularly beneficial in diabetic conditions	Cortesi & Sguizzato, 2024; Sundaram Sanjay & Shukla, 2021
Manganese Nanoparticles (MnNPs)	Neuroprotection: Mimics SOD scavenges superoxide radicals and reduces oxidative stress in neural tissues Cardiovascular Protection: Reduces oxidative stress in heart tissues, promoting cardiovascular health Antioxidant Therapy in Cancer: Induces ROS-mediated apoptosis in cancer cells, offering potential therapeutic benefits in cancer treatment	Cortesi & Sguizzato, 2024; Sundaram Sanjay & Shukla, 2021
Platinum (Pt) Nanoparticles	Platinum nanoparticles (PtNPs) exhibit high catalytic activity and the ability to scavenge ROS. They mimic the functions of SOD and CAT, effectively protecting cells from oxidative stress and aging. Due to these properties, PtNPs are increasingly utilized in antioxidant, anticancer, and anti-aging applications	Sundaram Sanjay & Shukla, 2021
**Metal Oxide Nanoparticles**		
Ceria/Cerium Oxide (CeO₂)	Neuroprotection, ocular protection, cardiovascular protection, and cancer therapy Cerium oxide nanoparticles (CeO₂) mimic SOD and CAT, effectively scavenging ROS CeO₂ nanoparticles protect against oxidative stress in neurodegenerative diseases, radiation-induced damage, and ischemia/reperfusion injury	Sundaram Sanjay & Shukla, 2021; Ge et al., 2022; Cortesi & Sguizzato, 2024
Yttria (Y₂O₃)	Antioxidant and Biomedical Applications Yttrium oxide nanoparticles (Y₂O₃) exhibit strong antioxidant properties, similar to cerium oxide (CeO₂), by protecting cells from oxidative stress and promoting wound healing	Sundaram Sanjay & Shukla, 2021
Zinc Oxide (ZnO) NPs	Zinc oxide nanoparticles (ZnO NPs) exhibit strong antioxidant activity by scavenging free radicals and reducing oxidative stress. They have shown potential in diabetes management and various biomedical applications, including cytotoxicity in zebrafish oocytes. ZnO NPs induce autophagy and apoptosis by generating ROS in maturing oocytes, contributing to their antioxidant, antidiabetic, and antibacterial properties Additionally, ZnO NPs are widely used in cosmetics, such as sunscreens and anti-aging creams, due to their ROS scavenging activity. The incorporation of Schiff base ligands and metal complexes has been shown to enhance their antioxidant properties	Cortesi & Sguizzato, 2024; Sundaram Sanjay & Shukla, 2021; Ge et al., 2022; Kumar et al., 2020; Ibrahim et al., 2025
Iron Oxide (Fe₂O₃)	Applications: Drug delivery, antioxidant activity, and environmental remediation Iron Oxide Nanoparticles (Fe₂O₃ & Fe₃O₄): Fe₂O₃ nanoparticles functionalized with antioxidants (e.g., gallic acid) exhibit enhanced radical scavenging activity and protect cells from oxidative stress Fe₃O₄ nanoparticles synthesized using plant extracts demonstrate significant antioxidant activity by scavenging free radicals and reducing oxidative stress	Sundaram Sanjay & Shukla, 2021; Ge et al., 2022; Kumar et al., 2020
Titanium Dioxide (TiO₂) NPs	Applications in Cosmetics, Sunscreens, and Food Packaging Titanium dioxide (TiO₂) nanoparticles enhance UV protection and reduce lipid peroxidation. However, their interaction with polyphenols may decrease bioavailability	Ge et al., 2022; Cortesi & Sguizzato, 2024
Copper Oxide (Cu₂O)	Cu₂O nanoparticles synthesized from plant extracts exhibit antioxidant activity comparable to ascorbic acid, effectively reducing oxidative stress. These nanoparticles demonstrate ROS scavenging activity. Additionally, Schiff base ligands and their metal complexes further enhance the antioxidant properties	Kumar et al., 2020 ; Ibrahim et al., 2025
**Polymeric Nanoparticles**		
Polymeric Nanoparticles	Drug Delivery and Antioxidant Activity Polymeric nanoparticles (e.g., PLGA, chitosan) are widely used for drug delivery and the controlled release of antioxidants. These nanoparticles enhance the bioavailability and stability of encapsulated antioxidants, protecting them from degradation. Additionally, they help combat oxidative stress and improve therapeutic efficacy. Polymeric nanoparticles are particularly beneficial for targeted drug delivery in treating oxidative stress-related diseases	Sundaram Sanjay & Shukla, 2021; Cortesi & Sguizzato, 2024; Khan et al., 2025
Poly(lipoic acid)-based NPs	Cardiovascular Protection Protects cardiomyocytes from ROS-induced damage during ischemia/reperfusion	Cortesi & Sguizzato, 2024
**Lipid-Based Nanoparticles**		
Nanoemulsions	Food, cosmetics, and drug delivery: Nanoemulsions enhance the solubility and bioavailability of lipophilic antioxidants, thereby improving their efficacy in treating oxidative stress-related diseases. In Parkinson's disease models, quercetin-loaded nanoemulsions have shown neuroprotective effects. These nanoemulsions reduce ROS levels and improve mitochondrial function in Caenorhabditis elegans. Additionally, nanoemulsions enhance the solubility and stability of antioxidants, improving their bioavailability. They are widely used in both topical and oral delivery systems for antioxidants targeting oxidative stress-related diseases	Cortesi & Sguizzato, 2024; Khan et al., 2025; Sundaram Sanjay & Shukla, 2021
SLNs and NLCs	Drug Delivery and Cosmetic Applications SLNs and NLCs are used to encapsulate antioxidants, enhancing their stability and bioavailability. These nanoparticles are widely applied in skincare products and nutraceuticals	Cortesi & Sguizzato, 2024
Essential Oil-loaded NPs	Cosmetic Applications (e.g., acne treatment): Inhibit Cutibacterium acnes and scavenge ROS, protecting skin cells	Cortesi & Sguizzato, 2024
Mitochondria-targeting Nanoemulsions	Cancer Treatment Inhibits tumor growth in 3D models and zebrafish larvae	Cortesi & Sguizzato, 2024
**Carbon-Based Nanoparticles**		
Carbon-Based Nanoparticles	Radical Scavenging and Enzyme-Mimetic Activity Carbon dots and fullerenes function as"radical sponges,"effectively scavenging ROS and providing protection against oxidative stress. Due to their strong antioxidant properties, they are widely utilized in neuroprotection and cancer therapy	Innocenzi & Stagi, 2023; Cortesi & Sguizzato, 2024
Fullerenes (C₆₀)	Neuroprotection, Liver Protection, and Antioxidant Activity Fullerenes act as"radical sponges,"effectively scavenging free radicals and reducing oxidative stress. Water-soluble fullerenes have shown promising potential in protecting against liver damage and neurodegenerative diseases	Sundaram Sanjay & Shukla, 2021
**Other**		
Vesicles (Bilosomes)	Anti-inflammatory and antioxidant applications: Bilosomes improve the solubility and stability of antioxidants like budesonide, showing potential in reducing inflammation and oxidative damage in intestinal cells Drug delivery and antioxidant activity: Bilosomes enhance the delivery of antioxidants, offering benefits in reducing inflammation and oxidative damage	Cortesi & Sguizzato, 2024
α-Bisabolol-loaded NPs	Pharmaceutical, cosmetics, and food industries: Improve stability and antioxidant activity; exhibit antibacterial effects	Cortesi & Sguizzato, 2024
Silica (SiO₂)	Drug Delivery and Antioxidant Activity Silica nanoparticles (SiO₂) functionalized with antioxidants, such as gallic acid, exhibit enhanced radical scavenging activity. These SiO₂ nanoparticles play a crucial role in protecting against oxidative stress	Sundaram Sanjay & Shukla, 2021
Resveratrol-loaded NPs	Ocular Delivery for Inflammation and Oxidative Stress-Related Diseases: The goal is to prolong corneal residence time and enhance therapeutic effects	Cortesi & Sguizzato, 2024
Vitamin B1-stabilized Gold Nanoclusters	Antioxidant Enhancement Enhancement of antioxidant properties compared to pure Vitamin B1	Cortesi & Sguizzato, 2024
Selenium (Se)	Nutraceuticals, cancer therapy, and antioxidant activity: Se nanoparticles exhibit high antioxidant activity by enhancing GPx activity and scavenging free radicals	Ge et al., 2022; Kumar et al., 2020
Hybrid Nanoparticles	Hybrid nanoparticles (e.g., metal-polymer composites) exhibit synergistic antioxidant effects, combining multiple materials to enhance antioxidant activity and enable targeted delivery for biomedical applications	Cortesi & Sguizzato, 2024; Khan et al., 2025

Subroto et al. (2023) provided an in-depth analysis of SLNs as delivery vehicles for active and antioxidant compounds. Their review highlighted SLNs’ ability to improve the stability, bioavailability, and controlled release of both hydrophobic and hydrophilic antioxidants, including polyphenols, flavonoids, and vitamins. Key formulation variables-such as lipid matrix selection, surfactant type, and fabrication method-significantly influenced SLN performance. Among preparation techniques, high-shear homogenization combined with ultrasonication emerged as the most effective. The review also addressed SLNs’ applications in food, pharmaceuticals, and cosmetics, while noting ongoing challenges, particularly in optimizing entrapment efficiency for polar compounds. Future directions include improving scalability, biocompatibility, and clinical translation.

Maraveas et al. (2021) contributed a comprehensive overview of antioxidant polymers, focusing on sustainable monomers, green synthesis techniques, and cross-sectoral applications. They highlighted bio-based polymers such as lignin, dopamine/polydopamine, inulin, quercetin, and terpenes for their free radical scavenging capacity, biocompatibility, and potential use in food packaging, drug delivery, and antimicrobial treatments. The review also examined advanced polymerization methods like reversible-addition-fragmentation chain-transfer (RAFT), atom transfer radical polymerization (ATRP) and cost-effective production from agro-waste. A key insight was the comparison between synthetic and natural antioxidants, showing the superiority of bio-based materials in terms of sustainability and toxicity reduction.

Lipid-based nanocarriers have gained significant attention in antioxidant delivery due to their ability to enhance the stability, bioavailability, and therapeutic efficacy of poorly soluble compounds. Gunawan and Boonkanokwong (2024) evaluated both SLNs and nanostructured lipid carriers (NLCs) for oral delivery of antioxidant nutraceuticals such as curcumin, β-carotene, and quercetin. These systems protect antioxidants from gastrointestinal degradation, improving solubility and absorption. NLCs, composed of a blend of solid and liquid lipids, demonstrated superior performance over SLNs in terms of higher drug-loading capacity, enhanced stability, and sustained release. While commercial application remains limited, the incorporation of natural antioxidant-rich oils like olive and pomegranate further enhances therapeutic potential for oxidative stress-related conditions. Similarly, Akanchise et al. (2025) investigated the self-assembly of natural lipids and phytochemicals—including curcumin, vitamin E, and coenzyme Q10—into lyotropic liquid crystalline nanostructures (e.g., lamellar, inverted hexagonal, and cubic phases). Through techniques such as synchrotron X-ray scattering and cryo-TEM, they demonstrated that lipid composition and PEGylated amphiphiles (e.g., Pluronic F127, TPGS-PEG1000) play crucial roles in stabilizing these nanostructures and enhancing antioxidant efficacy. These nanocarriers showed low cytotoxicity in SH-SY5Y neuroblastoma cells and controlled biodegradability, indicating promise for multifunctional therapeutic delivery. Complementing these findings, Esposito (2022) reviewed various lipid-based nanostructures—including liposomes, ethosomes, SLNs, and NLCs—for delivering antioxidants such as pepper extracts, mangiferin, and phenolics. These systems improved antioxidant stability, controlled release, and cellular uptake, with liposomes effectively reducing intracellular ROS and ethosomes enhancing mangiferin skin penetration, highlighting their applicability in both pharmaceutical and cosmetic formulations.

Khan et al. (2025) provided a broad classification of nanoantioxidants based on origin (natural vs. synthetic), composition (metallic, polymeric, carbon-based), and structure, highlighting their enhanced bioavailability, stability, and therapeutic efficacy. Their review addressed sustainable synthesis approaches, key characterization techniques such as FTIR, zeta potential, SEM, and DSC, and a range of biological assays used to evaluate antioxidant activity. While they emphasized the promising therapeutic potential of nanoantioxidants, they also noted the need to overcome formulation and translational barriers for clinical applications. Expanding on the role of carbon-based nanoantioxidants, Wang et al. (2025) explored the applications of carbon dots (CDs) in the food industry, citing their exceptional ROS scavenging capabilities, biocompatibility, and customizable synthesis via hydrothermal or microwave-assisted methods. CDs were proposed as additives for food packaging, edible coatings, and nutrient preservation, with the potential for machine learning integration in their design, though the need for comprehensive safety evaluations was emphasized. Similarly, Innocenzi and Stagi (2023) discussed the dual oxidant–antioxidant nature of CDs, which can both generate ROS under UV/visible light and scavenge radicals, depending on environmental conditions. Their review encompassed graphene quantum dots (GQDs), carbon quantum dots (CQDs), and carbonized polymer dots (CPDs), emphasizing the importance of tailoring surface functionalities for specific biomedical and environmental applications.

In a comparative analysis of carbon nanomaterials, Li et al. (2014) evaluated their utility as carriers for catalase (CAT), a model antioxidant enzyme. Fullerenes (0D), carbon nanotubes (1D), graphene oxide (2D), and graphene aerogels (3D) exhibited varying encapsulation efficiencies and release behaviors, with graphene aerogels offering the most sustained release, indicating their suitability for lung-targeted antioxidant therapies. Sindhu Priya and Udaya Prakash (2023) studied carbon nanoparticles (CNPs) derived from *Eichhornia crassipes* for their antioxidant and cytotoxic potential. Although the CNPs showed limited antioxidant activity (indicated by high IC₅₀ values), they were non-toxic to A549 lung cancer cells, suggesting promise in cosmetic and biomedical applications where biocompatibility is prioritized over potency. In the energy sector, Kwon et al. (2022) reviewed the use of antioxidants in enhancing polymer electrolyte membrane (PEM) durability in fuel cells, with strategies including cerium-based additives, ROS scavengers, and hydrogen peroxide decomposition catalysts. They emphasized the role of functionalization and compatibility assessments using advanced characterization tools like XAFS and XRF to ensure electrochemical stability. Likewise, Lu et al. (2018) reviewed nanoparticle-immobilized antioxidants (NP-AOs) integrated into polymers, which prevent migration and extraction while maintaining antioxidant activity. Nanomaterials such as silica, carbon nanotubes, and graphene oxide improved thermal and mechanical performance, though optimization of dispersion and antioxidant loading remains a key challenge for scalable industrial application.

Gold nanoparticles (AuNPs) have emerged as promising antioxidants due to their unique physicochemical properties, including minimal toxicity, ease of synthesis, and high detectability (Suliasih et al., 2024). Their antioxidant activity primarily arises from their ability to scavenge ROS through catalytic surface reactions, effectively mimicking enzymatic antioxidants such as CAT and SOD. Beyond food science, AuNPs find applications in anti-aging and anti-inflammatory therapies, wound healing, and the management of oxidative stress-related diseases such as diabetes and atherosclerosis. Nevertheless, challenges remain-particularly regarding scalability, stability in biological systems, and potential long-term toxicity-that must be addressed to fully optimize their clinical and industrial applications. Future advancements in green synthesis methods and surface functionalization hold promise for enhancing both their therapeutic potential and safety profiles (Kumar et al., 2020). Dehghani et al. (2023) demonstrated the green synthesis of gold nanoparticles (AuNPs) using *Glaucium flavum* leaf extract, producing stable, spherical nanoparticles averaging 32 nm in size. These AuNPs exhibited strong antioxidant and antiviral activities against the influenza virus, with low cytotoxicity and good biocompatibility. The study highlights the potential of eco-friendly synthesized AuNPs as effective nanoplatforms for biomedical applications. Complementing these advances, Feng et al. (2017) introduced a simple colorimetric assay for detecting GSH in cells by leveraging gold nanoclusters (GSH-AuNCs) with peroxidase-like activity. Since elevated GSH levels are characteristic of cancer cells, this assay distinguishes cancerous from normal cells based on GSH’s inhibition of the peroxidase-like oxidation of 3,3′,5,5’-tetramethylbenzidine (TMB), which produces a blue color in the presence of H₂O₂. Higher GSH concentrations weaken this colorimetric reaction, causing fading. Notably, this enzyme-free, rapid, and cost-effective assay achieves a detection limit of 420 nM and a linear range of 2–25 μM, outperforming traditional techniques such as HPLC and fluorescence. Its visual readout and minimal instrumentation requirements make it a promising tool for early cancer diagnosis and biomedical research, highlighting the potential of nanocluster-based biosensors for sensitive and reliable antioxidant detection.

More recently, Zhao et al. (2021) developed visible light-responsive hybrid nanozymes (Au/NC) by combining gold nanoparticles with nitrogen-doped carbon dots, creating a system capable of dynamically regulating redox balance. These nanozymes exhibit dual functionality, acting as antioxidants under normal conditions by scavenging ROS and mimicking SOD activity, while under visible light irradiation, they switch to a pro-oxidant mode by generating ROS such as hydroxyl radicals and singlet oxygen, thereby enhancing oxidase-like activity. This light-switchable behavior results from the synergy between carbon dot photoexcitation and gold surface plasmon resonance. The study demonstrates practical applications in controlling oxidation processes like Monascus pigment degradation and Fe^2^⁺ oxidation, providing a precise, stimuli-responsive approach to modulate biological redox states in both therapeutic and industrial contexts.

Meena Kumari et al. (2015) demonstrated a green synthesis method for Au–Ag bimetallic nanoparticles (BMNPs) using pomegranate fruit juice, which acted as both a reducing and stabilizing agent. The study revealed that varying the Au/Ag molar ratios produced alloy and core–shell nanostructures, confirmed by UV- Vis spectroscopy, Transmission Electron Microscopy (TEM), and X-ray Diffraction (XRD) analysis. These BMNPs exhibited excellent catalytic activity in degrading nitrophenols (4-NP > 2-NP > 3-NP) and methyl orange, following pseudo-first-order kinetics. Additionally, they enhanced thermal conductivity in nanofluids and displayed strong antioxidant properties, effectively scavenging nitric oxide (ON^•^) and hydroxyl (HO^•^) radicals, comparable to gallic acid. This work highlights the multifunctional applications of biosynthesized BMNPs in catalysis, thermal engineering, and biomedicine.

Silver nanoparticles have been extensively studied for their antioxidant properties. For instance, AgNPs synthesized using *Erythrina suberosa* leaf extract demonstrated significant antioxidant activity, as assessed by the DPPH (2,2-diphenyl-1-picrylhydrazyl) assay. The DPPH assay involves the reduction of the DPPH radical (a stable free radical) by antioxidants, leading to a color change from purple to yellow. The chemical reaction is as follows:

$$\mathrm{DPPH}^\cdot+\mathrm{Antioxidant}\rightarrow\mathrm{DPPH}-\mathrm H$$

Similarly, green synthesis of AgNPs using *Dracocephalum kotschyi* extract showcased antimicrobial, antioxidant, and anticancer potentials. The strong antioxidant activity of these nanoparticles was attributed to the phytochemicals present in the plant extract, underscoring the dual benefits of green synthesis and enhanced bioactivity (Peng et al., 2024).

Zinc oxide nanoparticles synthesized using grape seed extract have demonstrated significant antioxidant and anti-inflammatory activities. These nanoparticles were effective in scavenging free radicals, as evidenced by H₂O₂ scavenging and ABTS (2,2'-azino-bis (3-ethylbenzothiazoline-6-sulfonic acid)) quenching assays. The chemical reactions involved in these assays are:

$${\mathrm H}_2{\mathrm O}_2\;+\;\mathrm{Antioxidant}\;\rightarrow\;{\mathrm H}_2\mathrm O\;+\;{\mathrm O}_2$$

​ 

$$\mathrm{ABTS}^{\cdot+}+\;\mathrm{Antioxidant}\;\rightarrow\;\mathrm{ABTS}$$

The study also highlighted the potential of ZnO NPs in therapeutic applications due to their biocompatibility and enhanced antioxidant properties (N et al., 2024).

Iron oxide nanoparticles mediated by *Coleus amboinicus* extract exhibited notable free radical scavenging properties. The antioxidant activity of these nanoparticles increased with concentration, suggesting their potential in therapeutic applications. The study emphasized the role of plant-mediated synthesis in enhancing the bioactivity of FeONPs (Kunjan et al., 2024). The scavenging of ROS by FeONPs can be represented as:

$$\mathrm{Fe}^{2+}+{\mathrm H}_2{\mathrm O}_2\rightarrow\mathrm{Fe}^{3+}\;+\;\mathrm{HO}^\cdot+\;\mathrm{OH}^-\;+\;\mathrm{Fe}^{2+}$$

$$\mathrm{Fe}^{3+}+\mathrm{Antioxidant}\rightarrow\mathrm{Fe}^{2+}+\;\mathrm{Oxidized}\;\mathrm{Antioxidant}$$

Selenium Nanoparticles (SeNPs) synthesized from *Theobroma cacao* bean shell extract showed substantial antioxidant activity. The presence of natural bioactive compounds in the extract likely contributed to the enhanced antioxidant properties of the nanoparticles. The antioxidant mechanism of SeNPs involves the reduction of ROS through the following reaction:

$$\mathrm{Se}^0\;+\;\mathrm{ROS}\rightarrow{\mathrm{SeO}}_2\;+\;{\mathrm H}_2\mathrm O$$

### Elemental selenium (Se⁰)

This study highlighted the potential of SeNPs in addressing oxidative stress-related disorders (Shanmugam et al., 2023).

Cobalt Nanoparticles (CoNPs) have also gained attention for their antioxidant properties. A study by Umar et al. (2024) investigated the dose-dependent effects of cobalt carbonate nanoparticles (CoCO₃ NPs) on albino mice. The results revealed that the activities of CAT and SOD increased in a dose-dependent manner, indicating an adaptive response to oxidative stress. However, peroxidase (POD) activity decreased at higher doses, suggesting potential inhibitory effects. Hematological analysis showed significant alterations in monocyte and platelet counts, while histopathological examination revealed liver and kidney damage at higher doses (Umar et al., 2024). Additionally, green synthesis of CoNPs using *Millettia pinnata*, *Butea monosperma*, and *Madhuca indica* extracts demonstrated significant antioxidant activity, with *Madhuca indica*-derived CoNPs showing the highest inhibition of 87.67% in the DPPH assay (Ryntathiang et al., 2024). Cobalt oxide nanoparticles (Co₃O₄ NPs) synthesized using *Curcuma longa* root extract also exhibited significant antioxidant, antimicrobial, and anticancer properties, further highlighting their potential in biomedical applications (Shanmuganathan et al., 2023).

The unique properties of nanoparticles have also been leveraged to develop innovative techniques for antioxidant detection. For example, iron oxide nanoparticles have been utilized to enhance the sensitivity of ABTS assays, providing a more efficient means of quantifying antioxidant capacity. This approach leverages the catalytic properties of nanoparticles to improve the detection of antioxidants, making it a valuable tool in both research and industrial applications (Sloan‐Dennison et al., 2017; Sharpe et al., 2013). The catalytic reaction can be represented as:

$$\mathrm{ABTS}^{\cdot+}\;+\;{\mathrm{Fe}}_3{\mathrm O}_4\;\rightarrow\;\mathrm{ABTS}\;+\;{\mathrm{Fe}}_3{\mathrm O}_4^\cdot$$

Additionally, a novel assay using silver nanoparticles has been developed for the measurement of antioxidants in distilled spirits. This method relies on the interaction between antioxidants and silver nanoparticles, leading to measurable changes in nanoparticle properties, and offers a rapid and sensitive approach for antioxidant quantification (Bukovsky-Reyes et al., 2018). The reaction mechanism involves the reduction of Ag⁺ ions by antioxidants:

$$\mathrm{Ag}^++\mathrm{Antioxidant}\rightarrow\mathrm{Ag}^0+\mathrm{Oxidized}\;\mathrm{Antioxidant}$$

The antioxidant activity of nanoparticles is primarily mediated through the scavenging of ROS and the enhancement of endogenous antioxidant enzymes such as CAT and superoxide dismutase (). The presence of phytochemicals in plant extracts used for green synthesis further enhances the antioxidant properties of nanoparticles by stabilizing free radicals and preventing oxidative damage. However, higher doses of nanoparticles may lead to inhibitory effects on certain antioxidant enzymes, such as peroxidase (POD), and cause organ damage, as observed in albino mice treated with cobalt carbonate nanoparticles (Umar et al., 2024).

## In-situ models and their advances in antioxidant assays

In-situ methods for antioxidant detection enable real-time, on-site analysis without extensive sample preparation, making them valuable tools in food quality control, biomedical research, and environmental monitoring. While Table 3**.** Provides an overview of in-situ antioxidant assays. These techniques assess antioxidant activity by measuring redox reactions directly within the sample matrix, offering advantages such as rapid results, minimal reagent consumption, and portability. Among these, electrochemical techniques have emerged as particularly powerful due to their speed, simplicity, and suitability for analyzing complex matrices. Notably, they allow direct measurement of TAC (AOxC) without requiring reactive species (Haque et al., 2021).

**Table 3:** Overview of in-situ techniques used for antioxidant capacity determination

Method	Principle	Advantages	Limitations	Typical Applications	References
Cyclic voltammetry (CV)	**Measures redox behavior of antioxidants through cyclic voltammetry** Records redox current during potential cycling; peak current correlates with antioxidant concentration Determines oxidation potential (E < sub > p,a </sub >); lower E < sub > p,a </sub > values indicate stronger electron-donating (antioxidant) capacity Analyzes redox properties via voltage scanning and Faradaic current response. Key parameters include peak potential (E < sub > p </sub >), half-wave potential (E < sub > 1/2 </sub >), peak current (i < sub > p </sub >), and total charge (Q) from the integrated area under the curve	Rapid screening Identifies redox potentials Low sample volume (µL range) Fast (< 10 min/analysis) Low-cost (no reagents needed) pH-flexible (physiological to acidic conditions) Equal accuracy for all compounds (± 3 mV) Direct and rapid screening Provides fundamental redox properties (Ep, E1/2, ip, Q) Sensitive to electron transfer processes Minimal sample preparation Low cost and miniaturizable	Non-specific (measures total reducing capacity) Interference from electroactive non-antioxidants Non-specific: Detects all electroactive species Matrix interference: Sugars/polymers distort results No DPPH correlation for non-phenolics Electrode potential not absolute (reference-dependent) Solvent/electrolyte effects may interfere Passivating films may form on electrodes Lack of standardized correlation with other assays	Fruit teas, wines, plant extracts Phenolic antioxidants in spices/drugs (e.g., gallic acid, eugenol) Structure–activity studies (e.g., –OH/–OCH₃ substituent effects) Coffee, wines, beverages, fruit juices, oils Algae, propolis, herbal extracts, bee products, by-products	Hoyos-Arbeláez et al., 2017; Arteaga et al., 2012; Agregán et al., 2024
Differential pulse voltammetry (DPV)	Pulse-based potential sweep minimizes capacitive effects by sampling current before and after each pulse. Peak height is proportional to analyte concentration. Combines staircase scan with superimposed pulses to improve signal accuracy	High sensitivity (LOD: 10⁻⁷–10⁻⁸ M) Resolves overlapping peaks (50 mV separation) Higher sensitivity and resolution than CV Better signal-to-noise ratio Selective for complex mixtures	Limited to electroactive compounds Requires stable reference electrode Similar limitations as CV (solvent, reference dependence) Requires optimization of pulse parameters	Polyphenol quantification in various matrices including wines, teas, juices, fruits, spices, herbal extracts, honey, propolis, meats, medicinal plants, and fish oils	Hoyos-Arbeláez et al., 2017; Agregán et al., 2024
Square wave voltammetry (SWV)	Square-wave potential pulses combined with forward/reverse current subtraction. Net current improves sensitivity. Staircase potential ramp with superimposed square pulses. Current measured at the end of each pulse	Ultra-low LOD (40 nM for EGCG) Fast analysis (< 1 min) Background current suppression Highest sensitivity among voltammetric methods Fast analysis time Reduced capacitance current Suitable for mechanistic studies	Complex waveform optimization Electrode fouling in dirty matrices Complex waveform optimization Limited mechanistic insights compared to CV	Green/black teas, trans-resveratrol in wine, red wines, garlic, teas, edible oils, plant extracts, fruit extracts, saffron, coffee, hop/malt samples, vitamins in supplements	Hoyos-Arbeláez et al., 2017; Agregán et al., 2024
Chronoamperometry (CA)	Measures diffusion-controlled current following a potential step using the Cottrell equation to quantify analytes. It determines the oxidation current of ferrocyanide (Fe(CN)₆^4^⁻), which is produced when antioxidants reduce potassium ferricyanide (K₃[Fe(CN)₆]). The resulting current is directly proportional to the total antioxidant activity (AOA)	Direct kinetic data Combines with DPPH assay Unaffected by sample turbidity High sensitivity (LOD: 2 × 10⁻⁶ M for uric/ascorbic acids, 5 × 10⁻⁶ M for cysteine/GSH) Good agreement with certified potentiometry (R^2^ = 0.9980)	Requires precise timing Lower selectivity vs. pulse methods Requires mediator system (K₃[Fe(CN)₆]) Electrode surface stability affects results	Brandy authentication, trolox equivalence; blood plasma analysis, antioxidant capacity assays	Brainina et al., 2012 ; Hoyos-Arbeláez et al., 2017
Potentiometry	Measures the potential shift in the K₃[Fe(CN)₆]/K₄[Fe(CN)₆] redox mediator system following antioxidant addition. Antioxidant activity (AOA) is quantified using the Nernst equation based on the observed potential change	Simple instrumentation Validated for biological samples	Longer equilibration time (~ 20 min) Larger sample volumes may be needed	Biological fluids (e.g., blood plasma)	Brainina et al., (2012)

In situ methods for measuring antioxidant capacity in food, biological, and environmental samples benefit greatly from the use of electrochemical biosensors. These tools are essential for improving the understanding and optimization of detection protocols. As reviewed by Sukma et al. (2023) and Munteanu and Apetrei (2022), electrochemical techniques offer a practical and efficient alternative to traditional methods such as spectrophotometry and chromatography, which are often time-consuming and costly. Various types of electrochemical sensors-including carbon-based, metal-based, nanomaterial-modified, enzyme-based, and molecularly imprinted polymer (MIP)-based sensors-provide advantages such as high sensitivity, rapid response, and applicability in real-time food quality and health assessments. These sensors work by exploiting the redox properties of antioxidants, making them suitable for diverse sample matrices. Although challenges remain-such as enzyme stability, matrix interference, and scalability- recent advancements in nanomaterials and immobilization techniques show strong potential for developing portable, cost-effective, and high-throughput systems. The increased demand for antioxidant monitoring, particularly following the COVID-19 pandemic, underscores the importance of these technologies in addressing oxidative stress and supporting immune health. The operational principle of electrochemical methods is based on oxidation–reduction (redox) reactions of antioxidants, typically involving electron transfer (ET) mechanisms. These methods are suitable for detecting both polar and non-polar antioxidants, making them versatile for a wide range of applications (Bunaciu et al., 2016). Common techniques include cyclic voltammetry (CV), differential pulse voltammetry (DPV), and square wave voltammetry (SWV), each offering specific benefits in sensitivity and resolution (Jadon et al., 2017; Smolyaninov et al., 2017; Stevanović et al., 2020). Key analytical parameters include oxidation current, which reflects the current produced during antioxidant oxidation, and redox potential, which characterizes antioxidant electrochemical behavior. For instance, chronoamperometric techniques can determine total antioxidant concentration by measuring the oxidation current of ferrocyanide formed during reactions with antioxidants (Bunaciu et al., 2016).

Cyclic voltammetry (CV) is particularly useful for studying the oxidation–reduction behavior of antioxidants. It measures peak currents and potentials, which correlate with the concentration and type of antioxidant compounds present. For example, the anodic and cathodic peak currents can be directly linked to the concentration of phenolic compounds, known for their antioxidant properties (Barros et al., 2011; Jara-Palacios et al., 2020). CV's ability to provide detailed insights into the electrochemical behavior of antioxidants makes it a preferred method in this field (Suliborska et al., 2019; Tufan et al., 2014). Cyclic Voltammetry (CV): Measures redox reactions of antioxidants on an electrode surface. Potentiometry: Measures potential changes related to antioxidant concentration (Ye et al., 2020). In parallel, electrochemical techniques like cyclic voltammetry (CV) and amperometric sensing have gained widespread use in the food and nutraceutical industries for real-time assessment of antioxidant capacity. These methods offer rapid, sensitive, and cost-effective alternatives to traditional assays such as DPPH and oxygen radical absorbance capacity (ORAC), enabling on-site quality control, shelf-life prediction, and validation of antioxidant efficacy in functional foods and supplements (Suliasih et al., 2024).

Differential pulse voltammetry (DPV) and square wave voltammetry (SWV) offer enhanced sensitivity and resolution, enabling the detection of low concentrations of antioxidants in complex samples. For instance, DPV has been successfully used to measure the antioxidant capacity of natural compounds like polyphenols by analyzing their redox behavior (Gomes et al., 2015; Piszcz et al., 2013). Combining these electrochemical techniques with high-performance liquid chromatography (HPLC) further improves analysis by separating antioxidants before electrochemical evaluation, providing a comprehensive understanding of their antioxidant potential (Fu et al., 2020; Ziyatdinova & Kalmykova, 2023).

Electrochemical methods are also valuable for exploring the mechanisms behind antioxidant activity. By studying the electrochemical responses of antioxidants, researchers can gain insights into the kinetics and thermodynamics of their interactions with free radicals (Andrei et al., 2016; Jadon et al., 2017). Additionally, the use of modified electrodes, such as those incorporating graphene or other nanomaterials, enhances the sensitivity and selectivity of electrochemical sensors, making them suitable for real-time monitoring of antioxidant activity in food and biological samples (Kovachev et al., 2010).

Flow-injection analysis with amperometric detection (FIA-AD) is another innovative technique for rapidly and sensitively assessing antioxidant capacity. Amperometry and chronoamperometry detect changes in current due to antioxidant activity (Ye et al., 2020).

This method combines flow-injection analysis with amperometric detection, enabling continuous monitoring of antioxidant activity through the reduction of stable free radicals like DPPH (2,2-diphenyl-1-picrylhydrazyl) (Oliveira et al., 2016). The amperometric detection mechanism measures current changes as antioxidants react with DPPH, providing a quantifiable measure of antioxidant capacity (Andrei et al., 2014). FIA-AD is particularly advantageous for high-throughput analysis, making it ideal for applications in food chemistry and pharmacognosy, where rapid evaluation of antioxidant properties is essential for quality control and efficacy studies (Ribeiro et al., 2011). For example, it has been effectively used to assess the antioxidant activity of plant extracts, including traditional Chinese medicine formulations, showcasing its versatility (Li et al., 2016; Yang et al., 2016a, b).

FIA-AD can also be integrated with other analytical techniques, such as HPLC, to simultaneously separate, quantify, and assess the activity of antioxidant compounds. This dual approach improves the accuracy of antioxidant measurements and helps identify specific compounds responsible for the observed activity (Nuengchamnong & Ingkaninan, 2010; Smrke et al., 2013).

The electrochemical techniques like CV, DPV, SWV, and FIA-AD provide rapid, sensitive, and versatile methods for evaluating antioxidant activity. Their ability to analyze complex matrices and explore antioxidant mechanisms makes them invaluable tools in food science, pharmacology, and biomedical research (Bunaciu et al., 2016). These methods are particularly useful for studying polyphenolic compounds, plant extracts, and other antioxidants in both polar and non-polar matrices, offering significant advantages in terms of speed, cost-effectiveness, and sensitivity. However, they require careful calibration and standardization to avoid interference from other electroactive species in complex matrices (Bunaciu et al., 2016). Electrochemical methods are gaining traction due to their direct measurement of antioxidant concentration without the need for reference materials. They are instrumentally simple, cost-effective, and time-efficient, making them suitable for clinical and field applications. Used in food quality assessment (e.g., wine, fruits) and clinical monitoring of oxidative stress. Development of wearable sensors for real-time antioxidant monitoring (Ye et al., 2020).

The increasing demand for accurate, rapid, and portable antioxidant detection methods has driven the development of innovative techniques leveraging nanoparticle synthesis for real-time analysis. One such approach, the in-tube solid-phase microextraction coupled with capillary liquid chromatography and diode-array detection (IT-SPME-CapLC-DAD), utilizes silver nanoparticle (AgNP) formation as an indicator of antioxidant activity (Prieto-Blanco et al., 2023). This method operates on the principle that polyphenols (e.g., Trolox, chlorogenic acid) reduce Ag⁺ to Ag⁰, generating AgNPs whose localized surface plasmon resonance (LSPR) is monitored at 400–430 nm. The technique distinguishes between different AgNP populations, providing insights into their size, coating, and aggregation state. Optimized conditions, such as a basic medium (NaOH) and cationic surfactants (CTAC/CTAB), enhance AgNP stability, while temperature adjustments influence reaction kinetics. Quantitative analysis demonstrates linear relationships for Trolox (25–500 µM) and chlorogenic acid (up to 3 mM, LOD = 34 µM), with applications in dietary supplements (97–100% recovery) and TAC assessment. Compared to traditional UV–Vis and HPLC-ICP-MS methods, IT-SPME-CapLC-DAD offers real-time monitoring, speciation capability, and greener chemistry by avoiding toxic reagents (Prieto-Blanco et al., 2023).

In parallel, smartphone-based chemosensors using gold nanoparticles (AuNPs) have emerged as a portable and cost-effective alternative (Calabria et al., 2021). These sensors detect antioxidants through a colorimetric shift (red to blue) as Au (III) is reduced to AuNPs in hydrogel cartridges. The system leverages smartphone CMOS cameras and LED illumination for rapid TAC measurement in teas, olive oil, and beverages, correlating well with ORAC assays. Unlike lab-bound techniques (e.g., ABTS, DPPH), this approach is field-deployable, user-friendly, and low-cost, making it ideal for food quality control and consumer-level testing (Huang et al., 2016).

## In vitro models in analysis of antioxidant properties: methods, mechanisms and applications

In vitro antioxidant assays serve as essential tools for evaluating the efficacy of bioactive compounds through controlled experimental systems. These methods can be broadly classified into three categories: chemical (non-enzymatic), enzymatic, and cell culture-based assays, each offering distinct advantages for assessing antioxidant activity (Thakur and Kapila, 2017).

The mechanism of antioxidant action varies across different systems, with the choice of assays and underlying principles depending on the research question, sample nature, and desired outcome. Chemical assays primarily evaluate antioxidant capacity through two key mechanisms: radical scavenging and inhibition of oxidative damage. Radical scavenging assesses a compound's ability to neutralize ROS/RNS via hydrogen atom transfer (HAT) or single electron transfer (SET), as demonstrated in common assays like DPPH and ABTS. Unlike enzymatic antioxidants, which are substrate-specific, chemical antioxidants exhibit broad reactivity, allowing them to counteract diverse free radicals. HAT-based assays, such as the ORAC assay, measure an antioxidant's ability to quench radicals by donating a hydrogen atom. In contrast, SET-based assays determine the compound's capacity to transfer an electron, thereby reducing the radical species. Each mechanism has distinct principles, advantages, and limitations, making them suitable for different applications (Xiao et al., 2020; Danet, 2021a, b; Munteanu & Apetrei, 2021). Enzymatic assays focus on endogenous antioxidant systems, including SOD, CAT, and GPx. These enzymes play a critical role in cellular defense by targeting specific reactive species in cofactor-dependent reactions. Their high substrate specificity makes them valuable for studying targeted oxidative stress mitigation (Haida & Hakiman, 2019).

Cell culture-based assays provide a more biologically relevant platform by growing cells or tissues in controlled environments. These systems offer significant advantages, including minimal compound requirements which is ideal for early-stage research, and rapid, reproducible screening of antioxidant potential under standardized conditions. While simplified compared to in vivo models, cell cultures help bridge the gap between chemical tests and complex biological systems (Thakur & Kapila, 2017).

Collectively, in vitro assays enable cost-effective, high-throughput screening of antioxidant properties. However, combining multiple methods is recommended to account for different mechanisms of action and enhance biological relevance. These approaches lay the groundwork for further validation in preclinical and clinical studies, ensuring a comprehensive understanding of a compound's antioxidant potential (Danet et al., 2021b; Thakur & Kapila, 2017).

$$\mathrm X-\mathrm H\;+\;\mathrm Y^\bullet\rightarrow\;\mathrm X^\bullet\;+\;\mathrm Y-\mathrm H\quad\quad\quad\quad\quad\}\mathbf{HAT}$$

where X − H is the H-donor and Y^•^ is the radical acceptor.

$$\left.\begin{array}{r}\mathrm{Donor}\;(\mathrm D)\;\rightarrow\;\mathrm{Donor}^\bullet+\;(\mathrm D^{\bullet+})\;+\;e^-\\\mathrm{Acceptor}\;(\mathrm A)\;+\;e^-\;\rightarrow\;\mathrm{Acceptor}^{\bullet-}{(A^{\bullet-}})\\D\;+\;A\;\rightarrow\;D^{\bullet+}\;+\;A^{\bullet-}\;(\mathrm{General}\;\mathrm{Equation})\end{array}\right\}\quad\quad\quad\quad\quad\mathbf{SET}$$

The type of sample being analyzed significantly influences the choice of assay. For instance, the Folin-Ciocalteu (FC) assay is effective for measuring polyphenolic content in plant extracts, as many plant-derived antioxidants are polyphenols (Hemmateenejad et al., 2012). Conversely, assays like ABTS (2,2'-azino-bis (3-ethylbenzothiazoline-6-sulfonic acid) and FRAP are more versatile and can be applied to a broader range of food products, including those with complex matrices (Gülçın, 2020; Suleria et al., 2020). This versatility is critical in food science, where the antioxidant capacity of various components must be assessed to understand their health benefits fully (Cömert & Gökmen, 2017; Echegaray et al., 2021).

In biological systems, oxidants are referred to as prooxidants, which induce oxidative damage to nucleic acids, lipids, and proteins. Reductants, on the other hand, are termed antioxidants, as they neutralize pro-oxidants and form non-toxic or less harmful byproducts (Tomaz et al., 2021)*.*The broader definition of"antioxidant"refers to any substance (molecules, ions, or stable radicals) that significantly delays, prevents, or removes the oxidation of an oxidizable agent at relatively low concentrations by neutralizing free radicals, often acting as chain-breaking agents(Flieger et al., 2021; Halliwell, 1995; López-Alarcón & Denicola, 2013). Not all reductants are antioxidants; only compounds that protect biological targets qualify as antioxidants. This protection occurs through mechanisms such as ROS/RNS scavenging, reducing power, metal chelation, antioxidative enzyme activity, and oxidative enzyme inhibition. Methods for assessing scavenging and reducing capacities are widely discussed, whereas evaluating enzymatic activity or inhibition falls outside the scope of this discussion. Additional insights into oxidative stress biomarkers and in vivo antioxidant assessments are available in recent studies (Mandal et al., 2022; Shahidi, 2015).

Antioxidants can be classified into primary and secondary antioxidants based on their mechanism of action. Primary antioxidants inhibit oxidation by donating hydrogen atoms or accepting free radicals to form more stable radicals, thereby interrupting the propagation phase of oxidative chain reactions. Secondary antioxidants function through various mechanisms, such as metal ion binding, ROS scavenging, hydroperoxide conversion, UV absorption, or singlet oxygen deactivation (Aziz et al., 2019). The efficiency of antioxidant capacities is influenced by several factors, which can be categorized into structural properties, environmental conditions, concentration and localization, and reaction kinetics. Structural properties determine the reactivity of antioxidants to free radicals, while environmental conditions, such as temperature, substrate characteristics, and the presence of synergists or pro-oxidants, play a significant role in their performance. The concentration and localization of antioxidants, particularly their distribution within a system, especially at interfaces, also affect their effectiveness. Additionally, reaction kinetics, including the rate, thermodynamics, and ability to neutralize oxidants, are critical factors. Ultimately, the effectiveness of an antioxidant depends on its chemical structure, reaction kinetics, and suitability for specific applications (Munteanu & Apetrei, 2021).

Antioxidants (AOs) can be categorized into enzymatic and non-enzymatic systems, which correspond to cellular and non-cellular mechanisms, respectively. Enzymatic AOs include SOD, CAT, peroxiredoxins (Prx), thioredoxin reductase (TR), reduced GPx, and oxidized GR. Non-enzymatic AOs are classified into five main groups: 1) Endogenous low-molecular-weight AOs, such as free reduced GSH, uric acid (UA), lipoic acid (LA), coenzyme Q10 (CoQ10), bilirubin, methionine, and cysteine; 2) Exogenous low-molecular-weight AOs, like ascorbic acid (vitamin C), tocopherols (vitamin E), and carotenoids (beta-carotene and vitamin A); 3) Proteins, including albumin and cysteine-rich proteins; 4) Polypeptidic AOs, such as thioredoxins, glutaredoxins, and sulfiredoxins; and 5) Metal-binding proteins like ceruloplasmin and metallothioneins. Exogenous and endogenous antioxidants work together to counteract pro-oxidants, forming an interconnected network that reduces oxidative stress (Ialongo, 2017).

The reduction of RONS (reactive oxygen and nitrogen species) levels through scavenging by small-molecule antioxidants was once considered a primary indicator of their bioactivity, but this view is outdated. While numerous in vitro assays demonstrate the free radical scavenging activity of compounds like curcuminoids, resveratrol, cinnamic acids, lignans, and flavonoids, their relevance to in vivo conditions is limited due to the rapid reactivity and short lifespan of RONS. An exception is vitamin E, which effectively combats ROO• in biological membranes. Despite the limited efficiency of dietary antioxidants in reducing RONS levels, their role in forming secondary metabolites may warrant further exploration (Hunyadi, 2019). Not all reductants are antioxidants; only compounds that protect biological targets qualify as antioxidants. This protection occurs through mechanisms such as ROS/RNS scavenging, reducing power, metal chelation, antioxidative enzyme activity, and oxidative enzyme inhibition. Methods for assessing scavenging and reducing capacities are widely discussed, while evaluating enzymatic activity or inhibition falls outside this scope. Additional insights on oxidative stress biomarkers and in vivo antioxidant assessments are available in recent studies (Kumari & Shahidi, 2024; Mandal et al., 2022).

The terms used to explain antioxidant activity are dependent on the specific context. For example, the ability of an antioxidant to slow oxidation in a soft drink may differ from its ability to protect biomembranes from oxidative damage in vivo. Additionally, the efficacy of an antioxidant in protecting a food supplement may not correlate with its ability to extend the shelf life of a drug. Consequently, assays used to compare antioxidants for food preservation may differ from those aimed at improving drug shelf life. To be relevant to human oxidative stress, an optimal assay should focus on the effect of antioxidants on PUFA (polyunsaturated fatty acid) peroxidation in model membranes or serum/LDL samples. Most commonly used assays are suited for antioxidants that stabilize water-soluble compounds, while biologically relevant antioxidants require assays involving aggregated substrates (Chandimali et al., 2025; Oh et al., 2021).

When describing the factors affecting antioxidant activity, several key aspects must be considered. One major factor is the reactivity toward free radicals (FR^•^), which determines how effectively an antioxidant neutralizes free radicals. Another important aspect is the stoichiometric factor (n), which indicates the number of free radical molecules neutralized by each antioxidant molecule. Additionally, the liposolubility of the antioxidant, or its solubility in lipids, plays a crucial role in its ability to function effectively in lipid-rich environments. Lastly, the presence of secondary reactions, or additional reactions, can significantly influence the overall antioxidant activity (López-Alarcón & Denicola, 2013; Zhou et al., 2017). The mechanisms of antioxidant actions are complex and context-dependent, requiring careful selection of assays and consideration of various factors to accurately assess antioxidant capacity and efficacy. Table 4 provides an overview of in vitro antioxidant assays, including their principles, measurement methods, and biological relevance.

**Table 4:** Overview of in vitro antioxidant assays: principles, measurement methods, and biological relevance

Assay name	Principle	Polarity of antioxidants	Type of reaction	Probe measurement	Detection method	Kinetics/Endpoint	Biological relevance	Measured parameters	Reference(s)
DPPH (2,2-diphenyl-1-picrylhydrazyl) Radical Scavenging Assay	Measures the ability of antioxidants to neutralize the DPPH radical Indicates the antioxidant capacity of a substance Assesses the ability of antioxidants to donate hydrogen atoms or electrons Reduces DPPH radicals to DPPH-H	Hydrophobic: Soluble only in organic solvents Hydrophilic: Water-soluble Lipophilic: Fat-soluble Best for low-polarity compounds (e.g., phenolics)	Mixed (primary SET and secondary HAT)	Chromogen radical: 2,2-diphenyl-1-picrylhydrazyl	Scavenging of stable free radicals Spectrophotometry (517 nm) UV–Vis Spectrophotometry	End-point (after 30 min)	Determines antioxidant capacity Relevant for free radical-induced diseases	Radical Scavenging Activity (RSA)	Brand-Williams et al., 1995; Aruoma, 2003; Munteanu & Apetrei, 2021; Rumpf et al., 2023; Baliyan et al., 2022; Gulcin & Alwasel, 2023; Siddeeg et al., 2021
ABTS (2,2'-Azino-bis(3-ethylbenzothiazoline-6-sulfonic acid)) Radical Cation Scavenging Assay	Reduction of ABTS⁺ radicals by antioxidants Results in a color change from blue-green to colorless Reaction with organic radical cation Antioxidants interact with the pre-generated ABTS•⁺ radical cation Leads to decolorization	Hydrophilic & lipophilic	Mixed (primary SET and secondary HAT)	Decolorization of ABTS• + radical cation, measured by UV–VIS spectroscopy	Spectrophotometric measurement at 414–417 nm or 730–734 nm	End-point measurement (4–6 min), but kinetics can be monitored over time	Measures antioxidant capacity but does not estimate antioxidant reactivity or inhibition rates	TAC, stoichiometry of ABTS• + scavenging, and reaction pathways	Re et al., 1999; Xie & Schaich, 2014; Rumpf et al., 2023; Ilyasov et al., 2020
FRAP (Ferric Reducing Antioxidant Power) Assay	Reduction of Fe^3^⁺-TPTZ complex to Fe^2^⁺ produces a blue-colored complex. Antioxidants reduce Fe^3^⁺-TPTZ to Fe^2^⁺-TPTZ The reaction results in the formation of a blue-colored complex	Hydrophilic (only in aqueous solution)	SET	Chromogen radical: ferric tripyridyltriazine (Fe3 + -TPTZ) Fe^2^⁺-TPTZ complex	Measures reducing power Spectrophotometry (593 nm)	End-point	Measures antioxidant capacity, relevant to oxidative stress and related diseases (e.g., diabetes, Alzheimer’s)	Antioxidant capacity (µmol Fe^2^⁺/g sample) Total Antioxidant Units (TAUFe/μmol)	Benzie & Strain, 1996; Tian & Schaich, 2013; Rumpf et al., 2023; Siddeeg et al., 2021;Spiegel et al., 2020
ORAC Assay	Antioxidants inhibit fluorescein oxidation induced by peroxyl radicals Measures fluorescence decay due to oxidation of a probe (e.g., fluorescein), delayed by antioxidants Evaluates antioxidant capacity by assessing the inhibition of fluorescein (FLH) oxidation induced by AAPH-derived radicals Monitors the inhibition of fluorescein bleaching caused by ROO• to measure antioxidant capacity	Hydrophilic & Lipophilic	HAT	Bleaching of hydrophilic carotenoid derivative: Strong golden-yellow hue Fluorescence decay of probes like fluorescein: Fluorescein molecule fluoresces as long as it is protected from oxidation by antioxidants (AOs) in the sample Fluorescein fluorescence intensity	Fluorescence Spectroscopy: Measures oxygen radical absorbance Fluorescence Spectrophotometry: Excitation: 485 nm Emission: 520 nm	Area Under the Curve (AUC): Measures the total exposure of a substance over time Kinetics: Describes how the substance is absorbed, distributed, metabolized, and eliminated Multiple Points: Data points are collected at various time intervals Continuous Monitoring: Ongoing observation for a 120-min period 120 Minutes: The total time span for continuous kinetic monitoring	Measures antioxidant capacity in biological systems, including food and pharmaceuticals, for chain-breaking effects	Assesses the ability to neutralize free radicals R0 (Initial Consumption Rate): The rate at which a substance is consumed initially in a reaction BDE (Bond Dissociation Energy): Energy required to break a bond in a molecule	Ou et al., 2002; Magalhães et al., 2008; Figueroa et al., 2023; Asma et al., 2024; Siddeeg et al., 2021
CUPRAC (Cupric Ion Reducing Antioxidant Capacity) Assay	Reduction of Cu^2^⁺-neocuproine complex to Cu⁺ leads to color change	Both hydrophilic and lipophilic	SET	Chromogen radical: bathocuproine:Cu2 + 2:1 chelating complex	Measures reducing power Spectrophotometry (450 nm)	End-point (single time point)	Measures TAC in biological fluids and foods, relevant to oxidative stress prevention	Antioxidant capacity (µmol Trolox/g sample)	Apak et al., 2004; Kılınçer et al., 2022; Siddeeg et al., 2021
β-Carotene Bleaching Assay	Antioxidants prevent oxidative bleaching of β-carotene by free radicals Measures inhibition of β-carotene oxidation by antioxidants Free radicals from linoleic acid oxidize β-carotene, causing bleaching Antioxidants neutralize radicals, slowing the bleaching process	Lipophilic	HAT	β-carotene	Absorbance measured at 470 nm using a spectrophotometer (TU-1800) Blank consisted of the emulsion without β-carotene	Kinetics	Simulates lipid peroxidation, relevant to oxidative stress in biological systems	% Inhibition of β-carotene oxidation: The percentage of inhibition observed in the oxidation of β-carotene Bleaching rate (R): The rate at which the β-carotene undergoes bleaching due to oxidation Antioxidant activity (AA): The ability of a substance to prevent the oxidation of β-carotene, typically measured through its effect on the bleaching rate IC50: The concentration required for 50% inhibition of β-carotene oxidation	Miller et al., 1993; Yang et al., 2008a, b; Prieto et al., 2012; Christodoulou et al., 2022; Dawidowicz & Olszowy, 2015; Siddeeg et al., 2021
TBARS (Thiobarbituric Acid Reactive Substances) Assay	Measures MDA levels as an indicator of lipid peroxidation		Mixed (primary SET and secondary HAT)		Spectrophotometry (532 nm)			MDA concentration (nmol MDA/mg protein)	Ohkawa et al., 1979
TRAP Assay	Oxygen Consumption Inhibition: Measures the ability of antioxidants to reduce oxygen consumption, indicating their effectiveness in inhibiting oxidation	Hydrophilic & Lipophilic	HAT	Fluorescence Protection: The molecule fluoresces when protected from oxidation by antioxidants (AOs) in the sample Oxygen Consumption: Indicates the level of oxidation occurring, with AOs preventing it	Total Reactive Antioxidant Potential: Measures the overall capacity of antioxidants to neutralize reactive species Spectrometry/Chemiluminescence Quenching: Techniques used to assess antioxidant activity by measuring light emission reduction	Kinetics (Multiple Points): Data is collected at various time intervals to track changes in a process Lag Time: The initial delay before a response or reaction begins after a stimulus or change	Assesses plasma antioxidant capacity, important for understanding oxidative stress in diseases like multiple sclerosis (MS)	Trolox Equivalents: A measure of antioxidant capacity, expressed in terms of Trolox TAC of Plasma (μmol/L): The overall ability of plasma to neutralize free radicals, quantified in micromoles per liter	Magalhães et al., 2008; Kartau et al., 2024; Siddeeg et al., 2021
TOSC Assay	It measures the ability of cellular antioxidants to neutralize oxyradicals by reducing the oxidation of α-keto-γ-methiolbutyric acid (KMBA). It also evaluates the overall capability of cellular antioxidants to neutralize ROS such as ROO•, HO•, and ONOOH. ROS are artificially generated and react with α-keto-γ-methiolbutyric acid (KMBA), causing its oxidation to ethylene. Antioxidants compete with KMBA to neutralize ROS, thereby reducing ethylene formation	Hydrophilic & Lipophilic	HAT	Ethylene gas formation	Monitored reaction: Production of ethylene due to α-keto-γ-methylthiobutyric acid oxidation. Gas chromatography (GC) with a flame ionization detector (FID) was used to analyze the formation of ethylene in the headspace of vials	Kinetics (multiple points): The time course of ethylene formation is monitored every 12 min	Predicts the oxyradical-mediated adverse effects on the physiological conditions of organisms. Indicates the onset of cellular toxicity, such as lysosomal destabilization, lipid peroxidation, and genotoxic damage. It is less sensitive to pro-oxidant challenges but has greater biological relevance compared to individual antioxidant variations	TOSC values (the ability to inhibit KMBA oxidation), lysosomal membrane stability (Neutral Red retention time), and TOSC of cellular antioxidants, normalized to protein content	Regoli, 2000; Gorbi & Regoli, 2011
Crocin bleaching assay	Measures the inhibition of crocin oxidation by antioxidants, as they compete with crocin for peroxyl radicals, thereby reducing crocin bleaching	Hydrophilic compounds (lipophilic compounds require modifications)	Absorbance of crocin at 440 nm (a reduction in absorbance indicates antioxidant activity)	Absorbance decrease at 443 nm	Bleaching of a hydrophilic carotenoid derivative (strong golden-yellow hue) using UV spectrophotometry	Ratio of initial crocin bleaching rates: Kinetics (slope of absorbance vs. time) or End-Point (absorbance difference after 10 min)	Relevant to biological systems for chain-breaking antioxidant activity, this method evaluates the ability to protect crocin from oxidative damage and measures the antioxidant capacity of plasma, natural compounds, and plant extracts	Inhibition percentage, relative rate constants (Krel), or percent inhibition of crocin bleaching (%Inh)	Magalhães et al., 2008; Bathaie et al., 2011
TEAC Assay	Inhibition of absorbance by antioxidants of the radical cation ABTS• + (2,2′-azinobis(3-ethylbenzothiazoline 6-sulfonate)	Hydrophilic & Lipophilic	SET	Chromogen radical: 2,2′-azinobis(3-ethylbenzothiazoline-6-sulfonic acid) (ABTS^2^⁻) forms ABTS•⁺ (a blue-green chromophore with maximal absorption at 734 nm)	Spectrophotometric measurement of discolouration of ABTS• +	Kinetics or End-point	Limited biological relevance; does not assess all types of antioxidants but measures antioxidant capacity in biological fluids	Relative antioxidant ability of samples, calibrated with Trolox as a standard	Aruoma, 2003; Silvestrini & Mancini., 2024
TRAA (Tocopheroxyl Radical Attenuating Ability)	Measures the ability of antioxidants to attenuate α-tocopheroxyl radicals (α-TO•) in micelles	Hydrophilic & Lipophilic	HAT	α-Tocopheroxyl radical (α-TO•) attenuation	Electron Spin Resonance (ESR) Spectroscopy	Kinetics	Predicts the ability of compounds to inhibit LDL lipid peroxidation, relevant to atherosclerosis research	α-TO• decay rate, anti-TMP index	Aruoma, 2003; Witting et al., 1996
TOSC (Total Oxidant Scavenging Capacity)	Measures the inhibition of ethylene formation from KMBA oxidation by antioxidants	Hydrophilic & Lipophilic		Ethylene formation	Gas chromatography	Area under the curve (AUC)	Measures antioxidant capacity in biological samples	Relative AUC	Aruoma, 2003;Magalhães et al., 2008
FC assay	Measures total phenolic content (TPC) and reduction capacity by reducing Mo⁶⁺ to Mo^5^⁺ through a redox reaction involving electron transfer from antioxidants to the reagent	Hydrophilic and Lipophilic	SET	Reduction of phosphotungstic/phosphomolybdic acid to form a blue chromophore	Measures total phenol content (TPC)Colorimetry (color formation) at 750 nm	End-Point	Measures total phenolic content, which correlates with antioxidant capacity in foods	Total phenolic content (TPC) in GAE (Gallic Acid Equivalents)	Cano et al., 1998; Rumpf et al., 2023; Rumpf et al., 2023; Mehdi & Rizvi, 2013; Magalhães et al., 2008; Pérez et al., 2023;Siddeeg et al., 2021
DMPD (N,N-dimethyl-p-phenylene diamine dihydrochloride) method	DMPD is oxidized by plasma oxidants to produce a stable pink color (DMPD + radicals). The intensity of the color is proportional to the oxidative capacity of the plasma	Hydrophilic & Lipophilic	HAT	DMPD (N,N-Dimethyl-p-phenylenediamine dihydrochloride)	Spectrophotometric measurement at 505 nm	End-point measurement (color stability for at least 30 min)	Measures plasma oxidative capacity, which increases with human aging, reflecting oxidative stress	Plasma oxidative potential (ferric iron equivalents)	Mehdi & Rizvi, 2013; Siddeeg et al., 2021
H_2_O_2_ Scavenging Activity Assay	Uses horseradish peroxidase to oxidize scopoletin into a nonfluorescent product	Lipophilic	HAT	Absorbance at 230 nm (H₂O₂ depletion) or 504 nm (quinoneimine chromogen)	UV–Vis spectrophotometry	End-point (absorbance measured after 10 min)	H₂O₂ is a ROS involved in oxidative stress, lipid peroxidation, and DNA damage	Percentage of H₂O₂ scavenged	Chelliah et al., 2022; Haida & Hakiman, 2019
Conjugated Diene Assay	Detection and quantification of hydroperoxides containing conjugated diene structures formed during lipid oxidation	Lipophilic	HAT	Absorbance at 233 nm	UV spectroscopy	End-point measurement	Relevant to dietary lipids, oil-in-water emulsions, and biological matrices stabilized by proteins	Molar concentration of conjugated diene hydroperoxides	Ribourg-Birault et al., 2024
Metal Chelating Assay	Chelating Ferrous Ions (Fe^2^⁺): Antioxidants prevent the Fenton reaction by chelating Fe^2^⁺ Fenton Reaction: Generates highly reactive hydroxyl radicals from hydrogen peroxide and Fe^2^⁺ Chelating Agents: Stabilize metal ions (e.g., Fe^3^⁺), preventing participation in radical reactions Act as secondary antioxidants by inhibiting metal-catalyzed oxidation Antioxidant Capacity Indicator: The ability to chelate Fe^2^⁺ is a key measure of antioxidant activity	Hydrophilic & Lipophilic	Redox reaction (Neither HAT/SET)	Absorbance of Fe^2^⁺-ferrozine complex at 562 nm	Spectrophotometry Spectrophotometry (562 nm with ferrozine)	End-point	Reduces oxidative stress by inhibiting the Fenton reaction, thereby protecting biomolecules from damage caused by reactive radicals	Percentage Chelating Effect: Measures the extent to which a substance binds and neutralizes metal ions IC50 Value: Concentration at which 50% inhibition of a specific reaction or activity occurs Binding Affinity: The strength of the interaction between a chelating agent and a metal ion % Chelation of Metal Ions: Percentage of metal ions effectively chelated or bound by the substance	Wong et al., 2014; Gulcin & Alwasel, 2022; Kotha et al., 2022; Chelliah & Oh, 2022; Danet, 2021a, b; Moghrovyan et al., 2019; Dinis et al., 1994

### Electron spin resonance (ESR) assays

Another technique, electron spin resonance (ESR) spectroscopy, directly measures nitric oxide radical scavenging (Huang et al., 2005). Additionally, the Griess reaction is commonly used, involving a two-step diazotization process in which nitrous acid, derived from nitrite, reacts with sulfanilic acid to form a diazonium ion. This ion subsequently couples with N-(1-naphthyl) ethylenediamine, producing a chromophoric azo compound, whose intensity is measured spectrophotometrically at 548 nm (Prior et al., 2005; Vamanu, 2012).

The ESR assay, also known as electron paramagnetic resonance (EPR), is a powerful technique for directly detecting and quantifying free radicals in biological and chemical systems. It utilizes spin traps, which are compounds that react with short-lived free radicals to form stable, detectable spin adducts. This highly sensitive method can be used both in vitro and in vivo to study oxidative stress and antioxidant activity (Halliwell & Gutteridge, 2015; Shahidi & Zhong, 2020; Valko et al., 2007).

Basic ESR Signal Generation:

$$\mathrm{Radical}\;(\mathrm R^\bullet)\;+\;\mathrm{Spin}\;\mathrm{Trap}\;(\mathrm{ST})\;\rightarrow\;\mathrm{Radical}\;\mathrm{Adduct}\;(\mathrm{ST}-\mathrm R^\bullet)$$

The principle behind ESR is that free radicals are inherently unstable and short-lived, making direct detection challenging. Spin traps such as 5,5-dimethyl-1-pyrroline *N*-oxide (DMPO) or phenyl-*N*-tert-butylnitrone (PBN) react with free radicals to form stable adducts, which can then be measured using ESR spectroscopy. The resulting ESR spectra provide information about the type and concentration of free radical present (Dikalov & Harrison, 2014). ESR is widely used to study ROS/RNS, and other free radicals in biological systems. It is particularly valuable for evaluating the radical-scavenging activity of antioxidants and understanding their mechanisms of action (López-Alarcón & Denicola, 2013).

ESR spectroscopy is often used to characterize humic substances (HSs), as it is one of the few methods that can provide structural information. ESR spectra of HSs strongly depend on their origin. EPR analysis has been used to examine the antioxidant activity of HSs, revealing that a higher content of phenolic OH groups and paramagnetic centers enhances their antioxidant activity (Csicsor et al., 2023).

Antioxidant assays are essential tools for assessing the ability of compounds to neutralize free radicals or prevent oxidative damage. Different assays target specific aspects of oxidative processes, such as lipid peroxidation, radical scavenging, or metal chelation (Kumari et al., 2025). No single assay can comprehensively evaluate antioxidant activity due to the complexity of oxidative processes and the diverse mechanisms of antioxidants. Therefore, a combination of assays is recommended for a holistic assessment (Zhong and Shahidi, 2015). Assays vary in terms of complexity, required reagents, specificity, and sensitivity, making it crucial to select the appropriate method based on research objectives and sample type (Apak et al., 2016).

### Chemiluminescence vs. fluorescence methods for antioxidant activity assays

Luminescence methods, including chemiluminescence (CL) and fluorescence, are widely used for assessing antioxidant activity due to their high sensitivity, simplicity, and versatility. Both rely on the interaction of antioxidants with ROS/RNS, but differ in principles, applications, and limitations. Chemiluminescence is based on light emission from chemical reactions producing electronically excited states that decay by photon release (Danet, 2021a, b). Common CL reagents such as luminol, lucigenin, and pholasin react with ROS/RNS to emit light, which antioxidants inhibit by scavenging these species, with the degree of inhibition correlating to antioxidant capacity (Christodouleas et al., 2012). In contrast, fluorescence methods measure the quenching of fluorescence intensity caused by antioxidants interacting with fluorophores like fluorescein or Nile blue, which are excited by light and monitored during fluorescence decay (Godoy-Navajas et al., 2011).

CL methods offer high sensitivity owing to low background noise and the absence of an external light source, minimizing interference and enabling trace analysis of antioxidants at low concentrations (Pamunuwa & Atapattu, 2023). Fluorescence methods, while also sensitive, may be affected by sample turbidity or color that interfere with light absorption and emission, but often provide better selectivity for specific antioxidants due to tunable fluorophore properties (Krzymiński et al., 2019). Both approaches find extensive use in food analysis, pharmaceuticals, and biological systems: CL is effective for measuring TAC in complex matrices such as edible oils, wines, and plant extracts (Danet, 2021a, b), whereas fluorescence assays like the ORAC are popular for beverages and functional foods (Godoy-Navajas et al., 2011). Furthermore, CL has been adapted for high-throughput screening and microfluidic platforms, offering rapid and cost-effective antioxidant analysis (Sun et al., 2019a, b).

Despite their advantages, CL methods are limited by relatively low quantum efficiency and structural constraints favoring easily oxidized compounds (Krzymiński et al., 2019), while fluorescence techniques require more complex instrumentation and are susceptible to interference from sample matrices (Barba et al., 2013). Both methods hold biological relevance by quantifying the radical scavenging capacity of antioxidants against ROS/RNS. CL is particularly useful for imaging antioxidant distribution in tissues and evaluating oxidative stress in diseases such as rheumatoid arthritis and Parkinson’s disease (Hughes et al., 2018), while fluorescence provides superior spatial and temporal resolution, making it preferred for cellular imaging applications (Prolo et al., 2018). Together, chemiluminescence and fluorescence serve as complementary tools in antioxidant assays: CL excels in sensitivity and cost-effectiveness for high-throughput and trace analyses, while fluorescence offers greater selectivity and real-time monitoring. The choice between these methods depends on the specific application, sample type, and sensitivity requirements.

## Non -enzymatic antioxidant assays

### Common ROS-based antioxidant assays

#### ORAC assay

The ORAC assay remains a widely recognized and extensively used method for evaluating antioxidant capacity in various substances, particularly in food and biological samples. This assay measures the ability of antioxidants to scavenge ROO•, a type of ROS involved in oxidative damage within biological systems (Huang et al., 2005; Wakagi et al., 2016). The ORAC method operates based on the hydrogen atom transfer (HAT) mechanism. Antioxidants compete with a fluorescent probe, typically fluorescein, for ROO• generated by thermal decomposition of azo compounds such as 2,2'-azobis(2-amidinopropane) dihydrochloride (AAPH) (Dávalos et al., 2003; Lucas-Abellán et al., 2008). As these radicals oxidize fluorescein, its fluorescence diminishes over time. The assay quantifies how effectively antioxidants delay this fluorescence decay. Antioxidant capacity is then expressed as Trolox equivalents by calculating the area under the fluorescence decay curve (AUC) (Albano et al., 2015; Cao et al., 1993). This approach allows simultaneous quantification of both lipophilic and hydrophilic antioxidants, enhancing the assay’s versatility for diverse sample types (Chandrasekara & Shahidi, 2011; Magalhães et al., 2008).

Unlike single electron transfer (SET)-based assays such as DPPH, the ORAC assay captures both the reactivity and stoichiometry of antioxidants. This feature provides a more comprehensive representation of antioxidant behavior in biological systems (Skrede et al., 2004; Wu et al., 2017). Consequently, ORAC is highly valued in food science for high-throughput antioxidant screening and has been applied to numerous matrices including milk peptides, plant proteins, seafood, and fruit and vegetable extracts (Amigo-Benavent et al., 2021; Chung et al., 2010; Moyo et al., 2018). Many studies have leveraged the ORAC assay to explore antioxidant potential. For example, various cabbage cultivars display different ORAC values, correlating with their phytochemical profiles (Mabuchi et al., 2019). Hawthorn fruit extracts also show significant variability in antioxidant capacity as measured by ORAC (Wu et al., 2017). In marine research, the assay has been used to assess antioxidants from the sponge *Cliona celata*, illustrating its applicability across diverse biological systems (Alves et al., 2021; Kinnunen et al., 2005).

Beyond food science, ORAC plays a crucial role in clinical and nutritional research. It is employed to assess dietary antioxidant intake and its relationship to health outcomes, particularly those linked to oxidative stress and metabolic diseases (Mirzababaei et al., 2018; Prior et al., 2003). Its ability to distinguish hydrophilic and lipophilic antioxidants increases its relevance for investigating diet-disease interactions (Chandrasekara et al., 2012; Chen et al., 2013). The ORAC assay has also been adapted for electrochemical applications. Techniques such as cyclic voltammetry monitor antioxidant oxidation by tracking changes in anodic peak areas. This adaptation has been used for lipid-soluble antioxidants like tocopherol and ethoxyquin, as well as for radical scavenging kinetics using AIBN (Haque et al., 2021).

Despite its advantages, the ORAC assay has some limitations. Fluorescence-based measurements may be affected by interfering substances in complex food matrices, which can reduce accuracy (Barba et al., 2013). Furthermore, reliance on specific fluorophores like fluorescein restricts assay conditions. Nevertheless, ORAC generally offers greater sensitivity and higher antioxidant capacity values than other assays such as TEAC, DPPH, and PCL. It also correlates strongly with key bioactive compounds like ascorbic acid and phenolics, especially in plant-based beverages (Barba et al., 2013; Skrede et al., 2004; Zhong et al., 2012). Overall, the ORAC assay remains a highly sensitive, accurate, and versatile method for evaluating antioxidant activity, particularly against peroxyl radicals. Its ability to measure both kinetic and stoichiometric antioxidant properties, along with its adaptability to various food, clinical, and environmental samples, highlights its enduring importance in antioxidant research (Chandrasekara et al., 2012).

### Superoxide radical anion scavenging assay

The superoxide anion (O₂^•⁻^) scavenging activity assay is a widely used method to evaluate the ability of antioxidants to neutralize superoxide anions, a highly ROS implicated in oxidative stress and cellular damage (Carocho & Ferreira, 2013). This assay is optimized for enzymatic antioxidants and measures the competition kinetics of O₂^•⁻^ reduction between cytochrome C (a probe) and the test sample (Xie et al., 2016a, b). The scavenging capacity of antioxidants is quantified by measuring absorbance at 320 nm using a spectrophotometer, with ascorbic acid often used as a positive control and deionized water as the blank (Lalhminghlui & Jagetia, 2018).

The assay can also employ the pyrogallol autoxidation method, where pyrogallol undergoes autoxidation in an alkaline environment, generating superoxide anions (O₂^•⁻^) (Zeng et al., 2020a, b). Additionally, the PMS/NADH (phenazine methosulfate system or the xanthine/xanthine oxidase system)-NBT system is used to generate superoxide anions from dissolved oxygen, which then reduce nitroblue tetrazolium (NBT). The decrease in absorbance at 560 nm indicates the consumption of superoxide anions by antioxidants, with the percentage inhibition calculated to determine scavenging activity (Yang et al., 2008a, b).

**Table:** 

NADH/PMS System (Non-Enzymatic) Superoxide Generation: NADH + H^+^ + PMS → NAD^+^ + PMSH_2_ ​PMSH_2_ + O_2_ → PMS + O_2_^•−^ + 2H ^+^ NBT Reduction (Detection of O_2_^•−^): O_2_^•−^ + NBT → Formazan (Blue) + O_2_ Antioxidant Scavenging (If Present): O_2_^•−^ + Antioxidant (AH) → H_2_O_2_ + A^•^ *(The antioxidant donates a hydrogen atom to neutralize* O₂^•⁻^*)*	Xanthine/Xanthine Oxidase System (Enzymatic) Superoxide Generation: Xanthine + O_2_ + H_2_O → Xanthine Oxidase Uric Acid + O_2_^•−^ + H^+^ Cytochrome c Reduction (Detection of O₂⁻): O_2_^•−^ + Cytochrome c (Fe^3+^) → O_2_ + Cytochrome c (Fe^**2+**^) *(The reduction of Cytochrome c is monitored at 550 nm.)* Antioxidant Scavenging (If Present): O_2_^•−^ + Antioxidant (AH) → H_2_O_2_ + A^•^

Polysaccharides with electrophilic groups, such as keto or aldehyde, facilitate hydrogen release, enhancing their ability to scavenge superoxide anions (Rumpf et al., 2023). Phenolic compounds, including flavonoids and catechins, are particularly effective at scavenging superoxide anions, with their efficiency influenced by the concentration of phenol and the number/position of hydroxyl groups (Carocho & Ferreira, 2013).

This assay is widely applied to measure the superoxide-neutralizing ability of various antioxidants, such as flavonoids, catechins, and BHT (butylated hydroxytoluene), and to study their role in preventing oxidative stress (Xie et al., 2016a, b). It is also used to compare the effectiveness of different antioxidants in protecting biological systems from oxidative damage (Yang et al., 2008a, b). For example, studies have shown that rutin exhibits lower scavenging activity compared to BHT, with both compounds demonstrating concentration-dependent scavenging of superoxide radicals, as indicated by their IC50 values (0.13 mg/mL for rutin and 0.16 mg/mL for BHT) (Yang et al., 2008a, b).Overall, the superoxide anion scavenging activity assay is a valuable tool for assessing the antioxidant potential of compounds, providing insights into their ability to mitigate oxidative stress and protect biological systems from ROS-induced damage(Carocho & Ferreira, 2013).

### Hydroxyl radical scavenging assay

The hydroxyl radical scavenging assay is a critical method for evaluating the ability of antioxidants to neutralize hydroxyl radicals (HO^•^), which are highly reactive and can cause severe cellular damage (Rumpf et al., 2023). Hydroxyl radicals are generated through the Fenton reaction, which involves the interaction of Fe^2^⁺ (ferrous ions) with hydrogen peroxide (H₂O₂) (Carocho & Ferreira, 2013). The scavenging activity of antioxidants, such as polysaccharides, is investigated by their ability to suppress HO• generation or clear formed radicals, particularly through electron-donating groups and iron-chelating mechanisms (Rumpf et al., 2023).

In this assay, hydroxyl radicals are indirectly detected through the hydroxylation of* p*-hydroxybenzoic acid or by monitoring the decay of fluorescein (FL), a fluorescent probe (Carocho & Ferreira, 2013). The reaction mixture typically includes FeSO₄, salicylic acid–ethanol solution, and hydrogen peroxide, incubated for 60 min at 25 °C. The presence of hydroxyl radicals is measured spectrophotometrically at 510 nm or by monitoring fluorescence decay in the presence and absence of antioxidants (Carocho & Ferreira, 2013). Antioxidant activity is quantified by calculating the net area under the curve (AUC), which is the difference between the sample AUC and the blank AUC (Xie et al., 2016a, b). Ascorbic acid is commonly used as a positive control, while deionized water serves as the blank sample (Fan et al., 2024).

Electrochemical hydroxyl radical (^•^OH) assays based on Fenton chemistry use gold electrodes coated with self-assembled thiol monolayers (SAM). The ^•^OH radicals generated from H₂O₂/Fe^2^⁺ degrade the SAM layer, exposing the electrode surface and increasing redox probe signals. Antioxidants compete with SAM for ^•^OH, reducing signal generation proportionally to their scavenging capacity. This method has quantified antioxidant activity in plant extracts (lemongrass, chamomile, etc.) and flavonoids (myricetin, catechin). The approach provides an indirect but sensitive measurement of •OH scavenging through electrochemical signal modulation (Haque et al., 2021).

This assay is particularly useful for evaluating the antioxidant potential of various compounds, such as polysaccharides, and their effectiveness in protecting biological systems from oxidative stress (Rumpf et al., 2023). It provides valuable insights into the mechanisms by which antioxidants neutralize hydroxyl radicals, making it a critical tool for assessing the protective capacity of natural and synthetic antioxidants in food, pharmaceutical, and biomedical applications (Carocho & Ferreira, 2013; Chibuye et al., 2024).

### H₂O₂ (hydrogen peroxide) scavenging activity assay

The hydrogen peroxide (H₂O₂) scavenging activity assay is a widely used method for evaluating the antioxidant capacity of various compounds and extracts. This assay is particularly important because hydrogen peroxide is a ROS that can cause oxidative damage to cells, contributing to the development of diseases such as cancer and neurodegenerative disorders (Fidrianny et al., 2016; Routh et al., 2022). The ability of antioxidants to neutralize H₂O₂ is critical for maintaining cellular health and preventing oxidative stress.

The H₂O₂ scavenging assay typically measures the decrease in absorbance of a hydrogen peroxide solution after the addition of a test sample. The principle of the assay is based on the reaction between hydrogen peroxide and antioxidants, which donate electrons to neutralize H₂O₂, converting it into water (Kalaisezhiyen et al., 2017). This reaction is quantified spectrophotometrically, with results expressed as the percentage of H₂O₂ scavenged relative to a control (Fidrianny et al., 2016; Maltaş & Yıldız, 2011).

Research has shown that various plant extracts exhibit significant H₂O₂ scavenging activity. For example, extracts from medicinal plants have demonstrated effective H₂O₂ scavenging, with their capacity often correlating with their total phenolic and flavonoid content (Kalebar et al., 2020; Saha et al., 2017). These compounds are known for their antioxidant properties, which play a key role in mitigating oxidative stress (Kalaisezhiyen et al., 2017; Saha et al., 2017). However, the effectiveness of different extracts can vary significantly, underscoring the importance of selecting appropriate sources for antioxidant studies (Ghaffar et al., 2021; Saha et al., 2017).

The methodology for the H₂O₂ scavenging assay can vary but generally involves preparing a solution of H₂O₂ at a specific concentration, mixing it with the test sample, and measuring the absorbance at a defined wavelength (often around 230 nm) after a set incubation period (Kang et al., 2017; Maltaş & Yıldız, 2011). This approach provides valuable insights into the antioxidant potential of tested compounds, which is useful for both pharmacological applications and food preservation strategies (Fidrianny et al., 2016; Jusri et al., 2019; Routh et al., 2022).

Another related method involves measuring the inhibition of scopoletin oxidation by horseradish peroxidase. In this assay, horseradish peroxidase oxidizes scopoletin into a non-fluorescent product, and antioxidants inhibit this reaction (Carocho & Ferreira, 2013). However, results from this method can sometimes be ambiguous due to the involvement of multiple inhibitory pathways, making it less straightforward than the H₂O₂ scavenging assay.

### Common RNS-based antioxidant assays

#### Nitric oxide radical scavenging capacity assay

The nitric oxide radical (ON^**.**^) scavenging capacity assay is a critical method for evaluating the antioxidant potential of various compounds, particularly in the context of oxidative stress and related pathologies. This assay measures the ability of antioxidants to neutralize nitric oxide radicals, which are known to play a significant role in various physiological and pathological processes, including inflammation and neurodegeneration (Kumar et al., 2019; Lalhminghlui & Jagetia, 2018). The assay typically involves the generation of nitric oxide from sodium nitroprusside in an aqueous solution, where the interaction of nitric oxide with oxygen leads to the formation of nitrite ions, which can be quantified using the Griess reagent (Prior et al., 2005; Vamanu, 2012).

The mechanism by which antioxidants scavenge nitric oxide involves competition with oxygen, thereby reducing the formation of nitrite ions. This competition is crucial because elevated levels of nitric oxide can react with superoxide radicals to form peroxynitrite, a highly reactive species that contributes to cellular damage (Huang et al., 2005; Savitha et al., 2018). Various studies have demonstrated that plant extracts and bioactive compounds exhibit significant nitric oxide scavenging activities, often in a dose-dependent manner. For instance, extracts from *Cardiospermum halicacabum* have been shown to effectively scavenge nitric oxide radicals, indicating their potential therapeutic applications (Savitha et al., 2018; Zhang et al., 2020). Similarly, the ethanolic extracts of *Eclipta alba* have demonstrated a capacity to inhibit nitrite generation, further supporting their antioxidant efficacy (Baldi, 2013; Ramesh et al., 2011).

Moreover, the antioxidant activity of flavonoids and other phenolic compounds has been extensively studied, revealing their ability to scavenge nitric oxide through various mechanisms, including hydrogen atom transfer and electron donation (Kumar et al., 2019; Zhang et al., 2020). The antioxidant potential of these compounds is often assessed alongside other assays, such as the DPPH assay, to provide a comprehensive evaluation of their radical scavenging capabilities (Prior et al., 2005; Ramesh et al., 2011). For example, studies on *Withania somnifera* roots have highlighted their significant nitric oxide scavenging activity, which correlates with their total phenolic content (Huang et al., 2005; Paul et al., 2016).

The ability of antioxidants, such as sulfur-containing compounds, to scavenge nitric oxide radicals is measured using various techniques. In aqueous solutions, nitric oxide radicals are generated, and their scavenging activity is quantified. One method involves an amperometric sensor, which utilizes an electrochemical approach to detect nitric oxide radicals (Kumar et al., 2019).

### Peroxynitrite (ONOO⁻) scavenging assays.

Peroxynitrite (ONOO⁻) is a highly reactive oxidant formed by the rapid reaction between superoxide radical anion and nitric oxide. As a potent RNS, ONOO⁻ can cause substantial oxidative and nitrosative damage to critical biomolecules including proteins, lipids, and nucleic acids, contributing to the pathology of various inflammatory and neurodegenerative diseases (Chen et al., 2016). The assessment of ONOO⁻ scavenging capacity has thus emerged as a vital component of antioxidant evaluation strategies, particularly in the context of drug discovery and nutraceutical development.

Several natural and synthetic compounds have demonstrated promising peroxynitrite-scavenging potential. Bhat and Madyastha (2001) evaluated the scavenging capacity of phycocyanin and its chromophore phycocyanobilin (PCB), derived from *Spirulina platensis*. Using a pyrogallol red bleaching assay, they observed that both compounds could effectively neutralize ONOO⁻, with phycocyanin exhibiting superior activity. Notably, PCB significantly inhibited ONOO⁻-induced single-strand breaks in plasmid DNA in a dose-dependent manner, suggesting potential therapeutic applications in oxidative stress-related conditions.

In a broader screening approach, Choi et al. (2002) employed a fluorometric method to evaluate the ONOO⁻ scavenging activity of 28 herbal extracts. *Hamamelis virginiana* (witch hazel) bark extract demonstrated the strongest activity, attributed primarily to hamamelitannin, a potent polyphenolic compound. Its scavenging ability was comparable to the well-known antioxidant penicillamine, underscoring the relevance of herbal sources in mitigating ONOO⁻-mediated pathologies such as Alzheimer's disease, arthritis, and certain cancers.

The structural features of polyphenols play a key role in their ONOO⁻ scavenging abilities. Könczöl et al. (2010) utilized a pyrogallol red bleaching assay coupled with LC-DAD analysis to screen phenolic compounds in *Salvia* species. They found that compounds with catechol moieties, such as rosmarinic acid and salvianolic acids A and B, were particularly effective scavengers. This approach not only facilitated rapid identification of bioactive antioxidants in complex plant matrices but also provided a robust alternative to more labor-intensive tyrosine nitration assays.

Similarly, Nugroho et al. (2014) examined *Euphorbia supina*, a traditional medicinal herb, for its ONOO⁻ scavenging potential. HPLC analysis revealed high concentrations of ellagic acid and galloylated flavonoid glycosides-isoquercitrin 6″-gallate and astragalin 6″-gallate-all of which exhibited potent activity, especially ellagic acid (IC₅₀ = 0.89 μM). These findings support the traditional use of *E. supina* in treating gastrointestinal disorders linked to oxidative stress.

Advancements in probe-based detection methods have significantly enhanced the sensitivity and specificity of ONOO⁻ quantification. Li et al. (2020) developed a novel anthraquinone-based fluorescent probe (L) capable of detecting ONOO⁻ in living cells with a detection limit as low as 13 nM. The probe exhibited rapid colorimetric and fluorescent responses and was validated through spectroscopic and computational analyses, demonstrating excellent potential for real-time monitoring in biological systems.

Other studies have explored therapeutic agents with incidental antioxidant benefits. Chen et al. (2016) reported that entacapone (ENT), a Parkinson’s disease medication, scavenged ONOO⁻ more effectively than vitamin C in a dihydrorhodamine (DHR 123) assay. ENT neutralized 76% of ONOO⁻ at 20 µM, compared to 46% for vitamin C, highlighting its potential beyond neuroprotection.

In the search for more sensitive detection platforms, Bekdeser et al. (2016) introduced a spectrofluorometric method using gentisic acid as a probe. Due to its high molar fluorescence coefficient, gentisic acid enabled the precise detection of ONOO⁻ at low concentrations. Among tested biothiols and amino acids, cysteamine and cysteine displayed the strongest scavenging activities. Liver tissue homogenates showed the highest ONOO⁻ scavenging capacity, reflecting the organ’s vital role in redox regulation.

Further advancing the detection of ONOO⁻ in live-cell systems, Zhen Luo et al. (2017) developed the DAX-J2 PON Green probe. This fluorescence-based method allowed real-time quantification of ONOO⁻ in various cell lines via flow cytometry and microplate readers. The probe was used to monitor ONOO⁻ levels induced by SIN-1 and evaluate the inhibitory effects of curcumin, making it a valuable tool for antioxidant screening and redox biology studies.

A comprehensive review by (Grzelakowska et al., 2024) emphasized the importance of boronate-based probes for ONOO⁻ detection. These probes react rapidly and selectively with ONOO⁻, generating phenolic and nitrated products suitable for analysis through fluorescence, bioluminescence, and chromatography-based techniques. The authors provided detailed insights into the chemical mechanisms, application protocols, and the comparative advantages of different probe types, underscoring the need for accurate and biologically relevant detection systems.

Nevertherless, Apak et al. (2016) reviewed a wide range of antioxidant assays, highlighting the complexity of standardizing ROS/RNS scavenging methods. Their analysis emphasized the value of using multiple complementary assays-including ONOO⁻ scavenging tests-to obtain a comprehensive picture of antioxidant activity in foods, natural products, and biological systems. The integration of chromatographic and chemometric techniques was advocated as a powerful strategy to unravel antioxidant profiles in complex matrices.

Collectively, these studies underscore the critical role of peroxynitrite-scavenging assays in evaluating antioxidant efficacy. They also highlight the therapeutic promise of both natural and synthetic compounds in counteracting ONOO⁻-induced oxidative damage, thereby contributing to the prevention and management of a wide array of oxidative stress-related diseases.

### Synthetic radical scavenging assays

#### DPPH (2,2-diphenyl-1-picrylhydrazyl) radical scavenging assay

The DPPH assay, originally developed by Blois (1958) and later refined, is one of the most widely used preliminary screening methods for evaluating antioxidant activity due to its simplicity, speed, and reproducibility (Carocho & Ferreira, 2013). It measures free radical scavenging capacity by accepting electrons or hydrogen atom, converting the DPPH radical into a stable diamagnetic molecule (Baliyan et al., 2022).The assay operates on the principle of measuring the reduction of the DPPH radical, a stable radical with a deep purple color that exhibits strong absorption at 515–520 nm(Sridhar & Charles, 2019).When antioxidants are present, they donate hydrogen atom to the DPPH radical, causing a color change from purple to yellow, which is quantified spectrophotometrically at 517 nm (Arsul et al., 2022; Wei & Shibamoto, 2010; Puangbanlang et al., 2019). A blank control is prepared using deionized water, and ascorbic acid is commonly used as a positive control (Xie et al., 2016a, b). Results are expressed as EC50 values, which indicate the antioxidant concentration required to reduce free radicals by 50% (Sridhar & Charles, 2019). This method is favored for its simplicity, rapid execution, and sensitivity, making it suitable for screening both natural and synthetic antioxidants (Upadhyay & Mishra, 2014; Yamauchi et al., 2024). However, variability in results may occur due to the nature of the antioxidants tested, as some compounds may exhibit masking effects (Geng et al., 2018). The decrease in DPPH absorbance is directly proportional to the radical scavenging activity of antioxidants (Carocho & Ferreira, 2013).

The DPPH assay is widely used to evaluate the radical scavenging capacityin the food, pharmaceutical, and cosmetic industries, as well as in environmental toxicology to assess oxidative stress reduction caused by pollutants (Rumpf et al., 2023). It measures radical scavenging activity (RSA) based on single electron transfer (SET) and hydrogen atom transfer (HAT) mechanisms. However, it has a slower reaction time (30 min) compared to the ABTS assay due to steric hindrance at the DPPH radical site, and it exhibits lower sensitivity with a higher coefficient of variation (CV = 5.30%) (Rumpf et al., 2023). Recently, an electrochemical version was introduced by Milardovic et al. (2005) where DPPH• reduction at a glassy carbon electrode was monitored. Antioxidant presence decreases the anodic current, correlating with concentration. This method has been applied to food samples (coffee, tea, wine, juices, olive oil) and pure antioxidants (gallic acid, quercetin, ascorbic acid). However, a key limitation is potential interference from other redox-active compounds, reducing selectivity. Despite this, the electrochemical approach offers promise for rapid antioxidant analysis in food and biomedical applications (Haque et al., 2021).

### ABTS (2,2'-Azino-bis (3-ethylbenzothiazoline-6-sulfonic acid)) radical cation scavenging assay

The ABTS^•⁺^ (2,2′-azino-bis (3-ethylbenzothiazoline-6-sulfonic acid) radical scavenging assay is a widely used method to evaluate antioxidant activity by measuring the ability of antioxidants to scavenge the ABTS radical cation, a strong oxidant (Cano et al., 2023). The ABTS• + radical cation is generated through the one-electron oxidation of ABTS, typically using potassium persulfate, and appears as a bluish-green solution with absorption peaks at 415, 650, 732, and 820 nm, with the highest molar absorptivity at 415 nm (3.4 × 10^4^ M⁻^1^ cm⁻^1^) (Kut et al., 2022a, b; Zhao et al., 2024). Antioxidants convert ABTS• + to its non-radical form, leading to decolorization, which is quantified spectrophotometrically at 734 nm (Rumpf et al., 2023). The assay is highly sensitive, with low detection limits (often less than 0.1 μM) and rapid reaction equilibrium times (less than 1 min) (Zhao et al., 2024).

The ABTS assay is versatile and can measure the antioxidant capacity of both hydrophilic and lipophilic compounds, making it suitable for a wide range of sample types (Kut et al., 2022a, b). It is commonly used to evaluate the antioxidant capacity of biologically active compounds, such as carotenoids and phenolic compounds, and to assess the efficiency of antioxidants in scavenging free radicals, which is essential for mitigating oxidative stress (Carocho & Ferreira, 2013; Xie et al., 2016a, b). The results are often expressed as Trolox equivalents (TE) for standardized comparison or as EC50 values, indicating the antioxidant concentration required to reduce free radicals by 50% (Kut et al., 2022a, b; Sridhar & Charles, 2019).

This method is favored for its simplicity, low cost, and minimal technical requirements, as it does not require expensive reagents or equipment (Kut et al., 2022a, b). It is also highly reproducible, with a coefficient of variation (CV) of 0.25%, and exhibits better linearity (R^2^ = 0.99996) compared to the DPPH assay (Rumpf et al., 2023). Additionally, the ABTS assay is stable over extended periods, making it useful for both laboratory analysis and field-based measurements (Zhao et al., 2024). However, it is important to note that the ABTS assay often produces higher antioxidant capacity readings compared to the DPPH assay, particularly for hydrophilic and highly pigmented antioxidants, highlighting the need to select the appropriate assay based on the specific characteristics of the antioxidants being evaluated (Damgaard et al., 2014; Payne et al., 2013).

The ABTS assay has been adapted for modern use with plate readers. The modified ABTS• + decolorization assay involves preparing a stock solution by oxidizing 7 mM ABTS with 2.45 mM potassium persulfate. The solution is diluted with phosphate-buffered saline (PBS) at pH 7.4 to achieve an absorbance of 1.0 at 734 nm in a 96-well microplate. This modification increases the ABTS^•+^ concentration to 106.7 µM, ensuring higher accuracy in absorbance measurements. The assay is particularly useful for evaluating antioxidant activity in complex biological samples like blood plasma and plant extracts. Electron paramagnetic resonance (EPR) measurements complement the assay, using specific settings to analyze ABTS^•+^ stability and interactions. The assay is applicable across a broad pH range (2.0–7.4), though ionic strength can affect reactivity. A reaction time of 60 min is recommended for antioxidants, blood plasma, or plant extracts. Statistical models like non-linear regression and software tools (e.g., OriginPro, GraphPad Prism) are used to estimate EC50 values (Kut et al., 2022a, b; Sridhar & Charles, 2019).

The ABTS (TEAC) antioxidant assay was electrochemically adapted using cyclic voltammetry (CV) and square wave voltammetry (SWV). In this approach, ABTS oxidation produces characteristic peaks at + 0.230 V (ABTS^•⁺^) and + 0.520 V (ABTS^2^⁺). Upon adding antioxidants, a regenerative redox mechanism occurs, antioxidants reduce ABTS^2^⁺, increasing the oxidation current while decreasing the reduction current. This electrochemical version offers a rapid, simple alternative to spectrophotometric ABTS assays, successfully applied in edible oil analysis (Haque et al., 2021).

This assay is widely applied in various fields, including food science, pharmaceuticals, and environmental analysis, to detect oxidative species and evaluate the electron-donating capacity (EDC) of dissolved organic matter (Zhao et al., 2024). Its faster reaction time (12 min) and higher sensitivity (slope of calibration curve > 5 times higher than DPPH) make it a preferred choice for antioxidant capacity assessment (Rumpf et al., 2023).

### N, N-dimethyl-p-phenylenediamine (DMPD) radical cation assay

The *N, N*-dimethyl-*p*-phenylenediamine (DMPD) radical cation assay is a widely used method for evaluating the antioxidant capacity of various compounds. This assay is based on measuring the reduction of the DMPD radical cation (DMPD^•⁺^) at a fixed time point, simplifying the analysis by eliminating the need to monitor time-dependent color changes, a common limitation of other methods such as the ABTS or DPPH assays (Gülçın, 2008; Topal et al., 2015). One of the key advantages of the DMPD assay is its ability to simultaneously analyze multiple samples, making it highly efficient for high-throughput screening of antioxidant activity (Aksu et al., 2015; Gülçın, 2008).

In this assay, the DMPD compound is oxidized in the presence of ferric ions to form a stable, colored radical cation (DMPD^•⁺^). When antioxidants are introduced, they donate hydrogen atoms to the DMPD^•⁺^, leading to a reduction in the absorbance of the colored solution, which can be quantitatively measured (Sochor et al., 2010). The reaction reaches a steady endpoint, providing a reliable and reproducible measure of antioxidant capacity (Adam et al., 2018; Topal et al., 2015). This method has proven effective in assessing the radical scavenging abilities of various natural extracts and compounds, including those derived from plants (Adam et al., 2018; Ghadage et al., 2017; Lü et al., 2010).

The DMPD•⁺ assay is similar to the ABTS•⁺ assay but utilizes a more stable radical cation. In this method, colorless DMPD is oxidized by ferric chloride to form the colored DMPD•⁺ radical cation. The presence of antioxidants reduces the radical cation back to colorless DMPD, and the decrease in absorbance at 505 nm is measured to determine antioxidant capacity (Fogliano et al., 1999). Initially developed to assess the antioxidant capacity of wines, the assay yielded results comparable to the ABTS^•⁺^ assay. However, due to the water-soluble nature of DMPD, this assay is unsuitable for hydrophobic compounds. It was later adapted for measuring the TAC of human plasma, expressed as hydrogen peroxide equivalents, and automated for kinetic analysis (Verde et al., 2002).

The DMPD assay is also used to measure peroxynitrite (ONOO⁻) inhibition. These assays are essential for evaluating the ability of compounds to neutralize peroxynitrite, a highly reactive oxidant formed from the reaction of nitric oxide (NO) and superoxide (O₂⁻) (Carocho & Ferreira, 2013). Ortho-phenylenediamine has also been used as a probe for peroxynitrite detection, demonstrating its effectiveness in quantifying peroxynitrite levels in biological systems (Li et al., 2004). These assays measure the inhibition of tyrosine nitration or dihydrorhodamine oxidation caused by peroxynitrite, offering insights into the antioxidative potential of test compounds (Carocho & Ferreira, 2013).

Despite its advantages, the DMPD assay has some limitations. For instance, its sensitivity and reproducibility can be compromised by the presence of hydrophobic antioxidants, such as α-tocopherol (Köksal et al., 2009). Additionally, the assay may produce misleading results when applied to products rich in organic acids, particularly citric acid, due to potential interferences (Corral-Aguayo et al., 2008). Nevertheless, the DMPD assay remains a popular choice among researchers due to its simplicity and effectiveness in determining antioxidant activity across a wide range of biological and food samples (Durmaz et al., 2022; Gil et al., 2000).

### Reduction capacity assays

#### FRAP (ferric reducing antioxidant power) assay

The FRAP assay is a single electron transfer-based method widely used for evaluating the antioxidant capacity of various samples, including plant extracts, foods, and biological fluids (Benzie & Strain, 1996; Carocho & Ferreira, 2013). It operates by reducing ferric ions (Fe^3^⁺) to ferrous ions (Fe^2^⁺) under acidic conditions (pH 3.6), resulting in the formation of a blue-colored Fe^2^⁺-ligand complex, typically [Fe (II)(TPTZ)₂]^2^⁺, with an absorbance maximum at 593 nm (Benzie & Strain, 1996; Gan et al., 2010). The intensity of the blue color is directly proportional to the reducing power of antioxidants present in the sample.

Modern adaptations of the FRAP assay have expanded its versatility. For instance, some variants substitute the original TPTZ ligand with potassium ferricyanide, which leads to the formation of Prussian blue upon reaction with excess ferric ions. However, due to Prussian blue’s tendency to precipitate, modifications such as the addition of stabilizers like sodium dodecyl sulfate (SDS) at lower pH (around 1.7) are employed. This adjustment not only prevents precipitation but also enhances the assay’s capacity to detect thiol-based antioxidants, such as cysteine and GSH, which are often undetected in conventional FRAP settings (Berker et al., 2010; Hsieh & Vani, 2021).

To further improve the assay’s applicability, solvent modifications (e.g., water/acetone mixtures) have been introduced to enable the simultaneous measurement of both hydrophilic and lipophilic antioxidants. Despite these advancements, FRAP is inherently limited by its reliance on single-timepoint readings, which may overlook slowly reacting antioxidants (Shahidi & Zhong, 2015). This limitation has prompted the development of electrochemical adaptations, including coulometric titration and chronoamperometry, which offer enhanced sensitivity, reproducibility, and real-time detection capabilities (Brainina et al., 2012; Pastor et al., 2020).

One recent electrochemical variant introduced by Pastor et al. (2020) maintains the core FRAP conditions (acetate buffer, pH 3.6, Fe(III)-TPTZ complex) but utilizes electrochemical detection instead of spectrophotometry. This approach minimizes interference from sample turbidity or inherent coloration and eliminates the need for calibration standards such as Trolox. The method demonstrated a strong correlation (R^2^ = 0.9) with conventional antioxidant assays (DPPH, ABTS, and traditional FRAP), while also aligning with green chemistry principles by excluding toxic metals and stable radicals.

Despite its simplicity, cost-effectiveness, and broad applicability, the FRAP assay primarily measures non-enzymatic antioxidant capacity (NEAC) via ET reactions and does not account for hydrogen atom transfer (HAT) mechanisms or metal-chelating activity (Hirsch et al., 2013; Shahidi & Zhong, 2015). Furthermore, it exhibits poor reactivity with certain antioxidant classes, such as thiols, and may not fully represent the complex interplay of antioxidants in biological systems (Benzie & Devaki, 2017). Nevertheless, the FRAP assay remains a valuable tool for assessing antioxidant-rich diets, monitoring oxidative stress, and supporting quality control in food, pharmaceutical, and clinical research. Its standardized protocol and adaptability to automated systems ensure comparability across studies, while newer adaptations continue to enhance its robustness and analytical performance.

### Ferricyanide/prussian blue method

The study by Berker et al. (2010) introduced an optimized ferricyanide/Prussian blue assay for assessing TAC, utilizing both ferricyanide and ferric ions as oxidants in an acidic medium (pH 1.7), stabilized with sodium dodecyl sulfate (SDS) to prevent Prussian blue precipitation. This modification enhanced reproducibility, linearity (5–50 µM range for most antioxidants), and additivity for synthetic mixtures, while minimizing interference from non-antioxidants such as citric acid and sugars. Notably, the assay effectively detected thiol-containing antioxidants (e.g., cysteine and GSH), which are often overlooked by other iron-based methods. Validation against reference assays like CUPRAC and FRAP confirmed its reliability across various herbal teas and plant extracts, supporting its practical utility for routine analysis in food and biological samples.

Expanding on this methodology, Cumbane et al. (2024) developed the Ferric-PCCR method, which forms a stable Prussian blue/green chromogenic complex under acidic conditions (80–100 mM HCl). This approach addressed limitations in reagent stability and matrix interference seen in traditional assays. The optimized method exhibited enhanced sensitivity (molar absorptivity up to 4.2 × 10^5^ M⁻^1^·cm⁻^1^), high reproducibility (RSD < 5%), and excellent linearity (R^2^ > 0.99). An inverse exponential correlation between molar absorptivity and IC₅₀ was observed, indicating that higher ε values correspond to stronger antioxidant potency. Application to five plant extracts revealed Olax dissitiflora and Gladiolus dalenii as having the highest TAC. The method, though limited by its acidic medium, offers a rapid (< 30 min), cost-effective, and matrix-compatible alternative suitable for use in food, cosmetic, and pharmaceutical industries.

To extend the application of this assay to biological systems, Brainina et al., (2012) developed an electrochemical variant based on chronoamperometry. This method quantifies antioxidant activity by measuring the anodic current at + 0.35 V (vs. Ag/AgCl) generated during the reduction of ferricyanide (K₃[Fe(CN)₆]) to ferrocyanide (K₄[Fe(CN)₆]). Antioxidant activity is expressed by comparing current responses from sample-induced reduction (ΔI_Formed) with standard additions (ΔI_Add). With a low detection limit (2–5 × 10⁻⁶ M), this technique successfully quantified key antioxidants in blood plasma, including uric acid, ascorbic acid, cysteine, and GSH, demonstrating strong potential for use in clinical and physiological contexts.

Complementing these methods, Kanmaz et al. (2020) introduced a novel nanoparticle-based Prussian blue assay (PBNP) for measuring TAC in *Cynara scolymus* L. (globe artichoke). The use of polyvinylpyrrolidone (PVP) stabilized Prussian blue nanoparticles, significantly enhancing sensitivity and colloidal stability. Compared to conventional assays like CUPRAC and FC, the PBNP method demonstrated superior molar absorptivity, lower limits of detection (LOD: 80 nM for trolox), and broader detection capabilities, including weak antioxidants such as methionine. Validation using artichoke leaf extracts confirmed excellent linearity and reproducibility, while STEM imaging revealed uniformly dispersed spherical nanoparticles (10–30 nm). This nanoparticle-enhanced assay offers a sensitive, reliable, and economical approach for antioxidant evaluation in both food and pharmaceutical applications.

### Reducing power assay

The reducing power assay is a widely used method to evaluate the ability of a compound to donate electrons and reduce Fe^3^⁺ (ferric) to Fe^2^⁺ (ferrous), reflecting its antioxidant potential (Yen & Chen, 1995). An increase in absorbance at 700 nm indicates greater reducing power, with higher absorbance values correlating with stronger antioxidant activity (Yen & Chen, 1995). This assay is based on the principle that antioxidants with reducing power can donate electrons to stabilize free radicals, thereby preventing oxidative damage (Oyaizu, 1986; Shahidi & Zhong, 2015).

The assay is commonly performed using standard antioxidants such as rutin, BHT, and vitamin C (Vc) for comparison (Oyaizu, 1986). The reductive potential of test compounds is assessed by mixing them with phosphate buffer (pH 6.6) and potassium ferricyanide, followed by incubation and the addition of trichloroacetic acid and FeCl₃ to measure absorbance at 700 nm (Oyaizu, 1986). The results are often expressed as IC_50_ values, which represent the concentration of the compound required to achieve 50% of its maximum reducing power, determined using linear regression analysis (Xie et al., 2016a, b).

The reducing power assay is a valuable tool for identifying compounds with strong electron-donating abilities and is widely used to evaluate the antioxidant potential of various organic extracts, including food preservatives, natural extracts, drugs, and nutraceutical preparations (Gülçin & Alwasel, 2025). It is particularly useful for screening antioxidants in food science, pharmaceuticals, and cosmetic industries, as it provides a simple and effective measure of electron-donating capacity (Yen & Chen, 1995). The assay’s ability to quantify reducing power makes it a critical method for assessing the efficacy of antioxidants in mitigating oxidative stress and preventing oxidative degradation in various applications (Oyaizu, 1986).

### CUPRAC (cupric ion ieducing antioxidant capacity) assay

The CUPRAC assay is a widely used method for measuring antioxidant activity and capacity in various media. It employs bis(neocuproine) copper (II) chelate (Cu (II)-Nc) as a chromogenic redox reagent, which operates effectively at physiological pH (pH 7). During the assay, polyphenolic antioxidants with reactive Ar-OH groups are oxidized to quinones, while Cu (II)-Nc is reduced to Cu(I)-Nc, forming a colored chelate that can be measured spectrophotometrically at 450 nm. This redox reaction follows the equation:

$$nCu{(Nc)}_2^{2+}\;+\;n-electronreductant(AOX)\rightarrow nCu{(Nc)}_2^+\;+\;n-electronoxidizedproduct\;+\;nH^+$$

The CUPRAC assay has been optimized for various reaction conditions, including reagent concentration, pH, and oxidation time at different temperatures, to enhance its performance. A significant advantage of this method is its relative independence from solvent effects, making it applicable to both hydrophilic and lipophilic antioxidants. (Capanoglu et al., 2021). This versatility allows it to assess TAC in hydrophobic media more effectively than other assays, such as the FRAP assay. The color development after the redox reaction is rapid for antioxidants like ascorbic acid, gallic acid, and quercetin but slower for compounds such as naringin and naringenin. For most antioxidants, the oxidation reactions are completed within 30 min at room temperature (Apak et al., 2016). However, in the case of flavonoid glycosides, acid hydrolysis is required to fully exhibit their antioxidant activity. For slow-reacting antioxidants, elevated temperatures may be necessary to complete the oxidation process. While oxygen exclusion is unnecessary for freshly prepared antioxidant solutions, plant extracts should be purged with nitrogen (N₂) to exclude oxygen and stored in a refrigerator if not analyzed immediately (Apak et al., 2025).

Compared to other electron-transfer-based TAC assays, such as ABTS DPPH, and FRAP, the CUPRAC method offers several advantages. These include its realistic physiological pH, favorable redox potential, reagent stability, simplicity, and ability to assess both lipophilic and hydrophilic antioxidants. Additionally, it provides additive responses and linear calibration curves over a wide range of concentrations, making it a reliable and comprehensive approach for evaluating antioxidant capacity (Apak, 2017).

The CUPRAC assay has been adapted for specific applications, such as the determination of hypoxanthine (Hx), a key indicator of fish spoilage. This adaptation utilizes a Nafion membrane sensor and relies on the enzymatic conversion of Hx to uric acid (UA) and hydrogen peroxide (H₂O₂) by xanthine oxidase (XOD). Both UA and H₂O₂ react with the CUPRAC reagent (Cu(II)-neocuproine) to form a colored chelate measurable at 450 nm. The method demonstrates a linear range of 2.0–32.0 µM for Hx, with a limit of detection (LOD) of 0.79 µM and a limit of quantification (LOQ) of 2.63 µM. The CUPRAC-Nafion sensor is cost-effective, simple, and versatile, making it suitable for real-time monitoring of fish freshness. It can measure both hydrophilic and lipophilic antioxidants, providing additive responses and linear calibration curves over a wide concentration range. The sensor also shows minimal interference from common biological compounds such as Na⁺, K⁺, Mg^2^⁺, Zn^2^⁺, Ca^2^⁺, lactic acid, and D-glucose. Antioxidants like cysteine (CYS), GSH, ascorbic acid (AA), uric acid (UA), and α-tocopherol are effectively removed through a preliminary oxidation and solvent extraction step, ensuring accurate Hx determination (Borahan et al., 2022).

The CUPRAC-Nafion sensor has been validated against HPLC, showing strong correlation (R^2^ = 0.9921 for CUPRAC vs. 0.9841 for HPLC). It provides comparable accuracy in measuring Hx concentrations in fish samples, with recoveries of spiked Hx ranging from 95.0% to 98.8%. This method is suitable for tracking fish spoilage and predicting shelf life, particularly in the early stages of degradation, and can be applied to both fresh and packaged seafood, offering a sensitive and selective tool for food safety and quality control (Borahan et al., 2022).

Despite its advantages, the CUPRAC assay has certain limitations. It cannot measure highly reactive and short-lived ROS due to their high reactivity and short half-life, which may disappear during sample handling. Additionally, fish samples require homogenization and extraction with perchloric acid (HClO₄) before analysis, adding complexity to the procedure. Plant extracts must be purged with nitrogen (N₂) to exclude oxygen and stored in a refrigerator if not analyzed immediately. While the method includes a step to remove interfering antioxidants, the presence of high concentrations of these compounds may still affect the accuracy of Hx determination if not properly eliminated. Furthermore, the assay measures both H₂O₂ and UA, which are produced from Hx by XOD. While this dual measurement is advantageous, it may complicate the interpretation of results in samples with high endogenous UA levels. The method also requires optimization of reaction conditions (pH, enzyme concentration, and reaction time) for each sample type, which may limit its applicability in high-throughput settings (Borahan et al., 2022).

The CUPRAC assay has been further modified to measure TAC in biological fluids like human serum. This modification is particularly useful for evaluating an organism's ability to counteract ROS and oxidative stress-related diseases, such as cardiovascular, liver, and neurodegenerative disorders. The assay can measure both hydrophilic and lipophilic antioxidants and is especially efficient for thiol antioxidants (e.g., GSH), where the FRAP assay is non-responsive. Results from this assay show strong correlations with ABTS methods for lipophilic antioxidants but moderate correlations for hydrophilic ones. A notable benefit of this method is that the results adhere to Beer’s law (additivity of absorbance). Unlike the Randox-TEAC assay, this method is not sensitive to serum dilution. It also exhibits rapid reaction kinetics, except for compounds like bilirubin and uric acid (Apak, 2019).

Another modification involves the simultaneous measurement of hydrophilic and lipophilic antioxidants using 2% methyl-β-cyclodextrin (M-β-CD) in a water–acetone solvent for TAC determination. This approach objectively measures antioxidants, irrespective of their lipophilicity, and has been validated for linearity, precision, and recovery against ABTS/HRP assays. A related modification allows for ascorbic acid determination in the presence of flavonoids by separating interfering flavonoids via La(III) chelates extractable into ethyl acetate, facilitating accurate measurement in mixtures (Apak et al., 2025).

The hydroxyl radical scavenging of water-soluble antioxidants is another significant modification, which detects hydroxyl radicals using probes like *p*-aminobenzoate. This method offers superior specificity, yield, and convenience compared to thiobarbituric acid reactive substances (TBARS) and identifies effective scavengers such as iodide and hexacyanoferrate(II). A related modification, hydroxyl radical scavenging of phenolics and flavonoids, uses a salicylate probe for detection. It efficiently halts the Fenton reaction within 10 min using CAT and measures the hydroxyl radical scavenging rate constants of polyphenolics (Apak, 2019).

The xanthine oxidase inhibition assay, another CUPRAC assay modification, measures xanthine oxidase inhibitory activity of polyphenols and antioxidants using a modified CUPRAC assay at 450 nm, avoiding interference from UV-absorbing substances. This approach is practical, cost-effective, and reliable for real sample analysis (Wu et al., 2020).

An electrochemical adaptation by Cárdenas et al. (2014) uses cyclic voltammetry in pH 7 ammonium acetate buffer to monitor the redox reaction between [Cu(Nc)₂]^2^⁺ and [Cu(Nc)₂]⁺. Antioxidants generate [Cu(Nc)₂]⁺, producing peak currents proportional to their concentration. This method offers advantages over traditional assays (e.g., FC, DPPH, FRAP) by working under physiological pH, avoiding sugar interference, and accommodating both hydrophilic and lipophilic antioxidants. Its fast reaction kinetics, reagent stability, and selectivity make it suitable for analyzing complex samples like tea.

The CUPRAC assay and its modifications offer a reliable, cost-effective, and versatile approach for determining antioxidant capacity and specific analytes like Hx in various samples. However, its limitations, such as the inability to measure highly reactive ROS and the need for sample preparation, highlight the need for further refinement to enhance its applicability in clinical and industrial settings. Future research could focus on immobilizing both XOD and the CUPRAC reagent on a single membrane for simplified smartphone-based detection, further improving its practicality and accessibility (Apak et al., 2025; Borahan et al., 2022).

### Ceric reducing/antioxidant capacity (CRAC) assay

Ozyurt et al. (2011) developed a novel spectrofluorometric method to determine TAC using the cerium(IV)-reducing antioxidant capacity (CERAC) assay. This technique relies on the selective oxidation of antioxidants by Ce(IV) in an optimized acidic sulfate medium (0.3 M H₂SO₄ + 0.7 M Na₂SO₄), followed by fluorometric detection of the resulting Ce(III) (λₑₓ = 256 nm, λₑₘ = 360 nm). Compared to conventional spectrophotometric methods, the CERAC assay demonstrates superior selectivity by minimizing interference from non-antioxidant substances such as citric acid and simple sugars. The method exhibits a broad linear range (e.g., 0.05–1.0 μM for quercetin), excellent reproducibility (RSD < 5.2%), and additive behavior in complex mixtures. Validation against standard methods (e.g., CUPRAC, ABTS) confirmed its accuracy for evaluating the TAC of flavonoids, phenolic acids, and thiol compounds. When applied to herbal teas, the method ranked antioxidant capacity as: green tea > sage > nettle > mint > linden > chamomile. The assay’s selectivity, simplicity, and elimination of organic solvents make it suitable for routine food analysis.

In a related study, Ozyurt et al. (2010) introduced a modified CERAC assay that refines redox potential conditions in the same sulfate-based medium. This method effectively hydrolyzes and oxidizes flavonoid glycosides (e.g., naringin to naringenin) while avoiding interference from sugars and acids. It also correlates strongly with other TAC assays such as ABTS and CUPRAC, making it a practical, low-cost option for use in conventional food laboratories.

Geng et al. (2018) investigated how interactions between bovine serum albumin (BSA) and dietary flavonoids affect antioxidant activity. Using a combination of electrochemical techniques (cyclic voltammetry and CERAC assays) and spectrophotometric methods (DPPH and FRAP), they found that BSA binding reduced the oxidation peaks of flavonoids in voltammetry, suggesting a “masking effect” that diminishes their electron-donating ability. The CERAC assay consistently showed significant reductions (67–82%) in antioxidant activity for bound flavonoids like morin, galangin, and catechin. In contrast, DPPH and FRAP produced inconsistent results, likely due to solvent interactions. The CRAC method, measuring Ce^4^⁺ consumption via chronoamperometry, proved more reliable for assessing antioxidant activity in protein-rich environments, better reflecting physiological conditions.

The ceric reducing antioxidant capacity (CRAC) assay, as described by Haque et al. (2021), offers a direct electrochemical approach to quantify antioxidant activity by measuring the reduction of Ce^4^⁺ to Ce^3^⁺ using chronoamperometry at + 0.8 V (vs. Ag/AgCl) on a boron-doped diamond electrode in 0.5 M H₂SO₄. This method accurately ranked antioxidants in the order: tannic acid ≈ quercetin > rutin > gallic acid ≈ catechin > ascorbic acid > BHA > Trolox, in alignment with FRAP results. Applied to fruit juices and plant extracts, the CRAC assay demonstrated reduced matrix interference due to its lower reduction potential, making it a reliable alternative for evaluating antioxidant capacity.

#### TAC assays

There is a growing preference for assessing the TAC of samples rather than individual antioxidants. Popular methods include ABTS, CUPRAC, FRAP, DPPH, and ORAC. These methods account for the synergistic effects of multiple antioxidants in complex biological matrices, providing a more comprehensive assessment of antioxidant capacity (Apak, 2019).

### Phosphomolybdenum assay

The phosphomolybdenum assay is a widely utilized and reliable method for evaluating the TAC of a variety of substances, particularly plant extracts, food samples, and phytochemicals. This assay is based on the reduction of molybdenum (VI) to molybdenum (V) by antioxidant compounds in an acidic medium, resulting in the formation of a green-colored phosphomolybdenum complex that can be quantified spectrophotometrically at 695 nm (Fidrianny et al., 2016; Özkan et al., 2007; Prieto et al., 1999). The procedure typically involves mixing the sample with a reagent solution composed of sulfuric acid, sodium phosphate, and ammonium molybdate, followed by incubation at 95 °C for 90 min, during which the antioxidant compounds promote the reduction reaction (Cardeñosa et al., 2002; Chandran et al., 2011). Tocopherol isomers, such as α-, γ-, and δ-tocopherol, are commonly used as standards for lipid-soluble antioxidants, while ascorbic acid is used for water-soluble equivalents. The assay provides a comprehensive measure of the overall antioxidant potential of compounds, which is essential for understanding their capacity to mitigate oxidative stress and related cellular damage (Bi et al., 2011; Kasangana et al., 2015). Moreover, the phosphomolybdenum assay has shown strong correlation with other antioxidant assessment methods, including DPPH and ABTS assays, thereby reinforcing its robustness and accuracy (Baskar et al., 2020; Souza et al., 2021). Its application spans numerous research contexts-from evaluating medicinal plant extracts like *Leea macrophylla* and *Origanum sipyleum* to analyzing the antioxidant properties of various bioactive molecules such as flavonoids and phenolics known for their free radical scavenging effects (Albayrak & Aksoy, 2012; Dalarmi et al., 2017; Durgadas & Acharya, 2021; Mahdjar et al., 2019). Due to its simplicity, reproducibility, and broad applicability, the phosphomolybdenum assay remains a valuable tool in both academic and industrial settings focused on identifying and developing antioxidant-rich natural or synthetic products.

### TRAP (total radical-trapping antioxidant parameter) assay

The TRAP assay, originally developed by Wayner et al. (1985), is a comprehensive and sensitive method used to evaluate the TAC of biological samples, especially plasma, under oxidative stress. This assay quantifies the ability of antioxidants to neutralize free radicals generated by a water-soluble azo compound such as 2,2'-azobis(2-amidinopropane) dihydrochloride (ABAP), which produces ROO• at 37 °C. The extent of antioxidant activity is determined by measuring the lag phase in oxygen consumption inhibition, with the induction period monitored via an oxygen electrode. Results are typically expressed as Trolox equivalents, reflecting the molar amount of radicals trapped per liter of sample (Wayner et al., 1985). The TRAP assay has shown that total plasma antioxidant capacity often exceeds the sum of known antioxidants like vitamin C, urate, and vitamin E, indicating the presence of unidentified antioxidant components (Nälsén et al., 2006). The method is particularly responsive to chain-breaking antioxidants and can measure scavenging efficiency against a variety of radicals, including peroxyl radicals, hydroxyl radicals, and peroxynitrite. Fluorescent probes such as β-phycoerythrin, dichlorofluorescein (from DCFH-DA), and ABTS have also been employed in TRAP-based approaches, with antioxidant activity inferred from the inhibition of fluorescence or absorbance (Dasgupta & Klein, 2014). Although highly informative, the assay's complexity and time requirements often limit its widespread routine use (Yamada et al., 2016).

Despite these limitations, the TRAP assay remains a powerful tool in both clinical and research settings, offering detailed insights into antioxidant dynamics in health and disease. It has been widely applied in physiological studies, such as assessing oxidative stress during physical exertion. For instance, in a study involving firefighters, the TRAP assay effectively tracked oxidative changes induced by strenuous activity, demonstrating its potential as a biomarker for oxidative imbalance (Park et al., 2016a, b). Additionally, its application in animal studies has validated its robustness in monitoring non-enzymatic antioxidant responses to exercise and other stressors (Godoy et al., 2022). The TRAP assay also correlates well with other antioxidant assays, such as FRAP and TEAC, reinforcing its credibility in evaluating antioxidant status (Lucas et al., 2016). Beyond physiological applications, TRAP has proven valuable in dietary studies by assessing the antioxidant potential of foods and identifying their role in preventing oxidative damage (Daneshzad et al., 2020). Moreover, elevated TRAP values have been associated with protective effects in sensitive tissues, such as the ovaries, where antioxidant defenses are essential for cellular preservation (Massignam et al., 2018). Overall, the TRAP assay, despite being relatively complex, remains indispensable for comprehensive antioxidant profiling, especially when precise measurement of plasma antioxidant capacity is required.

### Total oxyradical scavenging capacity (TOSC) assay

The TOSC assay is a robust and versatile analytical method developed to quantify the capacity of biological samples to neutralize ROS, which are implicated in oxidative stress and various pathological conditions (Regoli et al., 2000a, 2002). By evaluating the ability of antioxidants to suppress the formation of ethylene from α-keto-γ-methiolbutyric acid (KMBA) in the presence of ROS, the assay provides a nuanced measure of the antioxidant defenses against peroxyl radicals, hydroxyl radicals, and peroxynitrite (Kang et al., 2010; Kwon et al., 2010; Regoli et al., 2000a, b). ROS are typically generated via the thermal decomposition of ABAP, and the resulting ethylene is quantified through gas chromatography. The antioxidant capacity is then expressed in Trolox equivalents based on the area under the curve (AUC) of ethylene inhibition (Dasgupta & Klein, 2014; Kwon et al., 2009; Regoli et al., 2002). Despite its time-consuming nature, which may limit routine use (Magalhães et al., 2008), the TOSC assay’s strength lies in its sensitivity and ability to differentiate between fast- and slow-acting antioxidants, as well as its applicability to both hydrophilic and lipophilic compounds (Dasgupta & Klein, 2014).

The TOSC assay has been widely applied across diverse research fields, ranging from ecotoxicology and environmental monitoring to nutrition and clinical medicine. It has proven particularly useful in assessing antioxidant responses in marine organisms, where it correlates oxidative biomarkers with ecological stress (Regoli et al., 2003), and in seminal plasma, where it helps evaluate protection mechanisms for spermatozoa (Balercia et al., 2003). Additionally, it has been employed to analyze antioxidant potential in plant-based foods, beverages, and extracts, revealing significant variability in TOSC values and underlining their health-promoting properties (Kwon et al., 2009; Lichtenthäler & Marx, 2004; Sun et al., 2002). Clinically, it has facilitated the study of antioxidant defenses in human cells under oxidative stress, offering insights into cellular protection (Armeni et al., 2001), while its responsiveness to pollutants enhances its utility in environmental studies (Kwon et al., 2010; Regoli et al., 2003). Electrochemical adaptations have further expanded its applicability; for example, Salazar et al. used voltammetry and coulometric analysis to explore the oxidation of vanillin and isovanillin by ABAP-derived radicals, revealing mechanistic details via controlled-potential electrolysis (Haque et al., 2021). Comparative studies show a strong correlation between TOSC and other assays like ORAC (*r* = 0.77), though compounds such as rutin and α-lipoic acid exhibit greater activity in TOSC, emphasizing the assay’s complementary strength (Dasgupta & Klein, 2014). Overall, the TOSC assay remains an essential, sensitive, and insightful tool for understanding antioxidant systems in biological and environmental contexts.

### TEAC (trolox equivalent antioxidant capacity) assay

The TEAC assay is a widely utilized and reliable method for quantifying the antioxidant capacity of diverse substances, especially in food and biological samples. This assay measures the ability of antioxidants to scavenge the ABTS• +, with results expressed as Trolox equivalents—a water-soluble analog of vitamin E. The TEAC value represents the millimolar concentration of Trolox that matches the antioxidant capacity of the test sample (Huang et al., 2005; Klayraung & Okonogi, 2009). The assay’s operational simplicity and versatility have made it a preferred choice in many laboratories, as it involves generating the ABTS^•+^ and monitoring the decrease in absorbance at 734 nm upon sample addition, compared against a Trolox standard curve (Danila et al., 2011; Oliveira et al., 2008). Validated across multiple fields such as food science and pharmacology, the TEAC assay has also been employed to evaluate antioxidant capacity against peroxynitrite-induced oxidative reactions, including inhibition of tyrosine nitration and dihydrorhodamine oxidation, thereby offering insights into the efficacy of antioxidant compounds (Carocho & Ferreira, 2013; Magalhães et al., 2007; Zima et al., 2010).

One of the key strengths of the TEAC assay lies in its standardized and quantitative comparison of antioxidant activity relative to Trolox via ABTS radical decolorization, facilitating cross-study and sample comparisons. Its results correlate well with other widely used antioxidant assays such as FRAP and DPPH, enhancing confidence in its applicability (Chen et al., 2010; Tafulo et al., 2010). TEAC values from various foods have been linked to potential health benefits, as higher antioxidant capacities often correspond to protection against oxidative stress (Arbneshi et al., 2021; Qureshi et al., 2019). The assay has been broadly applied in research involving antioxidant evaluation of wines, chocolates, and medicinal herbs, demonstrating its extensive utility (Klayraung & Okonogi, 2009; Nahuelcura et al., 2023; Tahmaz & Söylemezoğlu, 2017). Recent advancements include the adoption of automated, high-throughput systems that improve measurement efficiency and precision, alongside adaptations for analyzing complex matrices such as biological fluids and food extracts, providing comprehensive antioxidant profiles (Magalhães et al., 2007; Rubio et al., 2016a, b; Tran et al., 2024).

### The FC method

The FC assay, originally developed for protein quantification, has become a widely adopted method for assessing TAC in food and biological samples. The classical method, established by Singleton et al. (1999), is based on the electron transfer from phenolic compounds to the FC reagent, which consists of phosphomolybdate and phosphotungstate. This reaction reduces Mo(VI) to Mo(V), producing a blue complex with a characteristic absorbance at 765 nm. Antioxidant capacity is typically expressed as gallic acid equivalents (GAE). However, the original FC assay primarily measures hydrophilic antioxidants due to the aqueous nature of the reagent. To address this limitation, Berker et al. (2013) modified the protocol by introducing an isobutanol–water medium with added NaOH, allowing for the simultaneous determination of both hydrophilic and lipophilic antioxidants. The optimized conditions-1:2 FC-to-isobutanol ratio, 3.5 × 10⁻^2^ M NaOH, 20-min reaction time, and 665 nm detection-enabled accurate quantification of compounds such as gallic acid, quercetin, catechin, ascorbic acid, Trolox, α-tocopherol, and β-carotene. This modified version demonstrated good reproducibility, additive behavior in complex mixtures, and strong correlation with other assays like the cupric reducing antioxidant capacity (CUPRAC) method.

In parallel, electrochemical techniques have emerged as rapid and selective alternatives to the FC assay, especially for analyzing total phenolic content in complex matrices such as wines. Techniques like cyclic voltammetry (CV) and differential pulse voltammetry (DPV) can differentiate phenolic subgroups-including hydroxycinnamic acids, catechins, and anthocyanins-based on their distinct oxidation potentials. Additionally, laccase-based biosensors offer amperometric detection of phenolics with high correlation to FC-derived measurements. These electrochemical methods overcome the lack of specificity inherent in the FC assay and provide real-time, high-resolution analysis, making them particularly valuable in food quality control and phenolic profiling (Haque et al., 2021). Collectively, these advancements enhance the analytical scope and precision of TAC measurements in diverse research and industrial applications.

### The crocin bleaching assay (CBA)

The CBA, initially developed by Bors et al. (1984), is a widely utilized method for assessing antioxidant capacity based on the ability of compounds to inhibit the bleaching of crocin, a water-soluble carotenoid pigment derived from saffron, in the presence of peroxyl radicals. These radicals are typically generated by the thermal decomposition of ABAP. The principle of the assay lies in the inverse relationship between crocin bleaching and antioxidant activity: antioxidants scavenge the radicals, thereby preventing crocin oxidation. Antioxidant capacity is determined spectrophotometrically by measuring changes in absorbance at 440–443 nm, with results expressed as the ratio of bleaching rates (V₀/V) or percentage inhibition of crocin bleaching (%In), which enables IC₅₀ determination—a key metric for comparing antioxidant potency (Assis et al., 2015; Dasgupta & Klein, 2014; González et al., 2021; Prieto et al., 2015; Tonolo et al., 2019). The assay is particularly suited for water-soluble antioxidants and is commonly applied in human plasma and natural product research. However, limitations such as interference from food pigments, pH sensitivity, metal ion effects, and issues with reproducibility have been reported (Magalhães et al., 2008; Prieto et al., 2015; Danet et al., 2023).

To address these shortcomings, several methodological advancements have been made. Prieto et al., (2015) developed a refined version of the CBA using a microplate format and a nonlinear kinetic model, allowing simultaneous evaluation of antioxidant and pro-oxidant behaviors over time and across concentration ranges. This model improved reproducibility and provided deeper mechanistic insights into redox modulation, demonstrating, for instance, that Mn^2^⁺, Cu⁺, and Cu^2^⁺ exert significant antioxidant effects, whereas BHT and α-tocopherol showed no activity under the same conditions. In addition, electrochemical adaptations of the CBA have enhanced sensitivity and selectivity. Using square wave voltammetry, crocin oxidation can be monitored via its distinct anodic peak at + 670 mV on a glassy carbon electrode, which declines exponentially upon exposure to radicals, following pseudo-first-order kinetics. This electrochemical variant offers greater analytical resolution by distinguishing crocin’s redox signal from matrix interferences, especially in complex samples like saffron extracts (Haque et al., 2021). Furthermore, coupling the CBA with high-performance liquid chromatography (HPLC) has expanded its utility in detecting antioxidant compounds in complex matrices, making it a valuable tool in food chemistry, pharmacology, and natural product research (Bountagkidou et al., 2012; Ghafari et al., 2023; Zeka et al., 2020).

### Lipid peroxidation assessment assays

Lipid peroxidation is a critical process implicated in numerous pathological conditions, characterized by the oxidative degradation of PUFAs in cellular membranes. This reaction, initiated by ROS, abstracts hydrogen atoms from PUFAs and propagates a damaging chain reaction that compromises membrane integrity, disrupts signaling pathways, and contributes to the progression of diseases such as atherosclerosis, neurodegeneration, and cancer (Dodson et al., 2019; Iuchi et al., 2021; Li et al., 2022). The resulting formation of reactive aldehydes like malondialdehyde (MDA) and 4-hydroxy-2-nonenal (4-HNE) serves as a hallmark of oxidative stress and lipid peroxidation (Dodson et al., 2019; Peiro et al., 2005a, b). These byproducts exert cytotoxic effects, induce apoptosis, and exacerbate cellular dysfunction (Iuchi et al., 2021). To assess lipid peroxidation, various assays have been developed. The thiobarbituric acid reactive substances (TBARS) assay remains a widely used method that quantifies MDA through its reaction with thiobarbituric acid (TBA), forming a pink MDA–(TBA)₂ complex measurable at 532 nm, providing a practical marker of oxidative damage in both biological and food systems (Danet, 2021a, b). However, the specificity and sensitivity of lipid peroxidation measurements have been significantly improved by advances in high-performance liquid chromatography (HPLC), mass spectrometry (MS), and nuclear magnetic resonance (NMR) spectroscopy (Dodson et al., 2019; Phan et al., 2020). HPLC enables the precise quantification of lipid hydroperoxides, while MS, particularly with Na⁺ adducts and collision-induced dissociation (CID), facilitates the identification of hydroperoxide positions and diastereomers, enhancing our understanding of lipid oxidation in vivo (Kontogianni & Gerothanassis, 2022; Peiro et al., 2005a, b; Zhu et al., 2011). NMR techniques, such as 1D-TOCSY and band-selective ^1^H-^13^C HSQC, further augment resolution and structural elucidation of lipid oxidation products, with in-cell NMR and saturation transfer difference (STD) spectroscopy enabling the study of lipid–protein interactions (Kontogianni & Gerothanassis, 2022). Integration of chemical shift databases, density functional theory (DFT) calculations, and automated platforms like LC-SPE-NMR-MS offers comprehensive structural insights. Future advancements, including ion-mobility MS, miniaturized LC–MS systems, and standardized analytical methodologies, alongside bioinformatics resources like LIPID MAPS, are poised to propel oxidative lipidomics forward. Collectively, these innovations not only improve the accuracy of lipid peroxidation assessment but also enhance our understanding of its pathological relevance, ultimately supporting the development of targeted therapeutic strategies (Dodson et al., 2019; Kontogianni & Gerothanassis, 2022; Phan et al., 2020).

### Ferrous oxidation-xylenol orange (FOX)

The FOX assay is a widely utilized method for quantifying lipid hydroperoxides, which are critical markers of oxidative stress and lipid peroxidation in various biological systems. The assay operates on the principle that under acidic conditions, lipid hydroperoxides oxidize ferrous ions (Fe^2^⁺) to ferric ions (Fe^3^⁺), which then form a colored complex with xylenol orange, that is measured spectrophotometrically, typically around 560 nm (Duca et al., 2019; Grau et al., 2000; Pinto et al., 2007). This method has gained popularity due to its sensitivity, rapid execution, and low cost, making it suitable for high-throughput applications (Chrisnasari et al., 2024; Kontogianni & Gerothanassis, 2022).

One of the significant advantages of the FOX assay is its adaptability to different sample types, including plant extracts, animal tissues, and food products. For instance, Pinto et al. (2007) successfully employed a modified FOX assay to determine lipoxygenase activity in plant extracts, demonstrating its versatility in biochemical applications. Similarly, DeLong et al. (2001) utilized the FOX assay to quantify lipid hydroperoxides in plant tissues, further highlighting its applicability in agricultural and food chemistry. Moreover, the FOX assay's ability to detect low concentrations of hydroperoxides makes it a valuable tool in clinical settings, as evidenced by its use as an oxidative stress biomarker in patients with pulmonary hypertension (Reis et al., 2013).

Despite its advantages, the FOX assay is not without limitations. The linear detection range can be narrow, which may complicate the quantification of hydroperoxide levels in samples with high concentrations (Chrisnasari et al., 2024). This limitation has prompted researchers to explore modifications to the assay to enhance its sensitivity and broaden its detection range. For example, Chrisnasari et al. (2024) proposed modifications to the FOX assay to improve its capacity for high-throughput screening of lipoxygenase activity, addressing the challenges associated with the original method. Additionally, the FOX assay's susceptibility to interference from other reactive species necessitates careful consideration when interpreting results, particularly in complex biological matrices (Kontogianni & Gerothanassis, 2022).

### TRAA (tocopheroxyl radical attenuating ability)

The study of antioxidants and their mechanisms has evolved significantly over the years, with advancements in analytical methods enabling more rapid, sensitive, and reliable screening of potential inhibitors of oxidative processes. A notable example is the development of a rapid and convenient method for screening inhibitors of low-density lipoprotein (LDL) oxidation, as reported by Witting et al. (1996). This method utilizes micelles of cetyltrimethyl ammonium chloride or sodium dodecyl sulfate, combined with alpha-tocopherol, to measure the attenuation of alpha-tocopheroxyl radicals generated by UV irradiation. The radicals are detected directly using electron spin resonance (ESR) spectroscopy, and the alpha-tocopheroxyl radical attenuating ability (TRAA) of 53 natural and synthetic antioxidants was compared with their ability to inhibit LDL lipid oxidation. The results demonstrated a highly significant correlation (*P* < 0.00005) between TRAA and LDL antioxidation activity, suggesting that the TRAA test alone could predict the potency of co-antioxidants for LDL's alpha-tocopherol with > 98% accuracy. This method represented a significant improvement over traditional approaches, offering a simple and effective screening tool for large-scale studies (Witting et al., 1996).

In parallel, the antioxidant assay described by Burton and Ingold (1981) investigates the effectiveness of tocopherols (Vitamin E compounds) in inhibiting lipid peroxidation, a process linked to cell damage caused by free radicals. This assay evaluates the antioxidant activity of four tocopherol isomers-α-tocopherol (α-T), β-tocopherol (β-T), γ-tocopherol (γ-T), and δ-tocopherol (δ-T)-each differing in their chemical structure, such as methyl group placement. While α-tocopherol is widely recognized for its in vivo effectiveness, its in vitro potency compared to other tocopherols has been debated. The assay monitors autoxidation reactions initiated by radicals, measuring inhibition through methods like oxygen uptake and induction periods in the presence of tocopherols. These antioxidants interrupt the chain reaction of lipid peroxidation by reacting with peroxyl radicals, potentially shortening oxidation chains and terminating the process (Dasgupta & Klein, 2014). The study uses styrene as a substrate and azoisobutyronitrile (AIBN) as a radical initiator to carefully monitor oxidation rates, emphasizing the importance of controlling factors like impurities and chain transfer reactions to ensure accurate comparisons of antioxidant properties. Together, these methods provide valuable insights into the mechanisms of action of antioxidants and their potential therapeutic benefits in mitigating oxidative stress and cellular damage.

In contrast, traditional methods for evaluating antioxidant activity, such as the ORAC, FRAP, and DPPH assays, often require complex sample preparation and long analysis times. These methods are further limited by their reliance on non-physiological conditions, making it difficult to extrapolate results to in vivo environments (Aruoma, 2003). Aruoma (2003) emphasized the need for a consensus on in vitro antioxidant methods, advocating for a combination of assays to better understand the mechanisms involved and to ensure bio-efficacy through reliable in vivo models. This highlights the limitations of older methods and the necessity for more advanced techniques.

The evolution of antioxidant analysis methods, from the TRAA test and tocopherol-mediated inhibition assays to advanced techniques like supercritical fluid extraction-supercritical fluid chromatography (SFE/SFC) coupled with proton transfer reaction ionization mass spectrometry (PTR-MS), reflects the growing need for rapid, sensitive, and physiologically relevant tools. These advancements pave the way for a deeper understanding of antioxidant mechanisms and their applications in health and disease management.

A significant leap forward in antioxidant analysis is demonstrated by the work of Ota et al. (2022), who developed a rapid and sensitive method for analyzing alpha-tocopherol and its oxidative products using supercritical fluid extraction-supercritical fluid chromatography (SFE/SFC) coupled with proton transfer reaction ionization mass spectrometry (PTR-MS), enabling the separation and detection of alpha-tocopherol and its oxidation products, such as the alpha-tocopheroxyl radical and alpha-tocopheryl quinone, in approximately 3 min per sample with high sensitivity and resolution, even for isomers, without extensive sample preparation-a substantial improvement over traditional chromatographic methods that often involve complex processing and long separation times. This method's ability to rapidly analyze complex matrices, such as cells and tissues, makes it particularly valuable for studying antioxidant defense mechanisms in plant cells during photosynthesis (Ota et al., 2022), aligning with the findings of Hondo et al. (2022), who highlighted its advantages in speed, sensitivity, and applicability to complex biological systems. While the TRAA test provides reliable screening for LDL lipid peroxidation inhibitors, it is limited in scope compared to the SFE/SFC-PTR MS method, which not only simplifies analysis but also expands research possibilities by enabling real-time study of cellular mechanisms and oxidative products. The progression from the TRAA test to advanced techniques like SFE/SFC-PTR MS underscores the increasing demand for rapid, sensitive, and physiologically relevant tools, paving the way for deeper insights into antioxidant mechanisms and their applications in health and disease management.

### β-carotene bleaching assay

The β-carotene bleaching assay is a widely used method to evaluate the antioxidant potential of various compounds, particularly in lipid-rich systems. This assay is based on the principle of competitive inhibition, where antioxidants protect β-carotene from oxidation by neutralizing free radicals generated from linoleic acid. In the absence of antioxidants, β-carotene undergoes rapid oxidation, leading to its bleaching, which is measured spectrophotometrically at 470–490 nm (Álvarez-Parrilla et al., 2012; Yang et al., 2008a, b). The assay is particularly effective for screening lipophilic antioxidants, such as flavonoids and phenolic compounds, which are commonly found in plant extracts and food systems (Alasalvar et al., 2001; Christodoulou et al., 2022).

The method involves preparing an emulsion of β-carotene, linoleic acid, and an emulsifier (e.g., Tween 80) in chloroform, followed by the evaporation of the solvent and the addition of oxygenated distilled water. The antioxidant solution is then mixed with the emulsion, and the mixture is incubated at 50 °C for a specific period, typically 2–4 h. The rate of β-carotene bleaching is monitored over time, and the antioxidant activity is calculated based on the inhibition of β-carotene oxidation (Prieto et al., 2012). The bleaching rate (R) is determined using first-order kinetics, and the antioxidant activity (AA) is expressed as the percentage inhibition of β-carotene bleaching relative to a control (Christodoulou et al., 2022; Shahidi & Yeo, 2012).

One of the key strengths of this assay is its ability to simulate lipid peroxidation, a process highly relevant to oxidative stress in biological systems. This makes the β-carotene bleaching assay particularly useful for evaluating the efficacy of antioxidants in preventing oxidative damage in food, pharmaceuticals, and cosmetics (Yang et al., 2008a, b). However, the assay has certain limitations, including sensitivity to experimental conditions such as temperature, pH, and the presence of metal ions, which can affect the reproducibility of results (Dawidowicz & Olszowy, 2015). Additionally, the assay has been criticized for its low correlation with other antioxidant assays, such as FRAP and DPPH, which can lead to inconsistent findings (Christodoulou et al., 2022).

Despite these challenges, the β-carotene bleaching assay remains a valuable tool for comparative studies of antioxidant efficacy. It has been successfully applied to characterize the antioxidant properties of natural extracts, such as those from *Melodinus eugeniifolus*, where ethanol extracts of bark and leaves demonstrated significant antioxidant activity (Lu et al., 2014). Furthermore, modifications to the assay, such as the development of microplate-based techniques, have improved its reproducibility and expanded its applicability for high-throughput screening of antioxidant and prooxidant activities (Prieto et al., 2012).

The β-carotene bleaching assay is a simple, yet effective, method for evaluating the antioxidant potential of lipophilic compounds. While it has certain limitations, its ability to simulate lipid peroxidation and its relevance to biological systems make it a valuable tool in antioxidant research. Ongoing advancements, such as the development of microplate-based methods, continue to enhance its utility and reliability in various applications.

### Conjugated diene assay

The conjugated diene (CD) assay is a widely recognized and reliable method for evaluating lipid peroxidation, particularly within biological systems. This assay is based on the principle that conjugated dienes are among the earliest detectable products formed during the oxidation of lipids, especially polyunsaturated fatty acids (PUFAs). These conjugated double bonds, formed during peroxidation, exhibit a distinct absorbance peak at approximately 233–234 nm, which is exploited for spectrophotometric analysis (Carru et al., 2004; Shin & Park, 2007).

Lipid peroxidation is initiated by ROS, which abstract hydrogen atoms from PUFAs in cell membranes and lipoproteins, leading to the formation of lipid radicals, hydroperoxides, and ultimately conjugated dienes (Nanetti et al., 2008; Yang, 2012). The CD assay is thus a sensitive indicator of oxidative stress and a valuable tool for assessing the antioxidant potential of compounds. Various antioxidants, including herbal extracts, have been shown to inhibit the formation of conjugated dienes, thereby mitigating oxidative damage (Kūka et al., 2018; Patel et al., 2014).

In clinical research, the CD assay is frequently used to determine the susceptibility of low-density lipoproteins (LDL) to oxidation, a key contributor to the development of atherosclerosis. Elevated levels of conjugated dienes in LDL are strongly associated with increased cardiovascular risk (Shin & Park, 2007; Yu-Poth et al., 2000). Dietary interventions, such as reduced intake of saturated fats, have demonstrated effectiveness in lowering LDL oxidation, as indicated by reduced diene formation (Schaich et al., 2013; Yu-Poth et al., 2000).

Beyond human health, the CD assay plays an important role in food science, particularly in monitoring the oxidative stability of oils and fats during processing and storage. The appearance of conjugated dienes serves as a key indicator of lipid degradation and rancidity in edible oils, making this assay a critical component of quality control in the food industry (Farhoosh et al., 2012; Kūka et al., 2018; Megahed, 2001).

A notable application of the CD assay is found in the recent work of Sidorov et al. (2024), who investigated lipid profile changes in the microalga *Vischeria* sp. IPPAS C-70 under oxidative stress induced by hydrogen peroxide (H₂O₂). Using thin-layer chromatography (TLC), HPLC–DAD, and GC–MS, they identified nine novel isomers of hexadecadienoic acid (C16:2), including methylene-interrupted, conjugated, and even allene-type dienes. Under oxidative stress, conjugated dienes increased dramatically from 8% to 28.5% of total fatty acids. Simultaneously, saturated fatty acids such as palmitic acid rose, while unsaturated fatty acids like palmitoleic acid declined, suggesting suppressed desaturase activity. These findings point to the potential role of structurally unique fatty acids in stress adaptation, though their biosynthetic origins and physiological significance require further investigation.

The standard procedure for the CD assay involves extracting lipids from biological samples such as membranes or human serum using a chloroform: methanol mixture (2:1, v/v). After phase separation, the organic layer is collected, dried under an inert gas (e.g., nitrogen), and reconstituted in cyclohexane, which is suitable for UV–Vis analysis. The absorbance is then measured at 233 nm, with the intensity correlating directly to the concentration of conjugated dienes present (Esterbauer et al., 1991; Gutteridge & Halliwell, 1990; Janero, 1990; Pryor et al., 1976).

Its simplicity, sensitivity, and broad applicability, the conjugated diene assay remains a cornerstone in lipid peroxidation studies. It provides essential insights into early oxidative damage, the efficacy of antioxidants, and the stability of lipid-rich products in biomedical and food science research (Esterbauer & Cheeseman, 1990).

### LDL oxidation inhibition assay

The \low-density lipoprotein (LDL) oxidation inhibition assay is a critical experimental approach used to evaluate the antioxidant properties of various compounds and their potential to inhibit LDL oxidation, a key factor in the development of atherosclerosis (Seo et al., 2014; Yang et al., 2016a, b). This assay typically involves measuring the formation of TBARS as a marker of lipid peroxidation, which is indicative of LDL oxidation (Jeong et al., 2007; Lin et al., 2006). The significance of LDL oxidation lies in its association with cardiovascular diseases, where oxidized LDL (oxLDL) can trigger inflammatory responses and foam cell formation, contributing to the development of atherosclerotic plaques (Tani et al., 2011; García‐Gómez et al., 2023). Uses low-density lipoproteins (LDL) as the oxidizable target. It measures conjugated diene formation at 234 nm. High inter-batch variability due to LDL isolation requirements (Cataldo et al., 2018; Magalhães et al., 2008)).

The inhibition of LDL oxidation assay measures the TAC of natural product extracts. In this method, human plasma is used to isolate LDL, and ROO• generated from the thermal decomposition of water-soluble ABAP at 37 °C are used to oxidize LDL (Chu & Liu, 2004). The extent of LDL peroxidation is assessed by measuring hexanal production using headspace gas chromatography. Hexanal is formed due to the oxidation of PUFAs in LDL, which can also be initiated using copper ions (Nakajima et al., 2007; Satchell & Leake, 2012). The antioxidant capacity of samples is compared to standard antioxidants such as vitamin E and vitamin C, providing a reliable measure of free radical scavenging potential (Chu & Liu, 2004; Wu et al., 2009; Shahidi & Zhong, 2010).

Various studies have demonstrated the efficacy of natural and synthetic antioxidants in inhibiting LDL oxidation. For instance, Lee et al. (2006) reported that abietane diterpenoids isolated from *Torreya nucifera* leaves exhibited significant antioxidant activities, effectively inhibiting lipid peroxidation. Similarly, Kim et al. (2012) highlighted those extracts from *Scutellariae Radix* not only inhibited LDL oxidation but also reduced inflammatory responses in macrophages, suggesting a dual role in antioxidant protection and anti-inflammatory effects. The TBARS assay was employed in these studies to quantify the extent of lipid peroxidation, reinforcing its utility in assessing antioxidant efficacy (Seo et al., 2014; Yang et al., 2016a, b).

The mechanisms through which antioxidants exert their protective effects against LDL oxidation are diverse. Polyphenolic compounds found in balsamic vinegar, for example, have been shown to inhibit the activity of enzymes that promote LDL oxidation, such as lipoxygenases and myeloperoxidase (Iizuka et al., 2010). Flavonoids, on the other hand, scavenge free radicals and chelate transition metal ions, thereby preventing the oxidative modification of LDL (Wu et al., 2009). Additionally, the antioxidant capacity of high-density lipoprotein (HDL) has been assessed using assays that measure its ability to prevent LDL oxidation, highlighting the complex interplay between different lipoproteins in the cardiovascular system (García‐Gómez et al., 2023).

The assay can be influenced by various factors, including the oxidative state of LDL and the presence of transition metals such as copper and iron, which catalyze LDL oxidation (Arai et al., 2005; Carru et al., 2011). For instance, the oxidation of LDL by copper ions can be monitored spectrophotometrically, providing a quantitative measure of antioxidant activity (Nakajima et al., 2007; Vasilescu et al., 2013). These findings underscore the importance of understanding the biochemical pathways involved in LDL oxidation and the potential therapeutic implications of antioxidant compounds in preventing cardiovascular diseases (Satchell & Leake, 2012; Tani et al., 2011).

The LDL Oxidation Inhibition Assay serves as a vital tool in the evaluation of antioxidant compounds, providing insights into their mechanisms of action and potential health benefits. The collective evidence from various studies highlights the significance of inhibiting LDL oxidation as a strategy for mitigating the risk of atherosclerosis and related cardiovascular conditions (Seo et al., 2014; Yang et al., 2016a, b).

### The inhibition of linoleic acid oxidation (ILAO) assay

The ILAO assay, developed by Niki et al. (1984), is a widely used method for evaluating the antioxidant capacity of food or natural product extracts. Unlike methods that assess LDL oxidation in vitro, the ILAO assay measures the oxidation of methyl linoleate in the presence of ROO• generated by the decomposition of an azo compound. Lipid oxidation, particularly of polyunsaturated fatty acids, has been extensively studied due to its role in food deterioration and its association with pathological conditions such as inflammation, platelet aggregation, asthma, and oxidative cellular damage (Dasgupta & Klein, 2014).

In this assay, methyl linoleate undergoes oxidation in solution, and the inhibition of this process by antioxidants such as vitamin E and vitamin C is analyzed. The rate of oxidation is monitored by measuring oxygen concentration using a Biological Oxygen Monitor or by tracking pressure changes due to oxygen uptake with a gas absorption apparatus. The reaction is conducted in a temperature-controlled environment (37 or 50 °C), and the concentrations of antioxidants are determined using high-performance liquid chromatography (HPLC). This method provides valuable insights into the effectiveness of antioxidants in preventing lipid peroxidation (Dasgupta & Klein, 2014).

The ILAO assay is based on the principle that antioxidants can inhibit the oxidation of linoleic acid, a polyunsaturated fatty acid highly susceptible to oxidative degradation. This inhibition prevents the formation of harmful oxidation products. The assay often employs a β-carotene-linoleic acid system, where the discoloration of β-carotene serves as an indicator of the oxidative process, allowing for the quantification of antioxidant activity. As linoleic acid oxidizes, it generates free radicals and other reactive species that degrade β-carotene, leading to a decrease in its color intensity. Antioxidants in the sample scavenge these free radicals, slowing down the oxidation process and preserving the color of β-carotene. This method has been validated in numerous studies, demonstrating its effectiveness in mimicking lipid peroxidation in biological systems (Silva et al., 2012; Sökmen & Khan, 2016).

Research has shown that various plant extracts exhibit differing degrees of antioxidant activity as measured by the ILAO assay. For example, extracts from *Myristica fragrans* and *Vitex negundo* have been evaluated for their ability to inhibit linoleic acid oxidation, with significant results highlighting their potential as natural antioxidants (Din et al., 2021; Saklani et al., 2017). Similarly, flavonoid-rich extracts from plants such as *Hypericum perforatum* and *Iris germanica* have demonstrated notable inhibition of linoleic acid peroxidation, further underscoring the utility of the ILAO assay in assessing antioxidant properties (Ullah et al., 2015; Zou et al., 2004).

The ILAO assay has also been used to compare the antioxidant efficacy of synthetic antioxidants, such as BHT, with natural compounds. For instance, extracts from *Swertia chirata* exhibited a high percentage of inhibition in linoleic acid oxidation, comparable to that of BHT, emphasizing the potential of natural antioxidants in food preservation and health applications (Naqvi et al., 2013; Touaibia, 2017). Beyond food science, the assay provides insights into the protective effects of antioxidants against oxidative stress in biological systems, which is implicated in various diseases (Boumerfeg et al., 2012; Gao et al., 2009).

### Inhibited oxygen uptake method

The inhibited oxygen uptake method is a critical assay used to evaluate the effects of various substances on cellular respiration and mitochondrial function, particularly in pharmacological studies. This method measures the rate of oxygen consumption in response to specific inhibitors, providing insights into the impact of these agents on mitochondrial respiration and their potential therapeutic efficacy (Madrigal‐Pérez et al., 2016; Wheaton et al., 2014). For instance, compounds like resveratrol have been shown to reduce oxygen consumption in *Saccharomyces cerevisiae* and rat mitochondria at concentrations of 10–25 µM, suggesting its primary action involves inhibiting respiration (Madrigal‐Pérez et al., 2016). Similarly, metformin inhibits mitochondrial complex I, reducing oxygen consumption in cancer cells, which is crucial for understanding its role in tumor metabolism (Wheaton et al., 2014; Zannella et al., 2013). These findings highlight the importance of mitochondrial inhibition in the therapeutic actions of various compounds. Additionally, the presence of nitric oxide (NO) can competitively inhibit cytochrome c oxidase, a key enzyme in the mitochondrial respiratory chain, further modulating oxygen consumption and influencing tissue oxygenation, particularly in conditions like heart failure (Pérez‐Rojas & Muriel, 2005; Cooper & Giulivi, 2007; Traverse et al., 2002). Environmental factors and metabolic conditions, such as the presence of ROS or compounds like quercetin, can also modulate oxygen uptake, either promoting or inhibiting it through mechanisms like NADH reduction or ADP phosphorylation inhibition (Buss et al., 2005). This dual role underscores the complexity of metabolic regulation and its implications for therapeutic strategies.

### Scavenging of ferric thiocyanate (FTC) assay

The FTC method, adapted from Osawa (1991), is a widely used technique to assess the antioxidant activity of compounds based on their ability to inhibit lipid peroxidation. This method evaluates the capacity of antioxidants to prevent the oxidation of linoleic acid, a process that generates peroxides, which are then detected using ferric thiocyanate (Osawa, 1991). The assay involves preparing a sample mixture containing linoleic acid, phosphate buffer (pH 7.0), and distilled water, which is incubated at 40 °C in a dark container to simulate oxidative conditions.

During the reaction, ammonium thiocyanate and ferrous chloride are added to the sample mixture, leading to the formation of a red ferric thiocyanate complex. The intensity of this complex, measured spectrophotometrically at 500 nm, is proportional to the extent of lipid peroxidation (Osawa, 1991). A control sample, without the test compound, is used to represent 100% lipid peroxidation, while α-Tocopherol is often employed as a reference standard for antioxidant activity.

The FTC method is particularly useful for evaluating the ability of compounds to inhibit lipid peroxidation, providing insights into their potential antioxidant properties (Osawa, 1991). It is widely applied in food science, pharmaceuticals, and cosmetic industries to assess the efficacy of natural and synthetic antioxidants in preventing oxidative degradation of lipids (Osawa, 1991). This method is valued for its simplicity and reliability in measuring antioxidant activity, making it a valuable tool for screening compounds with potential protective effects against oxidative stress.

### Metal chelating assays

Metal chelating assays are essential tools for evaluating the ability of compounds to bind metal ions, particularly in the context of antioxidant activity. Excess free metal ions, such as iron (Fe^2^⁺), can catalyze the formation of ROS through Fenton reactions, leading to oxidative stress and cellular damage (Kotha et al., 2022; Wong et al., 2014). The Fenton reaction describes how ferrous ions (Fe^2^⁺) react with hydrogen peroxide (H₂O₂) to produce highly reactive hydroxyl radicals (•OH), which contribute to oxidative stress:

$$\mathrm{Fe}^{2+}\;+\;{\mathrm H}_2{\mathrm O}_2\;\rightarrow\;\mathrm{Fe}^{3+}\;+\;\mathrm{HO}^-\;+\;{}^{\cdot\bullet}\mathrm{OH}\;$$

This reaction is a major source of oxidative damage in biological systems, as the hydroxyl radicals can attack lipids, proteins, and DNA. Metal chelators mitigate this damage by forming stable complexes with metal ions, thereby preventing their participation in redox reactions. For instance, medicinal plant extracts have been tested for their metal chelating activities, with results showing concentration-dependent chelation capabilities. Among the tested extracts, *C. nutans* and *H. diffusa* exhibited the strongest chelating activities, while *C. formosana* and *L. cardiaca* showed weaker effects (Chelliah & Oh, 2022; Wong et al., 2014). These findings highlight the potential of natural compounds in managing metal-induced oxidative stress.

Metal chelation is a critical mechanism in biological systems, as transition metals like iron, copper, and zinc are essential for enzymatic functions but can become toxic at elevated levels. Excessive metal accumulation can lead to oxidative stress, damaging lipids, proteins, and DNA (Gulcin & Alwasel, 2022). Chelation therapy is often employed to treat metal toxicity by forming stable, ring-like complexes with metal ions, facilitating their removal from the body. Natural antioxidants, such as polyphenols, are particularly effective metal chelators due to their functional groups (e.g., hydroxyl, carbonyl) that bind metal ions. For example, taxifolin, a flavonoid, chelates Fe^2^⁺ through its 4-oxo, 5-OH, and catechol groups, preventing the formation of harmful iron-ferrozine complexes:

$$\mathrm{Taxifolin}\;+\;\mathrm{Fe}^{2+}\;\rightarrow\;\mathrm{Taxifolin}-\mathrm{Fe}^{2+}\;\mathrm{complex}$$

Similarly, curcumin, a compound found in turmeric, binds Fe^2^⁺ via its hydroxyl and methoxy groups, demonstrating high binding affinity (Gulcin & Alwasel, 2022). These interactions prevent the formation of the Fe^2^⁺-ferrozine complex, reducing oxidative stress.

Metal chelating assays typically involve spectrophotometric methods to measure the formation of metal–ligand complexes. Ferrozine and 2, 2′-bipyridine are commonly used reagents that form colored complexes with Fe^2^⁺ ions, detectable at specific wavelengths (562 and 485 nm, respectively). The reactions are as follows:

Formation of Fe^2^⁺-Ferrozine Complex:

$$3\mathrm{Ferrozine}+\mathrm{Fe}{({\mathrm H}_2\mathrm O)}_6^{2+}\rightarrow\mathrm{Fe}-{{(\mathrm{Ferrozine})}_3^{4-}+6{\mathrm H}_2\mathrm O\;\mathrm{Ferrozine})}_3^{4-}$$

The decrease in absorbance at 562 nm after adding a chelating agent indicates the formation of a metal-antioxidant complex, reflecting the chelating capacity of the sample.

Formation of Fe^2^⁺−2, 2′-Bipyridine Complex:

$$3\mathrm{Bipyridine}+\mathrm{Fe}{({\mathrm H}_2\mathrm O)}_6^{2+}\rightarrow\mathrm{Fe}-{(\mathrm{Bipyridine})}_3^{2+}+6{\mathrm H}_2\mathrm O$$

Similar to the ferrozine assay, the decrease in absorbance at 485 nm indicates the chelating activity of the sample.

Ethylenediaminetetraacetic acid (EDTA) is often used as a standard chelator due to its high affinity for metal ions. The chelating ability of samples is expressed as EDTA equivalents, providing a quantitative measure of their antioxidant potential (Kotha et al., 2022; Gulcin & Alwasel, 2022).

The IC50 value, which represents the concentration of a compound required to chelate 50% of metal ions, is a key parameter in evaluating chelating efficacy. Lower IC50 values indicate higher binding affinity and greater antioxidant potential (Gulcin & Alwasel, 2022; Chelliah & Oh, 2022). However, the interpretation of IC50 values must consider experimental conditions, as factors like temperature and pH can influence binding affinities. Despite these challenges, metal chelating assays remain valuable for assessing the antioxidant capacity of natural and synthetic compounds.

In biological systems, cooperative binding is another important concept, as seen in the binding of oxygen to hemoglobin. Hemoglobin binds oxygen cooperatively, with the binding of one oxygen molecule facilitating the binding of subsequent molecules (Kotha et al., 2022). This is an example of a cooperative effect in metal–ligand interactions:

$$\mathrm{Hemoglobin}\;(\mathrm{Hb})\;+\;4\;{\mathrm O}_2\;\rightleftharpoons\;\mathrm{Hemoglobin}-{({\mathrm O}_2)}_4\;(\mathrm{Oxyhemoglobin})$$

The binding of oxygen to the Fe^2^⁺ in the heme group of hemoglobin is a well-studied example of metal–ligand interactions in biological systems.

Metal chelating assays play a pivotal role in understanding the antioxidant mechanisms of compounds, particularly in mitigating metal-induced oxidative stress. Natural antioxidants, such as flavonoids and polyphenols, exhibit significant chelating activities, making them promising candidates for therapeutic and nutritional applications (Chelliah & Oh, 2022). Further research is needed to explore the in vivo efficacy of these compounds and their potential health benefits. Table 5 provides a comparison of in vitro antioxidant assays, highlighting their advantages, limitations, and specific applications.

**Table 5:** Comparison of in vitro antioxidant assays: advantages, limitations, and uses

Assay Name	Strengths	Weaknesses	Applications	Reference(s)
DPPH (2,2-diphenyl-1-picrylhydrazyl) radical scavenging assay	Simple, rapid, and widely used method Utilizes a stable free radical (DPPH) Does not require fresh preparation Highly convenient and efficient Quick, easy, and affordable Measures free radical scavenging activity Simple, fast, and reproducible Sensitive to phenolic compounds Does not require expensive reagents or sophisticated instruments	Interference from External Factors: Light, pH, and solvents can affect results Lack of Biological Relevance: May not accurately represent real biological conditions Time-Consuming Process: Requires extensive procedures and analysis Limited to Specific Radicals: May not reflect in vivo antioxidant activity Selective for Low-Polarity Compounds: Less effective for high-polarity substances Reaction Rate Dependence: Influenced by steric accessibility of the radical site Incomplete Representation: Does not fully reflect radical scavenging in cells or food	Assessment of Antioxidant Potential Foods, cosmetic products, and pharmaceuticals Environmental toxicology of plant extracts and synthetic compounds Determination of antioxidant properties in plant extracts and natural compounds Evaluation of Antioxidant Capacity Foods, beverages, and herbal extracts Pharmaceutical research for natural antioxidants Quality control in food and drug industries	Brand-Williams et al., 1995; Aruoma, 2003; Munteanu & Apetrei, 2021; Rumpf et al., 2023; Yang et al., 2008a, b; Özyürek et al., 2011; Rumpf et al., 2023; Baliyan et al., 2022; Gulcin & Alwasel, 2023
ABTS (2,2'-Azino-bis(3-ethylbenzothiazoline-6-sulfonic acid)) radical cation scavenging assay	High Sensitivity: Works for both hydrophilic and lipophilic antioxidants Simple & Low Cost: Easy to use and affordable Versatile: Suitable for various sample types Quantitative & Comparable: Provides reliable and reproducible results ABTS Radical Cation Assay: Minimizes color interference Cost-effective, fast, and reproducible Widely Used & Accepted: Commonly utilized in research laboratories Application in Storage & Processing: Effective for tracking antioxidant system changes over time	Susceptible to interference from factors like light, pH, and solvents Coupling reactions with certain antioxidants (e.g., phenolics) may bias results High stoichiometries and complex kinetics complicate interpretation Does not measure antioxidant reactivity or inhibition rates Results may vary between laboratories due to differences in experimental conditions	Measurement of TAC in food, biological samples, and plant extracts Evaluation of antioxidant changes during storage and processing Comparison of antioxidant capacity of different compounds (with reservations)	Re et al., 1999; Xie & Schaich, 2014; Rumpf et al., 2023; Özyürek et al., 2011; Rumpf et al., 2023; Ilyasov et al., 2020
FRAP (ferric reducing antioxidant power) assay	Simple, rapid, and cost-effective High sensitivity and precision Good reproducibility (CV = 0.74%) Excellent linearity (R^2^ = 0.99982) Suitable for routine analysis with low cost Simple and fast Useful for screening and comparing antioxidant capacities	Excludes certain important antioxidants, such as thiols (e.g., GSH) and albumin Limited to assessing reducing capacity Not suitable for lipophilic antioxidants Includes reductants that do not function as antioxidants, potentially leading to overestimation of antioxidant capacity Not responsive to specific antioxidants like GSH Does not reflect physiological pH and is conducted under non-physiological low pH conditions Does not account for antioxidants that act through hydrogen atom transfer (HAT) mechanisms Less effective for measuring the antioxidant capacity of thiol-containing compounds (e.g., GSH) Limited biological relevance; does not account for GSH Limited to electron transfer mechanism Does not account for radical scavenging Requires low pH for iron solubility	Evaluates the capacity of water-soluble antioxidants Widely used to measure the TAC of biological samples, foods, and beverages Applied to food samples and chitosan derivatives Screening antioxidant capacity of phenolic acids Predicting antioxidant activity using QSAR models Studying structure–activity relationships	Özyürek et al., 2011; Pinchuk et al., 2012; Rumpf et al., 2023; Spiegel et al., 2020
ORAC assay	Measures chain-breaking antioxidant activity, applicable to both hydrophilic and lipophilic antioxidants Flexible: can be performed manually or automatically Relatively rapid: takes about one hour High-throughput and adaptable, ideal for large-scale analysis Mimics biological oxidation processes, making it relevant for biological studies Widely used for determining antioxidant capacity of bioactive peptides Provides insights into radical–radical reactions and FLH repair mechanisms Useful for comparing antioxidant activity of different peptides High correlation with experimental data (R^2^ > 0.99) Quantifies antioxidant reactivity and capacity Provides mechanistic insights into antioxidant activity Can differentiate fast and slow antioxidants Applicable to both pure antioxidants and complex mixtures (e.g., fruit juices)	Not suitable for measuring lipid-soluble antioxidants, which can be potent Free radicals produced in vitro may lack physiological relevance Absence of standardization and variable value expression can influence results Analytical procedures significantly impact outcomes, making inter-laboratory comparisons of ORAC values challenging May not accurately mimic biological substrates Time-consuming Does not discriminate between peptide structures Complex chemistry involving secondary radicals and oxidation products ORAC values may not reflect simple reaction mechanisms AUC-based results can be ambiguous Requires precise control of experimental conditions Complex computations and interpretation Inter-laboratory variability in AUC values Limited by the assumption of constant radical generation rate (Ri)	Industrialists aiming to promote antioxidant properties of foods: Focus areas: Fruit and vegetable juices Wines Spices Human plasma Fruit Juice Mixed with Milk: Ascorbic acid as the primary contributor to antioxidant capacity Vegetable Beverages: Phenolic compounds and tocopherols as main contributors to antioxidant capacity ORAC (Oxygen Radical Absorbance Capacity) shows strong correlations with phenolic content Other Key Areas: Evaluation of antioxidant capacity of bioactive peptides Potential use in food preservation Research on structure–function relationships of antioxidant peptides Food science (e.g., fruit extracts) Pharmaceutical research Cosmetic and polymer industries Antioxidant reactivity assessment Structure–activity relationship studies Evaluation of antioxidant efficacy in real food systems	Matute et al., 2021; Magalhães et al., 2008; Barba et al., 2013; Figueroa et al., 2023; Asma et al., 2024; Pinchuk et al., 2012
CUPRAC (cupric ion reducing antioxidant capacity) assay	Stable and Physiological Conditions: Utilizes stable reagents and operates at neutral pH (pH 7), ensuring accurate results under physiological conditions Broad Detection Range: Effectively detects both hydrophilic and lipophilic antioxidants, making it suitable for diverse biological samples Reliability for Thiol Antioxidants: Particularly reliable for measuring thiol-type antioxidants, such as GSH Minimal Sensitivity to Dilution: Shows less sensitivity to dilution compared to other methods like ABTS, ensuring more consistent results Rapid and Non-Interfering Reaction: The reaction is rapid for most antioxidants and does not lead to interactions that could interfere with absorbance measurements High Sensitivity & Versatility: Applicable to a wide range of antioxidant types and biological samples Specificity & Accuracy: Effectively detects antioxidant activity in compounds that are challenging to measure with traditional assays Simplicity & Cost-Effectiveness: Easier and more affordable than sophisticated methods like HPLC	Limited biological relevance Inability to Measure Highly Reactive Species Sample Preparation Requirements Interference from Antioxidants Limited to Specific Analytes Technical Constraints	Fish Freshness Assessment: Used to enzymatically determine hypoxanthine (Hx) levels, a key freshness indicator in fish Food Industry Quality Control: Helps monitor fish spoilage over time, ensuring food safety and shelf-life determination Colorimetric Sensor for Hypoxanthine: Utilizes a CUPRAC-based membrane sensor to measure Hx levels in fish samples through absorbance at 450 nm Early Detection of Fish Degradation: Detects early stages of spoilage with high sensitivity, even at nanomolar concentrations of Hx Validated Against HPLC: Provides reliable results comparable to high-performance liquid chromatography (HPLC), confirming its accuracy Minimizes Interference: Effectively differentiates Hx from other biologically important antioxidants, such as cysteine, reduced GSH, ascorbic acid, uric acid, and α-tocopherol Real-Sample Applicability: Successfully tested on sea bass (*Dicentrarchus labrax*), making it suitable for real-world applications in seafood monitoring	Apak et al., 2004; Özyürek et al., 2011; Avan et al., 2023
β-Carotene bleaching assay	Simulates lipid peroxidation in biological systems Simple and cost-effective Effective for screening lipophilic antioxidants	Low reproducibility due to sensitivity to temperature, pH, solvents, and metal ions Poor correlation with other antioxidant assays (e.g., FRAP, DPPH, ABTS) Cut-off effect complicates interpretation of results	Evaluating antioxidant potential in food, pharmaceuticals, and cosmetics Screening natural extracts and synthetic antioxidants Comparing antioxidant efficacy in lipid-rich systems	Miller et al., 1993; Yang et al., 2008a, b; Prieto et al., 2012; Christodoulou et al., 2022; Dawidowicz & Olszowy, 2015
Total radical trapping antioxidant parameter(TRAP)	Chain-Breaking in Biological Systems: Antioxidants stop oxidative chain reactions in the body Plasma Antioxidant Status: Measures the level of antioxidants in plasma, indicating overall antioxidant capacity Synergistic Effects: Assesses how multiple antioxidants work together Disease Relevance: Used to evaluate antioxidant capacity in conditions like lupus, cancer, and diabetes	Slow Signal Recovery & Instability: Indicators like the oxygen electrode in TRAP show delayed responses and instability Lag Phase: Not all antioxidants show a lag phase, and it may not always be identifiable Limited OS Reflection: Doesn't fully reflect overall oxidative stress (OS) on its own Need for Additional Biomarkers: Requires other biomarkers for a complete OS assessment Study Limitations: Often limited by small sample sizes in research	Antioxidant Activity: Measures the ability to neutralize peroxyl radicals Sample Sources: Human plasma, grape seeds Applications: Evaluate oxidative status in MS (Multiple Sclerosis) patients Monitor antioxidant response to disease-modifying therapies (DMTs) Assess gender differences in antioxidant capacity	Özyürek et al., 2011; Magalhães et al., 2008; Kartau et al., 2024
TOSC assay	Comprehensive: Covers a wide range of antioxidant activity assessments Relevant for testing endogenous antioxidants (AOs) Focuses on radical chain-breaking mechanisms and provides a holistic measure of antioxidant capacity against multiple oxyradicals Predictive validity at the organism level Shows a high correlation with lysosomal damage (Neutral Red retention time), quantifying the overall capability of cellular antioxidants to neutralize various ROS Provides biological relevance by indicating the onset of cellular toxicity Applicable to different oxyradical-generating systems (ROO•, HO•, ONOOH) Widely used in ecotoxicology to assess oxidative stress in marine organisms	Labor-intensive and requires specialized equipment (e.g., gas chromatography) Involves complex sample preparation and separation of antioxidants Transient responses to pollutants may complicate interpretation Less sensitive to pro-oxidant challenges compared to individual antioxidant measurements Requires specialized equipment (e.g., gas chromatography) and precise sample preparation Experimental TOSC values must fall within a specific range (25–45) for accurate results	Fish liver homogenate, strawberry Assessing biological resistance to oxidative stress in marine invertebrates Evaluating pollutant-induced oxidative damage Monitoring the effects of environmental pollution on marine ecosystems Characterizing the TOSC of marine organisms from different environments Assessing the impact of chemical pollutants on marine organisms under field or laboratory conditions Monitoring oxidative stress in aquatic ecosystems Evaluating the effects of environmental stressors on cellular toxicity mechanisms	Magalhães et al., 2008; Regoli, 2000; Aruoma, 2003
Crocin bleaching assay	Simple and specific to crocin oxidation; an economical and sensitive method Simple and effective for aqueous samples Capable of distinguishing samples with high uric acid or bilirubin content	Limited to hydrophilic antioxidants; not suitable for samples with high bilirubin levels (> 3 mg/dL) Interference from compounds with similar absorbance (e.g., bilirubin, crocetin) Requires pure α-crocin for consistent results	Phenolic compounds and flavanones: Determination of antioxidant capacity in plasma, natural compounds, and plant extracts Useful in clinical laboratories for assessing antioxidant potential Can be adapted for lipophilic compounds with modifications	Magalhães et al., 2008; Bathaie et al., 2011
Trolox equivalence antioxidant capacity (TEAC)	Adaptable to high-throughput analysis Measures both hydrophilic and lipophilic antioxidants Simple to operate Reproducible Applicable to both hydrophilic and lipophilic antioxidants Can be used in multiple media Pre-formed ABTS• + is stable when stored in the dark	The ABTS• + radical is not biologically relevant. It measures both ABTS• + and ferrylmyoglobin radicals, which may complicate interpretation. Additionally, it requires calibration with Trolox	Suitable for both water-soluble and lipid-soluble antioxidants Forms the basis for more elaborate assays with additional time points, samples, and complex calculations Plant extracts, such as peaches, for the determination of antioxidant capacity in biological fluids Evaluation of both hydrophilic and lipophilic antioxidants	Aruoma, 2003; Silvestrini & Mancini, 2024; Pinchuk et al., 2012
TRAA (tocopheroxyl radical attenuating ability)	- Simple and rapid screening method - High selectivity for active compounds - Reproducible - No biological materials required	- Requires ESR equipment - Qualitative data only - Does not consider in vivo metabolites - No direct link to LDL lipid peroxidation	Screening potential LDL antioxidants Predicting anti-atherogenic activity of compounds Research on atherosclerosis	Aruoma, 2003; Witting et al., 1996
FC assay	Simple and Rapid: Easy to perform with fast results Cost-effective: Low-cost analysis method Reproducibility: Good reproducibility with a coefficient of variation (CV) of 1.14% Linearity: Excellent linearity with an R^2^ value of 0.99972 Sensitivity: Effective for analyzing a wide range of phenolic compounds Scientific Acceptance: Widely used and recognized in the scientific community	Non-Specific: Measures total reducing capacity, not just antioxidants Interference from Reducing Agents: Ascorbic acid or amino acids can affect the analysis Overestimation Risk: Non-phenolic reducing agents may cause overestimation of phenolic content Not Specific to Phenolic Compounds: Can react with other reducing agents Control Requirements: Needs careful management of pH, temperature, and reaction time	Tea, Wine, Fruit Juices, Beer: Common sources of phenolic compounds Quantification of Total Phenolic Content: Measuring phenolic content in foods like wine, olive oil, fruits, and vegetables Assessment of Antioxidant Capacity: Evaluating antioxidant properties in dietary studies Mediterranean Diet and Phenolic Intake: Research on the relationship between Mediterranean diet and phenolic consumption	Cano et al., 1998; Rumpf et al., 2023; Magalhães et al., 2008; Pérez et al., 2023
DMPD (N,N-dimethyl-p-phenylene diamine dihydrochloride) method	Fast and reproducible Measures plasma oxidative capacity with great accuracy Involves fewer steps and is less time-consuming compared to other methods Stable color development (stable for at least 30 min)	This method is not suitable for detecting antioxidant capacity in plasma due to high levels of Fe^3^⁺ It cannot be used for measuring the antioxidant capacity of hydrophobic compounds It is not suitable for samples containing iron due to interference Longer incubation times can lead to increased free radical production, which enhances color intensity over time Careful handling of reagents and plasma samples is required	Used to measure the TAC of biological samples (e.g., human plasma) and food and beverages (e.g., various wines) Measured plasma oxidative capacity during human aging Studied oxidative stress and its effects on aging Assessed the oxidant potential in biological samples	Dasgupta & Klein, 2014; Mehdi & Rizvi, 2013; Siddeeg et al., 2021
H_2_O_2_ scavenging activity assay	Simple and accurate colorimetric method Can measure H₂O₂ scavenging activity of bacterial and plant extracts High sensitivity due to UV–Vis detection	Ambiguous Results: Multiple pathways of inhibition can lead to unclear outcomes Interference: Phenolic compounds may interfere with UV absorption Reaction Conditions: Requires precise control of pH and temperature Limited Scope: Only measures H₂O₂ scavenging activity	Assessing the antioxidant activity of bacterial and plant extracts Evaluating the H₂O₂ scavenging potential in biological and environmental samples Studying mechanisms of oxidative stress	Chelliah, 2022; Haida & Hakiman, 2019
Conjugated diene assay	Simple method for detecting and quantifying hydroperoxides Applicable to a variety of matrices (dietary lipids, emulsions, biological matrices) Uses a classical approach with a recommended correction for improved accuracy	Requires solubilization of lipids in 2-propanol Dependent on the presence of conjugated diene structures Requires a nonoxidized blank sample for correction	Measures early lipid peroxidation products at 233 nm Analysis of lipid oxidation in dietary lipids Study of oil-in-water emulsions Investigation of biological matrices stabilized by proteins	Ribourg-Birault et al., 2024
Metal chealting assay	High sensitivity, wide pH tolerance, low interference from foreign ions	Limited to Fe^2^⁺ chelation, may not reflect in vivo biological activity	Evaluated the metal chelation capacity of various antioxidants, plant extracts, foods, and beverages (e.g., coffee and tea)	Danet, 2021a, b; Wong et al., 2014; Gulcin & Alwasel, 2022; Kotha et al., 2022; Chelliah & Oh, 2022; Moghrovyan et al., 2019

#### DNA damage assays-oxidative DNA damage assays

The DNA damage resulting from oxidative stress is a major research focus due to its profound implications for human health and disease. Oxidative stress arises from an imbalance between the production of ROS and the body's ability to detoxify these reactive intermediates. This imbalance can result in base modifications, abasic sites, and strand breaks (Gonzalez-Hunt et al., 2018; Katerji et al., 2019). Among the ROS, hydroxyl radicals are particularly reactive, capable of inducing a wide spectrum of DNA lesions, including sugar modifications, DNA–protein crosslinks, and strand breaks (Gonzalez-Hunt et al., 2018; Yu et al., 2018; Cadet et al., 2024; Dizdaroglu et al., 2020). Both external factors—such as air pollution, radiation, and lifestyle choices—and internal sources like mitochondrial respiration and inflammation contribute to this oxidative DNA damage (Gonzalez-Hunt et al., 2018; Katerji et al., 2019).

Despite significant advances, accurately measuring oxidative DNA damage remains challenging. This is especially true when distinguishing nuclear from mitochondrial DNA damage or quantifying basal lesion levels (Gonzalez-Hunt et al., 2018). A variety of assays have been developed, each with unique principles, strengths, and limitations. Below is an overview of these key techniques and their applications.

Madhujith et al. (2004) explored the protective effects of phenolic extracts from different bean varieties (white, brown, red, black) against peroxyl radical-induced DNA damage using supercoiled pBR322 plasmid DNA. They found that red, brown, and black bean hulls preserved DNA supercoiling against AAPH-induced oxidative stress, with antioxidant capacity attributed to phenolic compounds such as anthocyanins, procyanidins, and phenolic acids. Rahman et al. (2018) evaluated similar protective effects using camelina and sophia seed phenolics. Their study revealed potent inhibition of hydroxyl and peroxyl radical-induced DNA damage, with sophia’s esterified phenolics showing the highest activity (IC₅₀ = 1.14 mg/mL against ROO•). These findings suggest that phenolic-rich functional foods may mitigate ROS-mediated DNA damage.

Kunz et al. (2025) investigated DNA strand breaks induced by proton FLASH radiotherapy (ultra-high dose rate, UHDR) versus conventional dose rates (CDR) using supercoiled plasmids. Despite varying biological conditions, DNA strand break levels were independent of dose rate, suggesting that the FLASH effect's tissue-sparing benefits are not due to reduced DNA damage. Instead, other biomolecular targets such as lipids or proteins may mediate these protective effects.

The comet assay detects DNA strand breaks at the single-cell level, with DNA fragments forming a “tail” under electrophoresis. It is commonly used for detecting single-strand breaks (SSBs), double-strand breaks (DSBs), and alkali-labile sites (Gonzalez-Hunt et al., 2018; Møller et al., 2020; Collins et al., 2024). Enzyme-modified versions enhance detection of oxidatively damaged bases by incorporating lesion-specific enzymes. Despite low throughput and limited applicability to mitochondrial DNA, its single-cell resolution and adaptability make it a valuable biomonitoring tool (Collins, 2014).

PCR-based methods detect DNA damage by measuring the inhibition of polymerase amplification due to DNA lesions. These assays can quantify damage in both nuclear and mitochondrial DNA but do not distinguish lesion types and require high-quality samples (Gonzalez-Hunt et al., 2018). Chen et al. (2007) demonstrated how supercoiled DNA conformation affects real-time PCR quantification. By correlating amplification efficiency with conformational changes, their method provides a sensitive approach to study mitochondrial DNA integrity under oxidative stress.

Immunological assays such as ELISA and immunofluorescence detect specific lesions like 8-oxo-2′-deoxyguanosine (8-oxo-dG), a common oxidative stress marker (Gonzalez-Hunt et al., 2018; Katerji et al., 2019). While convenient, they often suffer from limited specificity and sensitivity, especially for detecting low levels of basal lesions. Nonetheless, they remain useful in tissue-based studies and immunohistochemical analyses.

LC–MS/MS is considered the gold standard for detecting specific oxidative base lesions, including 8-oxo-dG. Its high sensitivity and specificity are offset by challenges such as the requirement for large DNA amounts and the risk of artifactual oxidation during sample preparation (Gonzalez-Hunt et al., 2018). Nonetheless, its utility in cancer and aging studies makes it indispensable for precise lesion quantification.

Southern blotting detects DNA lesions by treating DNA with lesion-specific glycosylases followed by electrophoresis and hybridization (Gonzalez-Hunt et al., 2018). Although labor-intensive, this method minimizes artifactual oxidation and allows for specific lesion detection. Green and Sambrook (2021) reviewed key improvements, including more robust nylon membranes, improved transfer techniques, and advanced detection systems, which have modernized this classic technique for nucleic acid analysis.

Mitochondrial DNA damage is particularly difficult to assess due to its low abundance, susceptibility to isolation-induced artifacts, and the lack of methods capable of pinpointing lesion locations (Gonzalez-Hunt et al., 2018). Further advancements in PCR-based, immunoassay, and mass spectrometry techniques are needed to enhance specificity, sensitivity, and throughput.

Recent developments such as OxiDIP-seq, OG-seq, and Click-code-seq offer genome-wide detection of oxidative DNA lesions at high resolution (Poetsch, 2020). However, challenges remain with antibody specificity, DNA fragmentation artifacts, and background interference. These emerging methods emphasize the need for cross-validation and optimized protocols to minimize technical artifacts.

Accurately assessing oxidative DNA damage is crucial for elucidating its role in disease processes such as cancer, neurodegenerative, and cardiovascular disorders. While existing techniques provide a diverse toolkit, improving resolution, throughput, and specificity—especially for mitochondrial DNA-remains a priority. Technological innovations will be essential for overcoming current limitations and advancing our understanding of oxidative DNA damage in health and disease.

#### F2-isoprostane assays

Oxidative stress, characterized by an imbalance between the production of ROS and the body's antioxidant defenses, has been implicated in a wide range of diseases, including cardiovascular disorders, neurodegenerative diseases, and cancer (Milne et al., 2007). Among the various biomarkers used to assess oxidative stress, F2-isoprostanes (F2-IsoPs), a class of prostaglandin-like compounds, have emerged as some of the most specific and reliable indicators. Formed through the non-enzymatic, free radical-mediated peroxidation of arachidonic acid, F2-IsoPs are chemically stable and specific to lipid peroxidation processes, distinguishing them from earlier markers like TBARS and malondialdehyde (MDA) (Milne et al., 2007; van't Erve et al., 2017).

The biological relevance of F2-IsoPs is reinforced by their detection in various physiological and pathological states. Elevated levels have been linked to conditions such as atherosclerosis, Alzheimer’s disease, and pulmonary disorders, reflecting their role in oxidative injury (Milne et al., 2005). In atherosclerosis, F2-IsoP levels correlate with risk factors like hypercholesterolemia and diabetes; in Alzheimer’s disease, increased cerebrospinal fluid (CSF) F2-IsoPs reflect the severity of neurodegeneration. Similarly, in pulmonary diseases such as asthma and smoking-related pathologies, they indicate inflammation and oxidative damage.

Recent evidence highlights the clinical significance and complexity of F2-IsoP interpretation. A meta-analysis by van't Erve et al. (2017) assessed 8-iso-PGF2α across 50 human health conditions, revealing that diseases such as cystic fibrosis (g = 2.3), pulmonary arterial hypertension, and chronic renal insufficiency (g = 1.9) demonstrated stronger associations with oxidative stress than traditional cardiovascular risks like hypertension (g = 0.4) or metabolic syndrome (g = 0.5). These findings challenge conventional assumptions and underscore the need for refined approaches to F2-IsoP quantification and interpretation.

The formation, metabolism, and quantification of F2-IsoPs are inherently complex. While most F2-IsoPs result from non-enzymatic peroxidation, certain isomers may also arise through COX-mediated pathways, complicating their interpretation. Additionally, their metabolism involves enzymatic hydrolysis by platelet-activating factor acetylhydrolase (PAF-AH) and other pathways that vary across tissues, further impacting their levels in biological samples (Milne, 2017).

Analytical methods for quantifying F2-IsoPs include gas chromatography-mass spectrometry (GC–MS), liquid chromatography-mass spectrometry (LC–MS), and enzyme-linked immunosorbent assays (ELISAs) (Milne et al., 2007; Soffler et al., 2010). GC–MS, particularly in its negative ion chemical ionization mode (GC/NICI-MS), remains the gold standard due to its high specificity and ability to distinguish among multiple isomers. However, it requires complex sample preparation and access to specialized equipment. ELISAs offer simplicity and accessibility but suffer from limited specificity and cross-reactivity. Comparative studies have demonstrated poor agreement between ELISA and GC–MS measurements, reinforcing the importance of validated and standardized methodologies (Soffler et al., 2010).

In vitro studies offer valuable insights by allowing the controlled assessment of oxidative stress under defined experimental conditions. For instance, quantifying F2-IsoPs in cell cultures or tissue homogenates can help elucidate the oxidative response to various stimuli. Notably, other related biomarkers like isofurans (IsoFs), which are preferentially formed under high oxygen tensions, complement F2-IsoPs by providing additional information about oxygen-dependent lipid peroxidation (Milne et al., 2013).

In vivo quantification of F2-IsoPs in fluids such as plasma, urine, and exhaled breath condensate allows for non-invasive assessment of systemic oxidative stress. Urinary 15-F2t-IsoP (8-iso-PGF2α) is particularly valuable for clinical and epidemiological studies. Advanced methods like GC–MS/MS and improved extraction protocols (e.g., liquid–liquid extraction) have enhanced throughput, accuracy, and reproducibility for such applications (Briskey et al., 2014).

Despite these advances, challenges remain in fully translating F2-IsoPs into clinical practice. Lack of methodological standardization across laboratories, biological variability in isomer formation and clearance, and insufficient understanding of their mechanistic roles limit their current use in precision medicine. Future efforts should focus on developing standardized protocols, exploring the role of non-enzymatic versus COX-derived isomers, and improving accessibility through high-throughput, accurate, and less resource-intensive assays.

Conclusively, F2-IsoPs represent a robust and validated biomarker for oxidative stress assessment in both experimental and clinical contexts. Their utility in disease monitoring, environmental exposure assessment, and potentially guiding antioxidant therapies is promising. However, rigorous standardization and deeper mechanistic insights are essential for harnessing their full potential in clinical diagnostics and personalized medicine (Milne, 2017; van't Erve et al., 2017).

### Cellular antioxidant activity (CAA) assay: principles and applications

Cellular antioxidant assays are increasingly favored over traditional in vitro chemical models due to their ability to provide biologically relevant insights into oxidative stress responses within living cells. Unlike chemical assays such as DPPH and ABTS, cellular models—using cell lines like Caco-2, HepG2, or SH-SY5Y—consider critical factors such as compound uptake, metabolism, and intracellular redox signaling, thereby yielding more physiologically meaningful data. These models evaluate multiple antioxidant mechanisms, including ROS scavenging, antioxidant enzyme activation, and cytoprotective effects, bridging the methodological gap between chemical assays and complex in vivo studies (Meng et al., 2017). Redox reactions, involving electron transfer and changes in redox states, are fundamental to cellular metabolism, energy production, and signaling pathways. They regulate key processes such as cell proliferation and apoptosis through structural modifications of proteins (Meng et al., 2017). While ROS are natural byproducts of metabolism, their excessive accumulation disrupts redox homeostasis and induces oxidative stress, leading to protein and lipid damage as well as DNA mutations (Zhang et al., 2017a, b, c). Endogenous defense mechanisms—including enzymatic systems like SOD and CAT, and non-enzymatic antioxidants—play a central role in neutralizing ROS. However, external sources of antioxidants are often required when internal systems are overwhelmed (Lang et al., 2024). Despite the widespread use of chemical assays like DPPH and FRAP, their inability to mimic biological conditions has prompted a shift toward more biologically relevant techniques, such as the cellular antioxidant activity (CAA) assay (Loh & Lim, 2018a, b). Introduced by Wang and Joseph (1999), the CAA assay employs dichlorofluorescin diacetate (DCFH-DA), which is converted intracellularly to a fluorescent compound (DCF) upon oxidation by ROS, allowing quantification of intracellular oxidative stress. This method replaced fluorescence microscopy, offering improved reproducibility and precision (Meng et al., 2017). Later refinements by Wolfe and Liu (2008), such as the use of HepG2 liver cells and the peroxyl radical generator ABAP, enhanced its physiological relevance by integrating parameters like antioxidant uptake and metabolism.

**Fig. 1:**
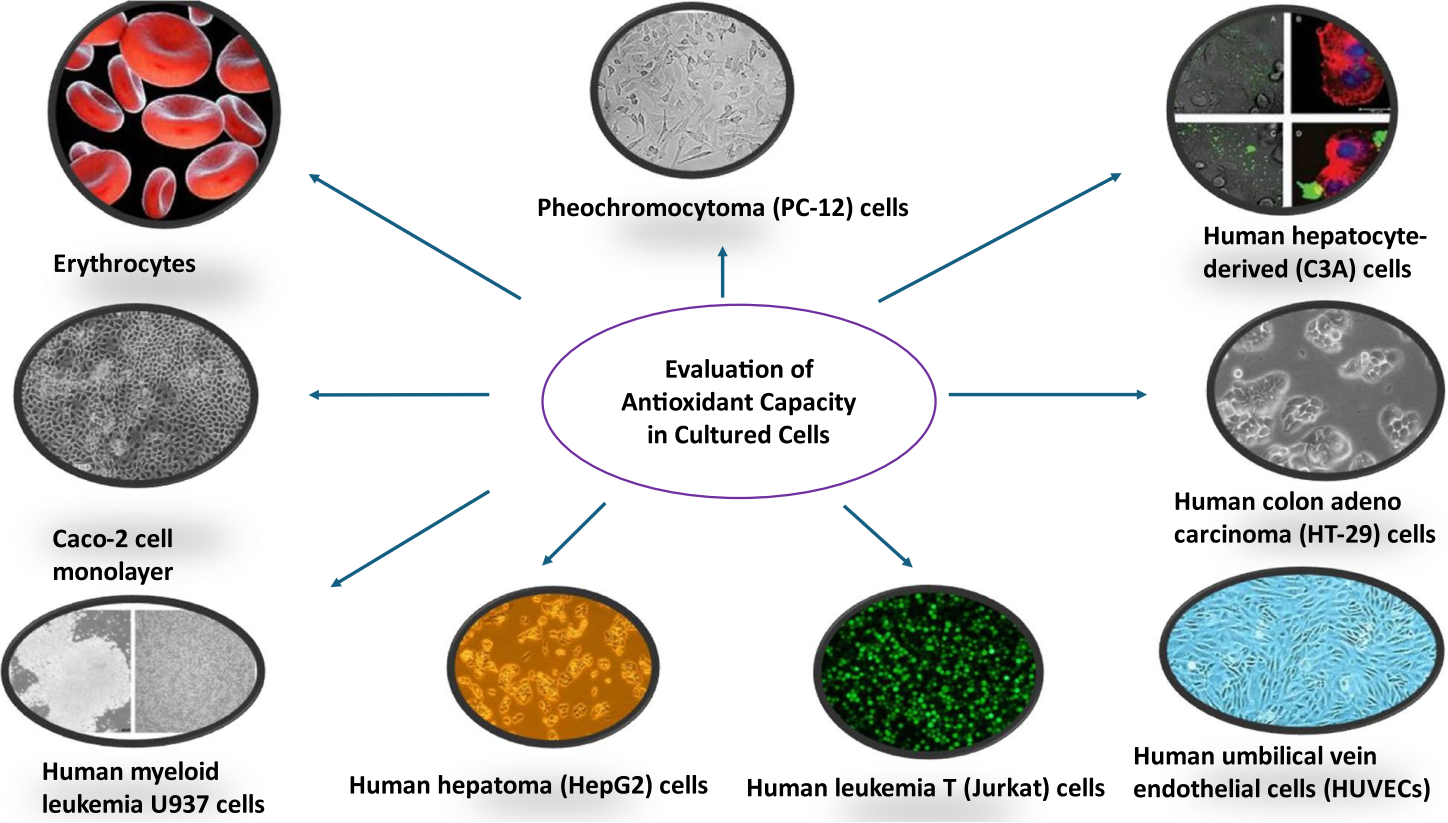
Different cell culture models used in the antioxidant assays

Figure 1 illustrates various cell culture models used in antioxidant activity determinations, highlighting their role in bridging the gap between in vitro chemical and in vivo assays. While the CAA assay offers enhanced biological relevance, it is not without limitations. It does not fully replicate systemic processes such as digestion, absorption, or inter-organ interactions (Lang et al., 2024). For example, gastrointestinal digestion can significantly alter antioxidant bioavailability and efficacy—an aspect not captured by CAA assays (Arouna et al., 2020). Furthermore, these assays often demand specialized instrumentation and technical expertise, restricting their broader application and scalability (Danet, 2021a, b). Nevertheless, the CAA assay is widely employed in food science, pharmacology, and nutrition research to evaluate the antioxidant potential of dietary supplements, functional foods, and phytochemicals (Meng et al., 2017). For instance, studies have used this assay to assess the antioxidant properties of polyphenols in fruits and vegetables, while oxidative hemolysis inhibition assays have evaluated antioxidant protection in red blood cells (RBCs) (Loh & Lim, 2018a, b; Arouna et al., 2020). Table 6 provides a summary of key CAA methodologies, biological models, and oxidative stress indicators, underscoring the assay's comparative advantages over traditional chemical methods (Engelbrecht et al., 2023). A noteworthy variant, the CAA-RBC assay, investigates antioxidant activity in erythrocytes under oxidative stress induced by AAPH. RBCs are especially suitable for such assays due to their high exposure to ROS and susceptibility to lipid peroxidation (Zhang et al., 2017a, b, c). For example, Frassinetti et al. (2018) reported that hemp seed extracts (CAA = 82 ± 4) were significantly more effective than sprout extracts (CAA = 34 ± 2 to 42 ± 3.5) in protecting RBCs from oxidative hemolysis, although Trolox (CAA = 94 ± 2) remained the most effective standard. Beyond HepG2, other cell lines are also extensively used in antioxidant research. Caco-2 cells, which mimic intestinal absorption, are ideal for studying the bioavailability of phenolic compounds like quercetin and (+)-catechin (Kellett et al., 2018). Liu et al. (2019) demonstrated that sulforaphane exerted dose-dependent effects in HepG2 cells, enhancing antioxidant defenses at lower doses (≤ 5 μM) but inducing DNA damage at higher concentrations (≥ 20 μM). PC-12 cells are commonly used for evaluating neuroprotective effects of antioxidants (Jang & Surh, 2003), while HT-29 cells are suited for studying antioxidant peptides (Guo et al., 2014a). Jurkat cells are used to investigate oxidative DNA damage (Céliz et al., 2013a), and the oxidative hemolysis inhibition assay remains a direct method to assess membrane protection (Arouna et al., 2020). In an interesting application, Yun et al. (2016) evaluated the antioxidant effects of cultured wild ginseng root extracts (cWGRE) on boar sperm under oxidative stress induced by xanthine and xanthine oxidase (X-XO). They found that cWGRE significantly reduced ROS levels and improved sperm motility (*P* < 0.05) compared to controls. Moreover, co-treatment with cWGRE and CAT showed a synergistic effect, suggesting that cWGRE might enhance male fertility through antioxidant mechanisms.

**Table 6:** Overview of ex vivo and cellular antioxidant activity (CAA) assay models

Method	Mechanism	Biological model	oxidative stress inducer	Measured Antioxidant Parameters	End-product determination	References
GST assay	Conjugates GSH to xenobiotic substrates for detoxification	Liver tissue or blood homogenates	Xenobiotics or ROS-generating agents	GST enzyme activity	Spectrophotometric measurement of GSH conjugation	Hejazi et al., 2018; Siddeeg et al., 2021
SOD assay	Converts superoxide radicals to H₂O₂ and O₂	Liver tissue homogenates	H₂O₂ or ROS-generating agents	SOD enzyme activity	Spectrophotometric measurement of superoxide levels	Hejazi et al., 2018
CAT assay	Decomposes H₂O₂ into water and oxygen	Liver tissue homogenates	H₂O₂	CAT enzyme activity	Spectrophotometric measurement of H₂O₂ degradation	Hejazi et al., 2018
GPx assay	Reduces H₂O₂ or organic hydroperoxides using GSH	Liver tissue homogenates	H₂O₂ or organic hydroperoxides	GPx activity	Spectrophotometric GSH consumption or NADPH oxidation	Hejazi et al., 2018; Ndile et al., 2025
GR assay	Converts GSSG to GSH using NADPH	Liver tissue homogenates	H₂O₂ or ROS-generating agents	GR activity	Spectrophotometric NADPH oxidation	Hejazi et al., 2018
Lipid peroxidation assay (TBARS)	Free radicals cause lipid and protein oxidation; MDA indicates damage	Liver tissue, rat brain homogenate, LDL	Fenton’s reagent (FeSO₄ + H₂O₂)	MDA, protein carbonyls	MDA-TBA complex at 532 nm	Hejazi et al., 2018; Pai Kotebagilu et al., 2015
Oxidation of low-density lipoprotein (LDL) assay	Measures oxidation of LDL induced by ROS/RNS, using cupric sulfate as an initiator	LDL particles	CuSO₄	Lag phase in kinetic profile (endogenous antioxidants like coenzyme Q, vitamin E)	Spectrophotometry (diene conjugates at 234 nm) or chemiluminescence (emitted radiation)	Danet, 2021a, b
	oxLDL → ROS → endothelial dysfunction, mitochondrial collapse	HUVECs, HepG2	oxLDL (50–200 μg/mL)	Electrophoretic mobility of oxLDL, ApoB fragmentation, caspase-3 activation	Mitochondrial ΔΨm, cytochrome C release, adhesion molecule expression	Chen et al., 2015; Lin et al., 2014; Cominacini et al., 2000
Ex vivo AAPH-induced hemolysis assay	Peroxyl radical scavenging via AAPH inhibition; replaces CAA data	Rat erythrocytes	AAPH (200 mM)	Hemolysis inhibition %, MDA, GSH, enzyme activities	Spectrophotometry, IC₅₀ values	Kpemissi et al., 2019; Banerjee et al., 2008a
Ex vivo DCFH-DA assay	Antioxidant protection against t-BuOOH-induced oxidative stress	Human fibroblast (WS-1) cells	t-BuOOH (200 μM)	Fluorescence intensity	IC₅₀ values (µg/mL)	Karker et al., 2016
Antimutagenesis assay	Mitotic gene conversion and point mutations in yeast	*S. cerevisiae*	H₂O₂ (13 mM)	Mutation inhibition	GC and PM frequency reduction	Frassinetti et al., 2018
Ex vivo vessel function assay	Endothelial protection and eNOS/NO modulation	Mouse aorta, **(**Human Aortic Endothelial Cells) HAECs	HOCl (100 µM)	NO production, Hmox-1, cell viability	eNOS dimerization, MTS assay	Jiang et al., 2016
Ex vivo tape stripping-DPPH assay	DPPH radical scavenging in human skin	Human forearm skin	Solar simulator (UV exposure)	% Free radical scavenging	Absorbance at 515 nm	Peres et al., 2018
Respiratory burst assay	Measures inhibition of PMA-induced superoxide in blood	Human whole blood	Phorbol myristate acetate (PMA) to activate NADPH oxidase and induce superoxide anion production	Superoxide anion inhibition (%) and total phenolic content (TPC)	Percentage of superoxide anion inhibition, calculated from the area under the chemiluminescence curve	Matute et al., 2021
H₂O₂-induced stress	Antioxidant enzyme modulation	Rat erythrocytes	H₂O₂	SOD/CAT/GPx activity, MDA, GSH	Spectrophotometry	Çimen, 2008
Ex vivo assay to measure inhibition of FeCl₂-ascorbic acid-induced lipid peroxidation in kidney homogenate	Inhibition of lipid peroxidation by scavenging hydroxyl radicals	*Rat kidney homogenate*	FeCl₂-ascorbic acid	MDA levels as a marker of lipid peroxidation	MDA concentration (nmol/g tissue)	Kpemissi et al., 2019
Oxidative stress cell model	Simulates oxidative stress conditions by inducing ROS production using stressors like H2O2, t-BHP, t-BuOOH, or AAPH. Measures ROS levels, oxidative damage markers, and antioxidant enzyme activity	*Red blood cells, HepG2, Caco-2, PC-12 cells*	H2O2, t-BHP, t-BuOOH, AAPH	ROS levels, MDA, GSH, antioxidant enzymes (SOD, CAT, GPx, HO-1, PRDX4, GR, GST), lipid peroxidation, DNA/protein damage	Biochemical markers (e.g., MDA, GSH, enzyme activity)	Lang et al., 2024
2,2'-Azobis(2-amidinopropane) dihydrochloride (AAPH) -induced hemolysis	Inhibition of lipid peroxidation and ROS scavenging	Human erythrocytes	AAPH	Hemolysis inhibition, MDA levels, GSH content, SOD/CAT/GPx activities	Hemolysis %, TBARS (MDA), GSH levels, enzyme activities	Banerjee et al., 2008a
Cu^2^⁺-induced oxidation	Prevention of hemoglobin oxidation and membrane damage	Human erythrocytes	CuCl₂/CuSO₄	Hemoglobin oxidation, hemolysis, MDA formation, antioxidant enzyme activities (SOD, CAT, GPx)	Hemoglobin oxidation rate, hemolysis %, MDA levels	Liao et al., 2014a
H₂O₂-induced stress	ROS scavenging and enzyme activity modulation	Rat erythrocytes	H₂O₂	GSH depletion, SOD/CAT/GPx activity, lipid peroxidation (MDA)	GSH levels, enzyme activities, MDA content	Çimen, 2008
AAPH & Cu^2^⁺-induced stress	Synergistic antioxidant protection	Human erythrocytes	AAPH + CuSO₄	Hemolysis inhibition, LDL oxidation, cholesterol degradation	Hemolysis %, LDL oxidation (cholesterol esters)	Hseu et al., 2014a, b
Peroxyl radical scavenging	Direct radical quenching and intracellular protection	Human erythrocytes	AAPH	Intracellular GSH oxidation, peroxyl radical scavenging	GSH oxidation rate, radical scavenging capacity	Banerjee et al., 2008a
Fe^2^⁺-ascorbate system	Prevention of hydroxyl radical-induced lipid peroxidation	Human erythrocytes	Fe^2^⁺/Ascorbate	TBARS (MDA), hemolysis %, protein carbonylation	MDA levels, hemolysis %, protein carbonyls	Duchnowicz et al., 2005a
Hypochlorous acid (HOCl) stress	Protection against chlorination and oxidation	Human erythrocytes	HOCl	GSH depletion, hemoglobin oxidation, membrane protein damage	GSH levels, hemoglobin oxidation, protein cross-linking	Bors et al., 2011
Curcumin protection	Inhibition of lipid peroxidation and apoptosis	Chicken erythrocytes	Heat stress	Apoptosis (Annexin V), hemolysis %, SOD activity, MDA levels	Apoptosis rate, hemolysis %, SOD activity, MDA	Zhang et al., 2014a
Polyphenol protection	Preservation of antioxidant enzymes and ROS suppression	Human erythrocytes & HepG2	AAPH, Cu^2^⁺	ROS formation, TBARS, SOD/CAT/GPx activities	ROS levels, TBARS, enzyme activities	Liu & Huang, 2015a, b
5-HMF (furan derivative)	Reduction of ROS and lipid peroxidation	Erythrocytes & A375 cells	Not specified	ROS levels, MDA content, SOD/CAT/GPx activities	ROS, MDA, enzyme activities	Zhao et al., 2013a
Pleurotus abalonus extract	Hemolysis inhibition via membrane stabilization	Kunming mice erythrocytes	Not specified	Hemolysis inhibition	Hemolysis %	Zhang et al., 2013a
HepG2 cell transfection	Cytosolic/mitochondrial CAT overexpression for oxidant protection	Human hepatoma (HepG2) cells	H₂O₂, AAPH	CAT, SOD, GPx activities	ROS content, MDA levels, protein expression	Bai et al., 1999; Tolosa et al., 2013; Martin et al., 2008; Ham et al., 2015
Caco-2 monolayer model	Transport/absorption mechanisms in gut-like structure	Human colon intestinal carcinoma (Caco-2)	H₂O₂, AAPH	SOD, GPx, CAT activities	ROS generation, lipid peroxidation	Fogh et al., 1977; O'Sullivan et al., 2013; Yee, 1997; You et al., 2016
PC-12 differentiation assay	Neuroprotection against β-amyloid/oxLDL-induced damage	Rat pheochromocytoma (PC-12) cells	oxLDL, AAPH	SOD, CAT, GPx activities	Apoptosis, oxidative damage markers	Zhou et al., 2006; Goldstein et al., 2012; Yao et al., 2014; Jang & Surh, 2003
HT-29 metabolic stress assay	Antioxidant peptide protection under glucose/glutamine deprivation	Human colon adenocarcinoma (HT-29) cells	H₂O₂ (1 mM)	SOD, CAT, GPx activities	Neoplasia/therapeutic responses	Lesuffleur et al., 1990; Guo et al.,^2014^
U937 leukemic cell model	Immunomodulatory effects on ROS production	Human myeloid leukemia (U937) cells	AAPH, H₂O₂	Superoxide/H₂O₂ production	Lipid peroxidation, antioxidant enzyme activity	Harris & Ralph,^1985^, Sestili et al., 2002a; McCarthy et al.,^2012^
C3A hepatocyte assay	Evaluation of oyster-derived DHMBA antioxidant effects	Human hepatocyte-derived (C3A) cells	Not specified	AST/ALT expression	Lipid peroxidation, antioxidant enzyme activity	Fuda et al., 2015, Watanabe et al., 2012a, b
Jurkat T-cell oxidation assay	Lipid/DNA damage under oxidative stress	Human leukemia T (Jurkat) cells	AAPH	Lipid peroxidation, DNA damage	Membrane marker changes, lymphocyte-like responses	Koníková et al., 1992; Erba et al., 1999; Céliz et al., 2013a
HUVEC endothelial dysfunction	oxLDL-induced ROS generation and NO synthase modulation	Human umbilical vein endothelial cells	oxLDL	ROS, caspase-3 activation, mitochondrial destabilization	Endothelial dysfunction markers	Kuo et al., 2009; Chen et al., 2015; Mochalski et al., 2015
AAPH radical induction	Peroxy radical generation for lipid oxidation (reactions 1–5)	Multiple cell models	AAPH	Conjugated dienes (234 nm), SOD/GPx/CAT activities	MDA levels, ROS content, antioxidant enzyme expression	Niki, 1990; Fantozzi et al., 1998; Peyrat-Maillard et al., 2003
Antioxidant pretreatment assays	Hydrogen donation to stabilize radicals (e.g., curcumin, peptides, polyphenols)	Erythrocytes, fibroblasts, HepG2, etc	AAPH (10–200 mM)	SOD/CAT/GPx activities, protein expression	Radical-scavenging efficiency, enzyme activity normalization	Gokila Vani et al., 2013; Zhang et al., 2014a; Alvarez-Suarez et al., 2016
Cytochrome P-450/Hb metabolism	Free radical intermediates → lipid peroxidation, GSH depletion, Ca^2^⁺ dyshomeostasis	Rat hepatocytes, HepG2, Caco-2, U937	t-BHP (100–1000 μM)	GSH/GSSG ratio, MDA, SOD/GPx/CAT/GR activities, DNA damage	Mitochondrial depolarization, cytosolic enzyme leakage (LDH, ALT), DNA single-strand breaks	Rush et al., 1985; Tseng et al., 1996; Fernandes et al., 2003
Univalent reduction of O₂	H₂O₂ → HO• radicals; membrane diffusion → apoptosis/inflammation	HepG2, Caco-2, PC-12, U937, HUVECs	H₂O₂ (0.2–1 mM)	SOD/CAT/GPx/GR activities, GSH levels, IL-1β/IL-6/IL-8/TNF-α	DNA damage (OH•-induced), cytokine secretion, cell viability	Zhang et al., 2016; Peng et al., 2016; Bak et al., 2014

Fluorescence-based assays like the CAA offer dynamic, sensitive tools for measuring intracellular antioxidant capacity (Loh & Lim, 2018a, b). However, interpreting these results requires careful attention to probe specificity and cellular uptake (Lang et al., 2024). Complementary methods, such as the comet assay, are useful for assessing the DNA-protective effects of antioxidants. Frassinetti et al. (2018) demonstrated through comet assay that hemp extracts significantly reduced H₂O₂-induced DNA damage in yeast cells, highlighting the assay's potential in antioxidant research targeting genetic material protection (Lee et al., 2017).

## Ex-vivo antioxidant assays

The ex vivo antioxidant methods are increasingly favored over in vitro and cellular assays due to their ability to preserve native tissue physiology, including cell–cell interactions and metabolic processes, while circumventing the ethical and logistical complexities associated with in vivo studies. Unlike chemical assays (e.g., DPPH) or cell-based models (e.g., Caco-2), ex vivo systems—such as isolated organs, tissues, or blood samples—better replicate real biological responses to oxidative stress, effectively bridging the gap between simplified in vitro tests and complex animal models. This makes ex vivo models particularly suitable for translational antioxidant research, offering physiologically relevant insights into compound efficacy and bioavailability (Arouna et al., 2020; Sallam et al., 2022). For example, human red blood cell (RBC) assays have effectively demonstrated antioxidant protection against oxidative hemolysis while also considering metabolic influences, such as gut microbiota-mediated transformations that can alter antioxidant profiles (Blasa et al., 2011). These models are especially valuable for studying isolated tissues, cells, or fluids to elucidate oxidative stress mechanisms and evaluate therapeutic potential.

Ex vivo systems also enable more accurate assessment of enzymatic antioxidant defenses, including SOD, CAT, and GPx, which work synergistically to neutralize ROS and regulate redox homeostasis. SOD converts superoxide radicals into hydrogen peroxide, which is subsequently detoxified by CAT and GPx (Weydert & Cullen, 2010). Measuring these enzyme activities in biological samples adds a mechanistic dimension often missing in traditional in vitro assays. Common ex vivo techniques—such as lipid peroxidation assays and ROS/RNS detection—provide biologically relevant data on how antioxidants mitigate oxidative damage. For instance, Rivera -Yañez et al. (2018) used the TAC assay to evaluate the peptide PIC1, revealing its efficacy via single electron transfer (SET) and hydrogen atom transfer (HAT) mechanisms. These results highlight the need to understand antioxidant structural features, such as those found in bioactive peptides, that influence their mode of action and potency (Zou et al., 2016).

The increasing complexity of antioxidant–ROS interactions has driven the evolution of diverse ex vivo techniques. High-sensitivity chemiluminescence assays, for instance, allow the evaluation of ROS scavenging and neuroprotective effects in biological systems (Chang et al., 2013). Peres et al. (2018) applied an ex vivo DPPH assay on human forearm skin exposed to a solar simulator, using real human tissue to evaluate topical antioxidant formulations. Their findings revealed no significant differences between test and control groups, underscoring the need for methodological optimization to capture subtle bioactive effects. In a complementary study, Karker et al. (2016) used WS-1 human skin fibroblasts to assess the antioxidant activity of *R. vermiculata* extracts, with methanol (IC₅₀ = 3.2 μg/mL) and water (IC₅₀ = 6.3 μg/mL) extracts outperforming those derived from non-polar solvents, likely due to their richer phenolic profiles.

Overall, ex vivo assays offer critical advantages over in vitro and even some in vivo models. They retain essential biological interactions—such as cellular uptake, metabolism, and enzymatic activity—delivering more translatable and clinically relevant data (Arouna et al., 2020; Sallam et al., 2022). RBC-based assays, for example, provide insights into both antioxidant protection and systemic bioavailability, making them indispensable tools for screening dietary and therapeutic antioxidants. Future research efforts should focus on refining these models to more closely emulate human physiology. Integrating ex vivo data with in vitro and in vivo findings is vital for a holistic understanding of antioxidant efficacy. Studies such as those by Alonso et al. (2016) using lipid peroxidation in human skin and by Dobrecky et al. (2020) employing TBARS in plant extracts, underscore the translational potential of ex vivo methods. Discrepancies observed between in vitro and in vivo outcomes can often be clarified through ex vivo evaluations, which better account for real biological complexity, including metabolic and bioavailability factors (Ky & Teissèdre, 2015). For instance, George et al. (2014) demonstrated that *Polygonum minus* extracts showed stronger antioxidant activity in ex vivo models than in vitro, reinforcing the importance of biological context in antioxidant research.

## Enzyme-based antioxidant assays (in-vitro and ex-vivo)

### Superoxide dismutase assays

The SOD (enzyme code EC1.15.1.1) family first isolated from erythrocytes, brain, and liver, plays a critical antioxidant role by catalyzing the dismutation of superoxide (O₂^•⁻^) into H₂O₂ and O₂. Three major SOD isoforms exist with distinct localizations and functions: (1) Cu,Zn-SOD (SOD1), a cyanide-sensitive copper-zinc enzyme predominantly found in the cytosol and liver nuclei; (2) Mn-SOD (SOD2), a mitochondrial matrix-localized, cyanide-resistant, manganese-dependent enzyme essential for neutralizing ROS generated by the respiratory chain; and (3) ecSOD (SOD3), the extracellular isoform found in plasma and the extracellular matrix. In liver tissue, Cu,Zn-SOD contributes the most to total SOD activity, while Mn-SOD is critical for mitochondrial protection. Dysregulated SOD activity is associated with various pathologies, including aging, cancer, diabetes, and liver diseases. Paradoxically, excessive SOD activity can exacerbate oxidative stress through the overproduction of H₂O₂ (Campos-Shimada et al., 2018; Zheng et al., 2023).

Weydert and Cullen (2010) measured SOD activity in cell lines (20–90 U/mg protein) and tissues using two complementary approaches: quantitative biochemical assays (requiring 10 × more protein) and qualitative native gels. Native PAGE revealed distinct bands corresponding to Mn-SOD (mitochondrial) and Cu,Zn-SOD (cytosolic), with adenoviral transfection increasing activity up to 30-fold. Immunofluorescence in MCF10A and MIA PaCa-2 cells confirmed mitochondrial localization of Mn-SOD, while pancreatic cancer tissues showed a marked loss of Mn-SOD compared to normal pancreatic tissue, emphasizing its relevance in disease.

The xanthine oxidase-cytochrome c system, first developed by McCord and Fridovich (1969), utilizes xanthine oxidase to generate superoxide radicals (O₂^•⁻^) while cytochrome c serves as an indicating scavenger. These techniques serve the dual purpose of not only quantifying SOD activity but also distinguishing between different SOD types, particularly Cu/Zn-SOD versus Mn/Fe-SOD, through the strategic use of specific inhibitors like cyanide. Similarly, the xanthine oxidase-sulfite system employs sulfite as the O₂^•⁻^ scavenger (Goldstein et al., 1988; Tyler, 1975). Alternative approaches include the riboflavin-dianisidine assay, where aerobic riboflavin mixtures are coupled with dianisidine (Misra & Fridovich, 1977), and the epinephrine autoxidation method that measures SOD's ability to inhibit epinephrine oxidation (Misra & Fridovich, 1972). The 6-hydroxydopamine assay uniquely uses 6-hydroxydopamine as both the O₂⁻ source and scavenger (Cohen et al., 1974), while the pyrogallol autoxidation method relies on pyrogallol as both generator and indicator of superoxide radicals (Al-Gubory, 2008; Marklund & Marklund, 1974). These assays share a common principle of coupling O₂^•⁻^ generation (enzymatic or photochemical) with specific scavengers, quantifying SOD activity through spectrophotometric detection of scavenger oxidation inhibition. Each technique offers unique advantages for studying SOD kinetics and inhibition properties under controlled laboratory conditions. For rapid qualitative analysis, the NBT-PAGE staining method provides efficient isoform detection in cell-free systems using riboflavin/TEMED-generated superoxide, serving as a valuable preliminary screening tool despite being non-quantitative (Flohé & Ötting, 1984). Together, these methods enable comprehensive SOD characterization from initial expression profiling to precise activity measurement, supporting research into oxidative stress-related diseases (Campos-Shimada et al., 2018). Each technique offers unique advantages for studying SOD kinetics and inhibition properties under controlled laboratory conditions, allowing researchers to select the most appropriate method based on their specific experimental needs.

The differential SOD activity assay introduced by Campos-Shimada et al. (2018) offers an optimized ex vivo approach for simultaneously measuring cytosolic Cu,ZnSOD and mitochondrial MnSOD activities from a single liver sample. By combining differential fractionation (sequential centrifugation at 7,080 × g for mitochondria and 15,000–105,000 × g for cytosol) with selective cyanide inhibition (2 mM KCN for Cu,ZnSOD specificity) and pyrogallol autoxidation detection (420 nm, pH 8.2), this method overcomes limitations of traditional in vitro assays. It avoids interference from endogenous antioxidants and cytochrome c oxidase while preserving physiological enzyme conditions. Sonication enhances MnSOD sensitivity sevenfold, enabling compartment-specific oxidative stress analysis (e.g., cancer progression) with minimal animal use.

Peskin and Winterbourn (2017) developed a WST-1-based SOD ex vivo assay, improving upon earlier methods by using xanthine oxidase to generate superoxide radicals that reduce WST-1 to a soluble formazan (measured at 438 nm). Key optimizations include CAT addition (prevents H₂O₂ interference), standardized xanthine oxidase activity, and 96-well plate compatibility for high-throughput analysis. KCN selectively inhibits Cu,ZnSOD, allowing isoform differentiation. The assay shows high reproducibility (~ 10% inter-assay variability) and sensitivity (50% inhibition with 2 ng Cu,ZnSOD/well). Unlike NBT-based methods, it avoids insoluble products, offering a robust, quantitative tool for antioxidant assessment. Assady et al., (2011a,) compared SOD activity in Fasciola parasites (*F. hepatica, F. gigantica*) and sheep liver (healthy vs. infected) using a xanthine/xanthine oxidase-based RANSOD kit. Infected liver exhibited significantly higher SOD activity (1.43 U/mg) than healthy tissue (0.51 U/mg), indicating an antioxidant response to parasitic oxidative stress. Parasite SOD levels were similar (0.57–0.58 U/mg), with SDS-PAGE revealing a conserved 60 kDa band. The findings suggest distinct host-parasite redox interactions, with elevated liver SOD reflecting defense against parasitic infection.

The study by Engelbrecht et al. (2023) describes a cost-effective SOD assay based on the inhibition of pyrogallol autoxidation, in which SOD activity is determined by its ability to neutralize superoxide radicals (O₂•⁻). Unlike traditional spectrophotometric methods that require individual cuvette measurements, this optimized protocol utilizes a 96-well microplate format, enabling high-throughput analysis of up to 32 samples in triplicate. The assay is particularly valuable for laboratories in resource-limited settings, as it eliminates the need for expensive commercial kits while maintaining accuracy. By measuring absorbance at 560 nm and normalizing the results to protein content (ng SOD/mg protein), the method offers a reliable assessment of oxidative stress in cell cultures exposed to environmental contaminants, as demonstrated in HuTu-80 and H4IIE-luc cell lines.

An inhibition of superoxide anion production assay using the chemiluminescence method, conducted by Matute et al. (2021), evaluated the antioxidant capacity of fruit and vegetable juices by quantifying their ability to suppress phorbol myristate acetate (PMA)-induced superoxide radical anion (O₂^•⁻^) generation in whole blood. The test involves incubating blood samples with lucigenin (a chemiluminescent probe) and diluted juices, followed by PMA stimulation to activate NADPH oxidase (NOX2) in neutrophils. The emitted chemiluminescence, which is proportional to O₂^•⁻^ levels, is recorded over 30 min, and the percentage of inhibition is calculated by comparing results with and without juice treatment. This ex vivo method provides rapid (30-min), sensitive, and biologically relevant data, showing strong correlations with phenolic content and superior performance compared to traditional assays such as ORAC. However, it requires fresh blood samples and does not account for in vivo metabolic factors.

Traditional SOD assays typically measure enzyme activity in tissue samples using spectrophotometric cuvette-based methods. However, Engelbrecht et al. (2023) developed a cost-effective, high-throughput alternative for mammalian cell cultures by adapting the assay to a 96-well microplate format. This method allows simultaneous measurement of SOD content in multiple samples (up to 32 per plate) using standard laboratory equipment, making it accessible for research labs in resource-limited settings. The assay utilizes cell culture supernatants, enabling parallel analysis of protein concentration and SOD levels, providing a practical tool for studying oxidative stress responses in vitro.

Immunological assays such as the electroimmunoassay (EIA) specifically quantify SOD protein levels through antibody-antigen binding, independent of the enzyme's catalytic activity. This makes EIAs particularly useful for detecting inactive or denatured forms of SOD in clinical samples and aging-related studies. Unlike activity-based assays, EIAs offer direct measurement of SOD concentration with high specificity, unaffected by variations in enzymatic function. They are especially valuable in correlating protein expression with disease biomarkers when enzymatic activity may be compromised, thereby providing complementary data to functional assays. Their antibody-based detection ensures reliable quantification even in complex biological matrices like serum or tissue extracts (Flohé & Ötting, 1984).

Building on this principle, Santharaman et al. (2016) developed a label-free electrochemical immunosensor to detect SOD1, a key oxidative stress marker associated with neurodegenerative and cardiovascular diseases. The sensor employs a gold nanoparticle–polypyrrole nanocomposite on a screen-printed carbon electrode, functionalized with anti-SOD1 antibodies to ensure high sensitivity and specificity. By leveraging SOD1’s nitrite oxidase activity, the sensor achieves a broad linear detection range (0.5 nM to 5 μM), a low detection limit (0.5 nM), and high sensitivity. It was successfully applied to measure SOD1 levels in human epidermal keratinocytes, demonstrating strong correlation with Western blot results. This cost-effective and rapid platform, which eliminates the need for complex labeling procedures, represents a promising tool for point-of-care diagnostics in oxidative stress-related disorders.

### CAT assay

CAT is a crucial antioxidant enzyme that protects cells against oxidative stress by decomposing hydrogen peroxide into water and oxygen. The CUPRAC-CAT method introduced by Hadwan et al. (2024) offers a robust and reliable approach for measuring CAT activity in biological samples. This method involves pre-incubation of enzymatic components, followed by the addition of the Cu(II)-neocuproine reagent. Remaining substrates reduce Cu(II) to Cu(I), forming a colored complex measurable at 450 nm. Since CAT activity is inversely proportional to the absorbance of this complex, the method enables accurate quantification. Optimized using response surface methodology and validated against the peroxovanadate standard through Bland–Altman analysis, the method demonstrated a strong correlation (*r* = 0.99) with conventional assays. Its sensitivity, reproducibility, and simplicity make it particularly suitable for evaluating CAT activity in liver tissues and for monitoring oxidative stress or bacterial contamination in food safety research.

The improved CAT activity measurement method developed by Hadwan et al. (2024) introduces a simple, sensitive, and safe spectrophotometric approach for measuring CAT activity in biological samples. The assay involves incubating the sample with H₂O₂, followed by stopping the reaction with ferrous ammonium sulfate (FAS) and sulfosalicylic acid (SSA), which form a maroon-colored ferrisulfosalicylate complex (absorbance at 490 nm) proportional to the residual H₂O₂ and inversely related to CAT activity. This method eliminates the use of toxic reagents (unlike the traditional ferrithiocyanate method), exhibits high sensitivity (limit of detection: 0.022 U/mL), and is resistant to interference from biomolecules. It can be performed in cuvettes or microplates, making it suitable for clinical, research, and environmental applications, including urinary infection detection, oxidative stress studies, and bacterial CAT analysis. Validation studies showed a strong correlation (*r* = 0.99) with standard methods, confirming the method’s reliability and precision (Kadhum & Hadwan, 2021).

The study by Kadhum and Hadwan (2021) presents a simple and precise spectrophotometric method for measuring CAT activity in biological samples. The assay involves incubating CAT with hydrogen peroxide (H₂O₂) in a phosphate buffer (pH 7.4), followed by the addition of a vanadate-pyridine-2,6-dicarboxylic acid solution to stop the reaction. The residual H₂O₂ forms a stable orange-colored oxo-peroxo-vanadate complex (OPDV), measured at 435 nm. The method was optimized using Box-Behnken design (BBD) and validated against the carbonato-cobaltate reference method, showing a high correlation (*r* = 0.9968). This approach is cost-effective, rapid, and avoids interference from biomolecules, making it suitable for both research and clinical applications.

Another methodology introduced by Hadwan (2018) presents a simple, precise, and sensitive spectrophotometric method for measuring CAT activity in biological tissues. The assay involves incubating samples with hydrogen peroxide, followed by reaction with a cobalt-bicarbonate reagent to form a stable carbonato-cobaltate (III) complex, which absorbs at 440 nm. CAT activity is proportional to hydrogen peroxide decomposition, and the method avoids interference from common biological compounds. Results showed a high correlation with the dichromate method (*r* = 0.9950), with low within-run (2.96%) and between-run (3.83%) variability. The assay is suitable for erythrocytes, liver, kidney, and bacterial samples, offering advantages over traditional methods by enabling measurements at low hydrogen peroxide concentrations without enzyme inhibition. The method is efficient, accurate, and applicable in clinical and research settings.

Additionally, another novel method was introduced by Hamza and Hadwan (2020). This study introduces a simple and cost-effective spectrophotometric method to measure CAT activity, an essential antioxidant enzyme that breaks down hydrogen peroxide (H₂O₂) into water and oxygen. The assay quantifies unreacted H₂O₂ using a hydroquinone/anilinium sulfate/ammonium molybdate reagent, forming a purple quinone derivative with peak absorbance at 550 nm. The method demonstrated high precision, with within-run and between-run coefficients of variation of 2.6 and 4.7%, respectively, and a strong correlation (*r* = 0.9982) with the peroxovanadate reference method. It was successfully applied to liver and bacterial homogenates, proving to be rapid, accurate, and reproducible. Due to its simplicity and reliability, this assay is well-suited for both research and clinical diagnostics.

The CAT activity assay, as outlined by Engelbrecht et al. (2023), provides a simple yet robust method for quantifying H₂O₂ decomposition through residual titration with potassium permanganate (KMnO₄). This technique measures the absorbance of residual KMnO₄ at 490 nm after CAT-mediated H₂O₂ breakdown, with results expressed as μM H₂O₂ decomposed per minute per milligram of protein. A major advantage of this assay is its reliance on standard laboratory equipment and reagents, making it particularly accessible to researchers in the Global South. Additionally, the 96-well microplate format enhances efficiency by enabling high-throughput processing of multiple samples.

Validation studies have confirmed the assay’s reliability, demonstrating its ability to detect significant changes in CAT activity in cells exposed to agrochemical mixtures. This underscores its utility in toxicological and environmental health research. Notably, CAT activity typically ranges between 5–30 mU/mg protein in cell lysates and tissue homogenates, as reported by Weydert and Cullen (2010). Beyond spectrophotometric methods, complementary techniques such as native activity gels have been used to visualize enzyme overexpression (e.g., achromatic bands in AdCatalase-transduced cells). Furthermore, immunogold electron microscopy (EM) in pancreatic cancer models has localized CAT to peroxisomes, while immunohistochemistry (IHC) revealed its depletion in diseased tissues. However, fibrotic regions may require exclusion due to artifactual staining, highlighting the need for careful interpretation in pathological samples.

Engelbrecht et al. (2023) further adapted this CAT activity assay for cell culture applications, overcoming the limitations of traditional tissue-based spectrophotometric methods. By utilizing a microplate reader, this modified approach simplifies CAT activity measurement while maintaining accuracy and cost-effectiveness. The assay requires minimal reagents and leverages standard cell culture lab equipment, making it ideal for high-throughput screening of environmental chemical mixtures. Similar to the SOD assay, it uses cell supernatants, enabling integrated analysis with other parameters. This streamlined and economical approach enhances antioxidant enzyme studies in mammalian cell lines.

### GPx assay

GPx is a critical antioxidant enzyme that neutralizes harmful hydroperoxides (e.g., H₂O₂) by oxidizing GSH. However, conventional methods for measuring GPx activity, such as NADPH-based and DTNB-based assays, suffer from limitations including low sensitivity, high reagent costs, time-consuming procedures, and susceptibility to interference (Ahmed et al., 2021; Sattar et al., 2024).

To address these challenges, Sattar et al. (2024) developed a modified DTNB-based method that is fast, reliable, and interference-free, eliminating the need for protein precipitation. This method was validated in liver, kidney, erythrocyte, and serum samples, with liver homogenates exhibiting the highest GPx activity. The results showed excellent agreement with the reference method by Rotruck et al. (1973), with a strong correlation (*r* = 0.9991) and high accuracy confirmed by Bland–Altman and Passing-Bablok analyses.

Similarly, Ahmed et al. (2021) introduced an alternative approach using the CUPRAC (cupric reducing antioxidant capacity) assay, which offers several advantages: no protein precipitation requirement (unlike traditional DTNB methods), a short 10-min incubation time, high sensitivity (LOD: 3 U/L, LOQ: 1 U/L), and a broad linear range (2–1000 U/L). Using Box-Behnken Design (BBD), the optimal reaction conditions were determined to be 4 mM GSH, 2 mM peroxide, and 7.5 mM neocuproine. Validation studies demonstrated a near-perfect correlation (*r* = 0.9994) with the standard GPx-DTNB assay, further supported by Bland–Altman and Passing-Bablok analyses.

Beyond these methodological advances, GPx activity has been quantified in various biological contexts. For example, Weydert and Cullen (2010) measured GPx activity (14–30 U/mg protein) in MIA PaCa-2 cells, observing dose-dependent increases upon AdGPx transduction. Native gel electrophoresis revealed GST contamination in bovine GPx controls, while immunofluorescence localized GPx to nuclear and mitochondrial compartments. In tissue samples, GPx activity correlated with immunohistochemical signals, though inflammatory infiltrates required region-specific quantification. Together, these studies highlight how activity assays, electrophoretic techniques, and imaging can be integrated to comprehensively assess GPx regulation across experimental and disease models.

### MDA assays (in-vitro and ex-vivo)

MDA is a reactive carbonyl compound generated as a byproduct of lipid peroxidation; a process triggered by ROS attacking PUFAs such as arachidonic acid. Alongside 4-hydroxy-2-nonenal (HNE) and F2-isoprostanes, MDA is a widely recognized biomarker of oxidative stress, reflecting cellular damage associated with diseases, aging, and lifestyle-related factors. Enzymatic pathways, particularly involving cyclooxygenases (COX) and thromboxane synthase, contribute to MDA formation during eicosanoid metabolism, while non-enzymatic oxidation of PUFAs also generates MDA. Chemically, MDA exists in both free and protein/DNA-adducted forms, and its reactivity due to aldehyde groups renders it both a marker and mediator of oxidative damage. Despite its link to mutagenesis and disease, physiological MDA levels are typically low, though elevated concentrations are observed in conditions such as diabetes, atherosclerosis, and neurodegeneration. Although analytical challenges remain in its accurate quantification, MDA continues to serve as a pivotal biomarker connecting lipid peroxidation to broader pathophysiological mechanisms (Chen & Zhang, 2016; Tsikas, 2017).

Various analytical techniques are employed to measure MDA, leveraging its physicochemical properties such as UV absorbance (245 nm at pH < 3; 267 nm at pH > 7) and reactivity with nucleophilic reagents. Direct spectrophotometric and HPLC–UV methods exist but often lack sensitivity for detecting physiological MDA levels (typically 0–0.5 μM). To overcome this limitation, derivatization strategies targeting MDA’s aldehyde groups or CH-acidic protons are employed. The classic thiobarbituric acid (TBA) assay forms a colored MDA-(TBA)₂ adduct; however, it lacks specificity. More selective approaches include hydrazine-based derivatization using 2,4-dinitrophenylhydrazine (DNPH) for stable hydrazone formation and subsequent HPLC analysis. Advanced methods, such as gas chromatography-mass spectrometry (GC–MS), use pentafluorobenzyl bromide (PFB-Br) to derivatize MDA into (PFB)₂MDA, significantly improving sensitivity and selectivity for biological matrices like plasma and urine (Atiba et al., 2016; Shahidi & Ambigaipalan, 2020; Tsikas, 2017).

Cui et al. (2018) systematically evaluated free (unconjugated) and total (free + conjugated) MDA across various human biospecimens using HPLC-fluorescence analysis. They observed considerable variability in the proportion of free MDA, with the highest levels in exhaled breath condensate (median 48.1%) and the lowest in saliva (3.0%). Strong correlations between free and total MDA in exhaled breath condensate (R^2^ = 0.61) and urine (R^2^ = 0.59) suggest that either form could reliably serve as a biomarker in these matrices. In contrast, weaker correlations in serum (R^2^ = 0.22), nasal fluid (R^2^ = 0.47), and saliva (R^2^ = 0.06) highlight the prevalence of protein-bound MDA in protein-rich specimens. These findings emphasize the importance of selecting appropriate biospecimens and analytical approaches for oxidative stress assessment.

The TBARS assay is a widely used, cost-effective method for evaluating lipid peroxidation by detecting MDA and related byproducts (results usually reported as MDA equivalents). Under acidic conditions (pH 4) and high temperatures (95 °C), MDA reacts with TBA to form a pink-red MDA-(TBA)₂ adduct detectable at 532 nm. However, this method lacks specificity since other carbonyl compounds also react with TBA. HPLC separation of the MDA-(TBA)₂ adduct can improve specificity by reducing interference from non-MDA TBARS. Despite its limitations, the TBARS assay remains a practical screening tool for comparative oxidative stress studies, particularly within the same laboratory environment, provided protocols are standardized (Fauziah et al., 2018; Aguilar Diaz De Leon & Borges, 2020; Senthilkumar, Amaresan, & Sankaranarayanan, 2021).

More refined analytical approaches, such as HPLC, GC–MS, and LC–MS/MS, offer enhanced specificity and sensitivity through derivatization with reagents like DNPH, DAN, or PFB-Br. While TBA derivatization requires harsh, acidic conditions, PFB-Br allows milder, pH-neutral reactions, improving reliability. However, some derivatizing agents (e.g., DAN) may lack specificity, necessitating chromatographic separation. GC–MS/MS analysis of MDA-(PFB)₂ is particularly effective in quantifying both free and protein-adducted MDA in biological samples (Aguilar Diaz De Leon & Borges, 2020).

In biological systems, MDA is predominantly present in protein-bound forms, with free MDA concentrations in healthy human plasma typically below 25 nM. Total MDA (free + bound) can be measured at concentrations of 0.1–3 μM following alkaline hydrolysis to release the bound fraction. However, the nature of the binding-whether covalent or non-covalent—remains incompletely understood. Similar principles apply to HNE analysis, and advanced methods like GC–MS and HPLC enhance the accuracy of quantification (Tsikas et al., 2016).

Animal models such as those using paracetamol or carbon tetrachloride (CCl₄) to induce oxidative stress are often invasive, inconsistent, and more representative of acute liver injury than chronic oxidative stress. These models frequently show elevated MDA and F2-isoprostane levels, but their relevance to human pathophysiology is limited. Morales and Munné-Bosch (2019) proposed a human model involving a single, safe 500 mg dose of paracetamol to assess biomarkers like MDA and nitro-paracetamol. This approach offers greater physiological relevance and reproducibility compared to traditional animal models.

Accurate measurement of MDA is also challenged by pre-analytical factors such as sample handling, storage conditions, and methodological inconsistencies. Hemolysis, the type of anticoagulant used, and prolonged storage can artificially increase MDA levels. Inconsistencies between analytical methods (e.g., TBA assay vs. GC–MS) often result in conflicting outcomes. While elevated MDA levels are associated with various diseases—including diabetes and neurodegeneration-clinical interpretation is complicated by these confounding variables. Thus, standardization of protocols is crucial to reliably correlate MDA levels with oxidative stress (Fauziah et al., 2018).

Beyond its analytical value, the TBARS assay remains a useful method for evaluating the antioxidant activity of compounds within biological systems. Barreira et al. (2013) emphasized the importance of using multiple assays to construct a comprehensive antioxidant profile, considering the multifactorial nature of TAC. Similarly, Rodrigues et al. (2021) highlighted the effectiveness of TBARS and OxHLIA assays in ex vivo models for assessing lipid peroxidation inhibition. The TBARS method also provides insights into protein oxidation via protein carbonyl measurement. Hejazi et al. (2018) demonstrated that H₂O₂-treated tissue showed a 4.9-fold increase in lipid peroxidation and a 4.7-fold increase in protein oxidation, which were significantly attenuated by *Curculigo orchioides* extract. The ethyl acetate (EA) fraction reduced oxidative damage by 2.9-fold, and the aqueous ethyl acetate (AEA) fraction by 3.6-fold, with an inhibition order of L-ascorbate (L-AA) > AEA > EA.

## In vivo antioxidant assays

The in vitro studies offer a cost-effective and controlled platform to explore antioxidant mechanisms using isolated cells or tissues. These models are particularly valuable for initial compound screening due to their simplicity and high-throughput capability. However, they lack the complexity of whole organisms and cannot replicate crucial physiological factors such as metabolism, absorption, or systemic toxicity. As a result, in vitro findings may not reliably predict the biological efficacy or safety of antioxidants in living systems (Warsinah et al., 2020; Kim et al., 2019; Danet, 2021a, b). To address these limitations, in vivo studies are employed to evaluate antioxidant efficacy within the physiological environment of a whole organism. These models account for bioavailability, metabolic transformations, and systemic interactions, providing a more comprehensive understanding of antioxidant activity. In vivo assays assess the real-world effectiveness of antioxidants by measuring oxidative stress biomarkers and related health outcomes across tissues and organs (Sacchet et al., 2015).

**Fig. 2:**
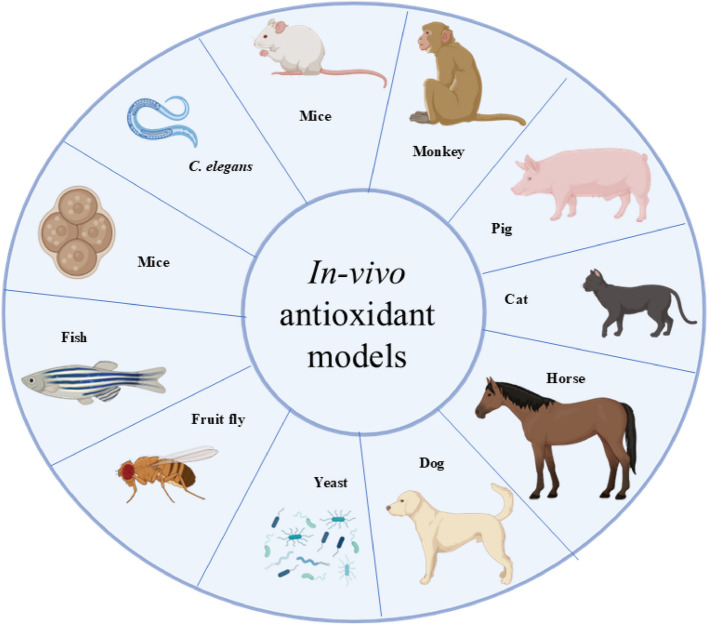
Different in-vivo models used in antioxidant detection

Although in vitro methods like DPPH and ABTS assays are widely used for preliminary screening, their results often diverge from in vivo observations due to differences in compound absorption, distribution, metabolism, and excretion (Warsinah et al., 2020; Kim et al., 2019). The cellular antioxidant activity (CAA) assay has emerged as a useful intermediate approach by incorporating aspects of cellular uptake and bioavailability, thus offering a more physiologically relevant alternative (Loh & Lim, 2018a, b; Slatnar et al., 2012). Additionally, simpler in vivo models such as *Caenorhabditis elegans* provide a practical compromise for early-stage evaluation, thanks to their genetic tractability, short lifecycle, and suitability for high-throughput testing (Torre et al., 2019). Despite the added complexity and ethical considerations associated with animal models, in vivo research is essential for validating antioxidant efficacy and safety prior to human trials. Studies have shown that some compounds exhibit significantly greater antioxidant effects in vivo than suggested by in vitro results, underscoring the importance of biological context (Zahra et al., 2022; Dobrecky et al., 2020). Furthermore, the bioavailability of antioxidant compounds—including their absorption, transport, distribution, and retention—plays a critical role in determining their effectiveness. Animal studies have been instrumental in examining these pharmacokinetic parameters and identifying optimal dosage and delivery mechanisms (Zhang et al., 2017a, b, c). While Fig. 2 illustrates various in vivo models used in antioxidant evaluation, Table 7 summarizes key in vivo antioxidant assays, detailing their mechanisms, experimental models, and measurable parameters. These methodologies provide essential insights into antioxidant interactions within biological systems and are pivotal in advancing fields such as nutrition science, pharmacology, and disease prevention. Moving forward, refining in vivo techniques to enhance reproducibility and reduce ethical concerns will be vital for ensuring reliable translation of research findings into clinical and therapeutic applications (Zahra et al., 2022; Dobrecky et al., 2020).

**Table 7:** In vivo Antioxidant Assays: Mechanisms, Models, and Measured Parameters

Assay Name	Mechanism	Animal Model	Oxidative stress inducer	Measured Antioxidant Parameters	End-product determination	References
Zebrafish antioxidant assay	Real-time observation of antioxidant effects in transparent embryos	Zebrafish (*Danio rerio*	Hydrogen peroxide (H₂O₂)	ROS levels, survival rates	Reduction in ROS levels and improved survival rates	Boix et al., 2020
In vivo SPF testing according to the International Sun Protection Factor Test Method	Measurement of minimum erythemal dose (MED) to determine SPF and UVA-PF	Human subjects with skin phototype I to III	Solar UV simulators (Multiport® 601) to induce erythema	SPF (Sun Protection Factor) and UVA-PF (UVA Protection Factor)	SPF and UVA-PF values, critical wavelength (> 370 nm), and broad-spectrum protection	Peres et al., 2018
C. elegans survival assay	Enhanced stress resistance and antioxidant enzyme activit	*Caenorhabditis elegans*	H₂O₂, methyl viologen (paraquat)	Survival rate, SOD, CAT, GSH-Px, MDA levels	Increased survival time and reduced oxidative damage	Shang et al., 2018; Lang et al., 2024
D-galactose aging model	Reversal of oxidative stress and inflammation	*Kunming mice*	D-galactose	SOD, CAT, GSH-Px, MDA, NO, T-AOC	Increased antioxidant enzyme activity and reduced oxidative stress markers	Zeng et al., 2020a, b
High-fat diet model(dietary antioxidant intervention)	Protection against oxidative injury and lipid peroxidation (In vivo oxidative stress modulation)	*C57BL/6 mice*	High-fat diet (HFD)	GSH-Px activities, SOD/CAT/MDA levels, GSH content	Increased antioxidant enzyme activity and reduced lipid peroxidation	Chen et al., 2018; Tulipani et al., 2011
CCl₄-induced liver damage	Enhancement of antioxidant enzymes and reduction of lipid peroxidation	*Wistar rats*	Carbon tetrachloride (CCl₄)	SOD, CAT, GSH, TBARS	Increased SOD, CAT, and GSH levels; reduced TBARS levels	Sharifi-Rad et al., 2018
ABAP-induced oxidative stress	Modulation of antioxidant enzyme activity and ROS scavenging	*Wistar rats*	2,2′-azobis(2-methylpropionamidine) dihydrochloride (ABAP)	CAT, SOD	Increased CAT and SOD activities	Alencar et al., 2019
Inulin supplementation assay	Modulation of gut microbiota and production of SCFAs	*Laying hens*	None (dietary intervention)	SOD, CAT, GSH-Px, T-AOC, MDA	Increased antioxidant enzyme activity and reduced oxidative stress markers	Shang et al., 2018
RRTP1-1 antioxidant assay	Enhancement of antioxidant enzymes and reduction of oxidative stress markers	*Kunming mice*	D-galactose	CAT, SOD, GSH-Px, TAOC, LPO(Lipid peroxidation), MDA	Increased antioxidant enzyme activity and reduced oxidative stress markers	Chen & Kan, 2018

## Examples of in vivo models and veterinary clinical trials in antioxidant assays

### Fish models

Zebrafish (*Danio rerio*) has emerged as a prominent model organism for in vivo antioxidant studies due to their transparent embryos, rapid development, genetic similarity to humans, and cost-effectiveness. These advantages enable real-time visualization of oxidative stress responses and high-throughput screening of antioxidant compounds, making zebrafish a preferred alternative to traditional mammalian models (Boix et al., 2020; Lin et al., 2023). Moreover, zebrafish share conserved physiological and biochemical pathways with humans, enhancing their relevance for studying human diseases and antioxidant mechanisms. This, combined with their ethical advantages, positions zebrafish as an efficient model for early-stage antioxidant research. For instance, studies using zebrafish embryos have demonstrated that pre-treatment with antioxidant compounds can effectively counteract oxidative damage. In one such study, green tea polyphenols significantly reduced ROS levels and improved survival rates in zebrafish embryos exposed to H₂O₂-induced oxidative stress, underscoring the model's utility in evaluating antioxidant efficacy (Boix et al., 2020).

The study by Rocha-Santos et al. (2018) examined antioxidant responses in the hypoxia-tolerant fish *Piaractus mesopotamicus* (pacu) under oxygen deprivation, comparing GPx and glutathione S-transferase (GST) activities in blood and liver tissues. While GPx activity remained stable in both tissues during hypoxia, GST activity showed substrate-dependent responses: no change with the artificial substrate CDNB but a significant decrease with the physiological lipid peroxidation product 4-HNE, particularly in erythrocytes. Notably, GST activity was threefold higher with 4-HNE than CDNB, identifying 4-HNE as a more sensitive biomarker for hypoxia-induced oxidative stress. The findings demonstrate that blood-based assays can effectively monitor oxidative stress non-lethally, with erythrocyte GST (using 4-HNE) and constitutive GPx activity proving key to maintaining redox balance during oxygen deprivation. This supports the use of minimally invasive blood sampling to assess oxidative stress in ecological and aquaculture studies.

### Caenorhabditis elegans (C. elegans) model

The *C. elegans* is a widely used model in antioxidant and aging research due to its unique biological features and practical advantages. Its short lifespan (approximately 3–4 weeks), small size, transparency, and high reproductive capacity allow for rapid, large-scale experimentation under simple and cost-effective laboratory conditions. These traits make it ideal for high-throughput screening of antioxidant compounds. Importantly, *C. elegans* shares conserved oxidative stress response pathways and aging-related genes with humans, including well-characterized insulin/IGF-1 signaling (IIS) and redox regulatory networks. These similarities enhance its relevance as a translational model for studying human aging and oxidative stress mechanisms (Lin et al., 2023; Zhang et al., 2017a, b, c). The nematode also exhibits age-related degenerative changes analogous to those in mammals, offering insights into the biology of aging, stress resistance, and lifespan extension (Pan & Finkel, 2017; Folch et al., 2018; Baumann et al., 2016).Further supporting its utility, *C. elegans* is highly amenable to genetic manipulation, with a wealth of genetic databases and molecular tools that facilitate mechanistic investigations of natural antioxidants. Researchers can evaluate not only lifespan and stress resistance but also physiological indicators such as protein homeostasis, neuromuscular function, and oxidative stress markers (Nadon, 2006; Mitchell et al., 2025).

Numerous studies have leveraged these advantages to explore antioxidant efficacy. For example, polysaccharide fractions from *Chlorella vulgaris* and *Panax notoginseng* have been shown to significantly extend C. elegans survival under oxidative stress, comparable to the protective effects of vitamin C (Lang et al., 2024; Shang et al., 2018). Specifically, *Chlorella vulgaris* polysaccharides enhanced resistance to H₂O₂-induced oxidative stress, while *Panax notoginseng* polysaccharides boosted the activity of endogenous antioxidant enzymes such as SOD and CAT (Shang et al., 2018). Despite the widespread use of commercial kits to measure antioxidant enzyme activity, earlier studies often lacked C. elegans-specific protocols, potentially affecting result accuracy. To address this, Zhang et al., (2017a, b, c) introduced a refined method for assessing SOD and CAT activity in C. elegans, including optimized sample preparation, protein quantification, and enzyme-specific assays. This standardized approach improves reproducibility and strengthens its application in toxicological and pharmacological evaluations. Overall, *C. elegans* provides a powerful, ethically favorable, and cost-efficient model for antioxidant screening, offering strong parallels to mammalian systems. Its genetic tractability, conserved stress pathways, and capacity for comprehensive phenotypic assessments make it particularly suited for identifying and characterizing natural compounds with potential anti-aging and cytoprotective effects.

### Fruit fly (drosophila melanogaster)

*Drosophila melanogaster* is an emerging model organism for studying food-derived antioxidants due to its conserved oxidative and antioxidative mechanisms shared with mammals, short life cycle, genetic manipulability, and cost-effectiveness compared to rodent models. Key antioxidative mechanisms include the reduction of reactive species, upregulation of endogenous antioxidants, and activation of the Nrf2-ARE pathway. Evaluation methods such as oxidative stress resistance assays and lifespan extension studies are commonly used. As a short-lived invertebrate with well-characterized genetics and neurobiology, *Drosophila* bridges the gap between *C. elegans* and vertebrate models, sharing critical aging-related pathways like TOR (Target of Rapamycin) and JNK (c-Jun N-terminal Kinase) signaling with mammals. This makes it an ideal system for high-throughput screening of dietary antioxidants, lifespan studies, and investigations into age-related degeneration, offering a standardized and efficient approach to bridge in vitro findings with mammalian research (Yi et al., 2021; Lin et al., 2023; Tolwinski, 2017).

*Drosophila melanogaster* has become a valuable model organism in food and nutrition research, with advantages such as a short lifespan (60–80 days), genetic conservation (approximately 60% gene orthologs with mammals), and low-cost maintenance. Its highly conserved metabolic and signaling pathways, along with fewer ethical concerns compared to rodent models, make it ideal for dietary studies. Researchers can utilize either complex solid diets or defined holidic diets to systematically assess nutritional impacts on feed intake, body composition, gut microbiota, aging, and disease-related pathways like inflammation and oxidative stress. Its versatility enables detailed investigation of diet-induced physiological and pathological changes, positioning *Drosophila* as a powerful tool for advancing nutritional science and disease-related research. Future studies should explore its potential in personalized nutrition and mechanistic diet–health interactions (Staats et al., 2018).

A comprehensive guideline established by Wu (2025) presents *Drosophila melanogaster* as a powerful model for studying oxidative stress, offering standardized protocols to assess its multifaceted impacts. The guide details methodologies for evaluating oxidative damage through lifespan analysis (under natural aging or chemical stressors like H₂O₂ and paraquat), ROS quantification (using DCFH-DA and DHE probes), and antioxidant enzyme activities (SOD, CAT, GSH-Px, GST). It further outlines techniques to measure lipid peroxidation (MDA, 4-HNE), protein carbonylation (DNPH method, 3-nitrotyrosine via LC–MS), DNA damage (8-OHdG detection by HPLC–MS/MS), and inflammation markers (RT-qPCR for cytokines). Emphasizing reproducibility, the protocols address sample preparation, reagent formulation, and data analysis, while highlighting *Drosophila*’s advantages—genetic tractability, conserved pathways, and cost-effectiveness. By integrating these approaches, the guideline enables systematic investigation of oxidative stress mechanisms, bridging fundamental research with translational applications in aging and disease.

A study conducted by Zou et al. (2017) demonstrates riboflavin’s anti-aging effects in *Drosophila melanogaster*, showing that lifelong supplementation extends lifespan by 14.1% and enhances reproductive capacity. The research reveals that riboflavin improves oxidative stress resistance, significantly prolonging survival under H₂O₂-induced stress by 20%. Mechanistically, riboflavin boosts endogenous antioxidant defenses, increasing SOD1 and CAT activities while reducing age-related lipofuscin accumulation. These findings suggest riboflavin's potential in slowing aging through antioxidant pathways, highlighting its relevance for human aging research given *Drosophila*’s genetic conservation with mammals and riboflavin’s established safety profile. The study provides compelling evidence for riboflavin’s role in mitigating oxidative damage associated with aging.

Additionally, the protocol by Louka et al. (2025) presents *Drosophila melanogaster* as a model for studying cellular senescence, leveraging its short lifespan and conserved aging pathways with mammals. The method outlines assays to detect senescence markers (e.g., cell cycle arrest, SA-β-gal activity, DNA damage) in fly tissues, while emphasizing biomarker selection and tissue-specific challenges. It addresses the dynamic nature of senescent cells in vivo and aims to improve organism-level detection, bridging gaps between single-cell studies and whole-organism aging research. The approach combines genetic tools with senescence assays, offering a cost-effective platform to explore senescence mechanisms and interventions.

Apart from that, Lopez-Ortiz et al. (2023) have reviewed the potential of *Drosophila* as a translational model for evaluating the impact of antioxidant phytochemicals derived from fruits, vegetables, and spices. These bioactive compounds (e.g., polyphenols, flavonoids, and carotenoids) exhibit health-promoting properties. Due to *Drosophila*’s genetic similarity to humans (approximately 70% gene homology) and its advantages—including low cost, short lifespan, and high reproductive rate—it serves as an ideal model to study the effects of these compounds. The fly’s conserved metabolic pathways enable rapid screening of plant extracts and their active components, providing insights into mechanisms such as antioxidant, anti-aging, and disease-preventive effects. *Drosophila* thus effectively bridges the gap between in vitro studies and mammalian models, offering an efficient platform for evaluating phytochemical benefits on metabolism, longevity, and stress resistance.

### Rodent models

Rodent models, particularly mice and rats, are extensively used in in vivo antioxidant research due to their physiological and metabolic similarity to humans. Rats can be exposed to a wide range of oxidative stress-inducing agents such as ethanol, d-galactose (d-gal), iron, alloxan (ALX), streptozotocin (STZ), carbon tetrachloride (CCl₄), bromobenzene, radiation, noise, methylmercuric chloride (MeHgCl), tert-butyl hydroperoxide (t-BOOH), and hydrogen peroxide (H₂O₂). The antioxidant potential in these models is assessed using numerous biochemical markers, including MDA, CAT, superoxide dismutase (SOD), GSH, GPx, glutathione S-transferase (GST), glutathione reductase (GR), xanthine oxidase (XOD), peroxidase (Px), and others such as alanine aminotransferase (ALT), aspartate aminotransferase (AST), lactate dehydrogenase (LDH), alkaline phosphatase (ALP), γ-glutamyl transpeptidase (γ-GT), ferric reducing antioxidant power (FRAP), protein carbonylation (PC), TAC, ORAC, and 2,2-azino-bis(3-ethylbenzothiazoline-6-sulfonate) (ABTS), among others, providing comprehensive insights into oxidative stress and antioxidant defense mechanisms (Martins et al., 2016). Rodents are also widely employed as aging models due to their physiological similarities with humans; strains such as senescence-accelerated mice (SAM), klotho mutants, and OXYS rats mimic various human aging diseases, making them indispensable in preclinical antioxidant testing (Lin et al., 2023). These models facilitate the evaluation of antioxidant interventions in disease contexts including diabetes, liver injury, and aging. For instance, in d-galactose-induced aging mice, flavonoids like epicatechin, epigallocatechin, and procyanidin B2 reversed oxidative stress by enhancing antioxidant enzymes (SOD, CAT, GSH-Px) and reducing lipid peroxidation (MDA) (Zeng et al., 2020a, b). Similarly, polysaccharides from Fuzhuan brick tea (FBTPS) and *Rosa roxburghii* fruit (RRTP1-1) significantly improved antioxidant status and decreased oxidative markers in models of high-fat diet and d-galactose-induced oxidative stress, respectively (Chen et al., 2018; Chen & Kan, 2018).

Further advancing antioxidant research, Yin et al. (2019) developed a green tea–activated genetic control system for diabetes treatment in mice, utilizing protocatechuic acid (PCA), a green tea metabolite, to regulate therapeutic gene expression precisely, restoring glucose homeostasis in both type 1 and type 2 diabetic models. This system demonstrated remote, diet-triggered therapy and programmable biocomputing capabilities in vivo*.* In a high-fat diet model, supplementation with FBTPS improved antioxidant enzyme activities and reduced MDA levels, indicating enhanced oxidative defense (Chen et al., 2018). The Bribiescas et al. (2025) study explored the relationship between testosterone, oxidative stress (measured via 8-OHdG), and Cu/Zn SOD activity in Shuar males, finding no significant direct correlation but suggesting oxidative stress may not be a major cost of male reproductive effort in this population. Additionally, marine-derived antioxidants such as sulfated polysaccharides from red seaweed (*Gracilaria caudata*) have shown promising in vivo activity by increasing CAT and SOD and reducing oxidative stress markers in rat models (Alencar et al., 2019). Collectively, these findings underscore the versatility of rodent models for assessing a wide spectrum of antioxidant interventions relevant to human health and disease.

### Dogs

Dogs are uniquely valuable models in antioxidant research due to their physiological and genetic similarities to humans, particularly in relation to age-associated oxidative stress and chronic metabolic diseases. Unlike rodents, dogs share comparable lifespans, experience spontaneous diseases such as cancer and neurodegeneration, and develop diet-related conditions including obesity and diabetes, making them highly relevant for studying the long-term effects of antioxidant interventions. Their larger body size allows for repeated sampling (e.g., blood and tissue biopsies) and advanced imaging, which are often not feasible in smaller laboratory animals. Furthermore, dogs exhibit naturally occurring oxidative damage patterns that closely mirror those in humans, providing clinically relevant insights for translational antioxidant therapies. Oxidative stress plays a critical role in the pathophysiology of numerous diseases in both humans and animals. In dogs, erythrocytes are especially vulnerable to oxidative damage, yet the link between oxidative stress and anemia remains underexplored. Milne (2017) found significantly reduced GPx activity in anemic dogs compared to healthy controls, suggesting the involvement of oxidative mechanisms. However, TAC and urinary F2-isoprostane levels showed no significant differences between the groups, indicating a complex interplay of oxidative processes in canine anemia. These findings highlight the need for further investigation into antioxidant-based therapeutic strategies targeting oxidative damage in hematological disorders.

Expanding on this theme, Hagen et al. (2019) conducted a pilot study assessing the effects of a 30-day antioxidant supplementation regimen—including N-acetylcysteine, S-adenosylmethionine, silybin, and vitamin E—in 40 hospitalized dogs with systemic illness. Although plasma vitamin E levels increased significantly (*P* < 0.05), no notable improvements were observed in other oxidative stress markers or clinical outcomes such as survival prediction scores or 30-day survival. This suggests that while antioxidant levels can be modulated biochemically, such combinations may not effectively influence redox balance or disease progression in heterogeneous populations. Future studies should explore targeted antioxidant therapies tailored to specific disease contexts. In the context of canine occupational stress, Sechi et al. (2017) evaluated the impact of antioxidant-enriched diets in therapy dogs participating in animal-assisted interventions (AAI). In a randomized crossover trial involving 11 dogs, those fed the supplemented diet (SD) for 18 weeks showed significant reductions in oxidative stress markers (d-ROMs), triglycerides, and creatinine (*P* < 0.05). In contrast, dogs on the control diet (CD) exhibited increased amylase levels (*P* < 0.01). After crossover, the CD group showed reductions in amylase, while the SD group experienced improved glutamate pyruvate transaminase (GPT) levels (*P* < 0.05). These results support the use of antioxidant-enriched diets to alleviate exercise-induced oxidative stress and enhance metabolic health in working dogs.

The protective role of dietary antioxidants was further evidenced by Campigotto et al. (2020), who investigated the incorporation of curcumin into dog food. After six months of storage, the curcumin-enriched diet demonstrated reduced protein and lipid peroxidation, increased TAC, and preserved nutritional quality. A 42-day feeding trial with Beagle dogs revealed that curcumin supplementation increased red blood cell and neutrophil counts, reduced lymphocyte levels, and enhanced antioxidant enzyme activity. Additionally, it lowered oxidative stress and improved metabolic parameters including serum glucose, urea, triglycerides, and cholesterol—without inducing weight gain. These findings underscore curcumin’s potential as a functional dietary additive with anti-inflammatory and antioxidant benefits. Similarly, Sechi et al. (2015) investigated the effects of antioxidant-rich dietary supplements on oxidative stress and brain-derived neurotrophic factor (BDNF) in aging dogs. In a six-month study involving 36 dogs assigned to four diet groups—including those enriched with *Grifola frondosa*, *Curcuma longa*, and omega-3/6 fatty acids—significant reductions in reactive oxygen metabolites (dROMs) and increases in BDNF were observed. These results suggest that such supplements may help mitigate oxidative stress and support cognitive health in aging dogs, offering a promising non-pharmacological strategy for managing neurodegeneration.

To standardize oxidative stress assessment in dogs, Rubio et al., (2016a, b) reviewed spectrophotometric assays commonly used to measure TAC in serum, including TEAC, ferric reducing ability of plasma (FRAP), and cupric reducing antioxidant capacity (CUPRAC). These methods differ in their mechanisms—radical scavenging (TEAC), ferric ion reduction (FRAP), and cupric ion reduction (CUPRAC)—and, while practical and cost-effective, are limited in scope. The authors emphasize the need for combining multiple assays with specific antioxidant measurements to achieve a comprehensive understanding of oxidative status in both clinical and research settings. Recent findings by Jewell et al. (2024) demonstrate the beneficial impact of supplementing canine diets with antioxidants such as vitamin E, vitamin C, and β-carotene. Increasing dietary vitamin E up to 1,500 IU/kg led to a dose-dependent elevation in serum vitamin E, with optimal response at 1,000 IU/kg. Supplementation significantly decreased DNA damage, as evidenced by the Comet assay and reduced levels of 8-hydroxy-2′-deoxyguanosine, a marker of oxidative DNA damage. These results affirm the protective potential of antioxidant intake in reducing free radical-induced cellular damage and enhancing immune health in dogs.

Dietary fatty acid supplementation also interacts with redox balance. Risso et al. (2021) studied the impact of fish oil (FO), alone and in combination with vitamin E (VE), on antioxidant activity in canine seminal plasma. In a 3 × 3 Latin square design involving six dogs, FO alone significantly decreased TAC (as measured by ABTS and FRAP assays) and increased oxidative stress (T-SH levels). However, FO + VE co-supplementation preserved antioxidant levels and reduced oxidative stress, emphasizing the importance of combining antioxidants with high-PUFA diets to avoid redox imbalance. FO also reduced serum triglycerides, though no other hematological changes were noted.

Antioxidants also play a vital role in improving outcomes in canine assisted reproductive technologies (ARTs), which face species-specific challenges such as prolonged estrous cycles and limited oocyte competence. Ciani et al. (2021) reported that cryopreservation leads to oxidative sperm DNA damage, but antioxidants such as metformin, curcumin, and myoinositol improve post-thaw motility and membrane integrity. Moreover, vitrification protocols with trehalose and soy lecithin support sperm viability, while enzymatic antioxidants (SOD, CAT, GPx) in semen extenders help maintain quality during refrigeration. Though advancements have been made, the application of antioxidants in canine ARTs remains an evolving area of research.

Lastly, Wang et al., (2016a, b) examined the effects of a dietary antioxidant blend—including lutein, zeaxanthin, β-carotene, astaxanthin, and vitamins C and E—on retinal function and refractive development in healthy adult Beagles. Over six months, electroretinography (ERG) revealed significant improvements in both scotopic and photopic retinal responses (*P* < 0.05), while refractive error progression was reduced compared to controls (*P* < 0.05). These results are consistent with human clinical data and suggest that antioxidant supplementation may help preserve visual function and delay age-related ocular changes in dogs.

### Cats

Cats offer unique advantages in antioxidant research due to their distinct physiology and metabolic traits, particularly their status as obligate carnivores with unique taurine metabolism and susceptibility to oxidative stress-related disorders. Unlike traditional rodent models, cats naturally develop conditions such as diabetes mellitus, hepatic lipidosis, chronic kidney disease, and cognitive dysfunction—diseases with strong oxidative stress components that also occur in humans. These similarities make cats valuable translational models for investigating antioxidant therapies. Moreover, their relatively long lifespan allows for extended studies on chronic oxidative damage and the long-term impact of antioxidant interventions.

Nutritional supplementation with antioxidants has shown promising effects in feline models. Jewell et al. (2024) demonstrated that diets enriched with vitamin E, vitamin C, and β-carotene improved serum vitamin E levels and cellular protection in cats. While DNA integrity, assessed by the Comet assay, improved with higher antioxidant intake, no significant reduction in 8-hydroxy-2′-deoxyguanosine (8-OHdG) levels was observed, in contrast to results seen in dogs. However, total antioxidant power (TAP) increased linearly with vitamin E supplementation, indicating improved systemic antioxidant capacity. These findings highlight species-specific metabolic responses and emphasize the need for further research to establish optimal antioxidant dosages tailored to feline physiology.

Beyond nutrition, antioxidants play a critical role in assisted reproductive technologies (ARTs) such as in vitro fertilization and sperm cryopreservation, which are particularly relevant to the conservation of endangered feline species. Ciani et al. (2021) reported that ROS negatively affect gamete quality during in vitro procedures. Antioxidants like SOD, CAT, and GSH have been shown to enhance oocyte maturation and embryo development. For example, supplementing ovary transport media with SOD reduces apoptosis in cumulus-oocyte complexes, while cysteine and vitamin E improve post-thaw sperm motility and DNA integrity. Additionally, resveratrol enhances embryo development after ovarian tissue storage, reinforcing the therapeutic value of antioxidants in improving ART outcomes.

Oxidative stress is also implicated in feline cardiovascular disease. Michałek et al. (2020) investigated oxidative biomarkers in cats diagnosed with hypertrophic cardiomyopathy (HCM), comparing symptomatic (*n* = 8), asymptomatic (SUB-HCM, *n* = 11), and healthy control cats (*n* = 11). Both HCM groups showed significantly reduced SOD activity, while CAT activity was selectively diminished in asymptomatic cats. No significant changes were detected in GPx, TAC, or lipid peroxidation markers. These findings suggest compromised antioxidant defenses, especially SOD depletion across disease stages, and early CAT reduction, which may contribute to the progression of cardiomyopathy.

Inflammatory conditions in cats also reflect the burden of oxidative stress. Rudenko et al. (2021) assessed oxidative stress markers in cats with aseptic inflammation (e.g., post-ovariohysterectomy) and purulent-inflammatory diseases such as wounds, abscesses, and sepsis. Mild oxidative stress was observed in aseptic cases, characterized by modest increases in diene conjugates (1.4-fold) and SOD activity (1.38-fold). In contrast, sepsis triggered severe oxidative damage, with substantial elevations in diene conjugates (4.4-fold), MDA(8.4-fold), and medium-weight molecules (8.8-fold), along with marked depletion of SOD and CAT. Interestingly, total antioxidant activity (AOA) rose in localized purulent infections but significantly declined in septic cats, indicating a systemic oxidative imbalance and highlighting the potential benefit of antioxidant-based therapies in managing feline inflammatory conditions.

Cognitive health in aging cats is similarly impacted by oxidative stress. Castillo and Hernández (2015) linked feline Cognitive Dysfunction Syndrome (CDS) to neurodegenerative changes exacerbated by ROS, including beta-amyloid deposition and neuronal loss. Aging cats exhibit diminished antioxidant enzyme levels, particularly SOD and CAT, along with elevated lipid peroxidation, all of which contribute to cognitive decline. Nutritional interventions including vitamin E, S-adenosylmethionine, omega-3 fatty acids, and mitochondrial cofactors like L-carnitine have been proposed to reduce oxidative damage and support cognitive function, offering a dietary strategy for managing age-related neurodegeneration in cats.

The connection between oxidative stress and infectious disease is further illustrated by feline coronavirus (FCoV) infections. Kayar et al. (2015) reported significantly elevated total oxidant capacity (TOC), nitric oxide (NO), and MDA levels in FCoV-seropositive cats, along with a marked reduction in TAC. These results indicate a substantial oxidative burden during FCoV infection and suggest a potential role for antioxidant supplementation in reducing viral pathogenicity and supporting immune function.

Natural antioxidants, such as bixin—a carotenoid derived from *Bixa orellana*—have also been studied in cats. Park et al., (2016a, b) found that bixin is rapidly absorbed into feline plasma and accumulates in leukocyte mitochondria. Supplementation enhanced both humoral and cell-mediated immunity, increasing plasma IgG levels and lymphoproliferative responses. It also significantly reduced oxidative DNA damage, as indicated by lower plasma 8-OHdG levels. However, higher doses (10 mg/day) negatively impacted vaccine-induced immune responses, pointing to the importance of careful dose optimization in antioxidant supplementation. In therapeutic contexts, antioxidant supplementation has shown benefits in endocrine disorders such as hyperthyroidism. Candellone et al. (2020) conducted a blinded randomized controlled trial in hyperthyroid cats undergoing methimazole (MMI) treatment. Cats receiving a combination of antioxidants—including curcumin, quercetin, resveratrol, and vitamin E—showed significantly reduced oxidative stress biomarkers (dROMs and oxidative stress index) and higher antioxidant capacity as measured by the OXY-adsorbent test. Moreover, the incidence of MMI-related side effects was notably lower in the antioxidant group (12.5%) compared to the placebo group (35.7%), suggesting that antioxidant co-supplementation may improve therapeutic efficacy and reduce drug-related adverse effects in feline hyperthyroidism.

### Horses

Horses serve as valuable models in antioxidant research due to their exceptional athletic physiology, high metabolic rate, and susceptibility to exercise-induced oxidative stress—conditions that closely parallel those seen in human athletes. Their large body size allows for longitudinal sampling (e.g., blood, muscle biopsies) and the use of advanced imaging technologies, which are limited in small animal models. Horses also develop naturally occurring oxidative stress-related disorders, such as equine metabolic syndrome and recurrent exertional rhabdomyolysis, which provide translational insights into antioxidant therapies for muscle and metabolic diseases. Furthermore, age-related conditions like equine Cushing’s disease exhibit oxidative damage patterns similar to those found in humans, making horses ideal for studying long-term antioxidant interventions. Ceja-Garcia et al. (2022) demonstrated the efficacy of dietary antioxidants—vitamins E, C, and β-carotene—in mitigating oxidative stress in horses triggered by exercise, transportation, and dietary shifts. These antioxidants act synergistically: vitamin E stabilizes cell membranes, vitamin C regenerates vitamin E and scavenges free radicals, and β-carotene supports immune function. This supplementation is particularly beneficial for performance horses, breeding mares, and stallions, enhancing their health and resilience. Additionally, Bahena Culhuac et al. (2023) highlighted the importance of selenium, a trace element essential for GPx activity, in maintaining redox balance, especially during stress, reinforcing the necessity of micronutrient inclusion in equine dietary management.

Beyond conventional vitamins and minerals, phytogenic feed additives are gaining attention for their antioxidant potential. Elghandour et al. (2018) examined plant-derived bioactives such as ginger, garlic, ginseng, and flaxseed, noting their antioxidative, anti-inflammatory, and immunomodulatory effects in equine nutrition. These compounds show promise for managing oxidative stress-related conditions including gastric ulcers and skin disorders. However, the authors cautioned that efficacy depends on multiple factors including dosage, duration, and individual variability, and emphasized the need for standardized studies to assess safety and optimal administration. Supporting this, Melo et al. (2016) explored the combined use of polyunsaturated oils and vitamin E in horses under different training regimens. Supplemented animals exhibited enhanced antioxidant responses—including increased activity of SOD, GPx, and higher uric acid levels—without adverse changes in hematological parameters. These findings suggest that such dietary strategies can improve oxidative defense mechanisms under both maintenance and physical exertion conditions.

Environmental and management factors also significantly influence oxidative status in horses. Bażanów et al. (2020) compared oxidative markers between Hucul horses raised in semi-natural environments and commercially managed Arabian horses. Hucul horses displayed higher SOD activity and lower levels of oxidative stress markers such as lipofuscin, MDA, and total oxidant status. Minimal gender differences were observed, although Hucul mares exhibited slightly higher antioxidant enzyme activity. These results suggest that natural husbandry conditions may bolster innate antioxidant defenses, while intensive management practices could elevate oxidative stress. In the context of equine assisted reproductive technologies (ARTs), antioxidants also play a vital role in preserving sperm quality during cooling and cryopreservation. Ciani et al. (2021) reported that nitric oxide enhances sperm motility post-thaw, while dietary supplements like *Lepidium meyenii* (Maca) improve sperm viability. Coenzyme Q10 and α-tocopherol were found to reduce lipid peroxidation and support motility in cooled semen. Seasonal variation also influenced outcomes, with improved cryopreservation results observed in spring and autumn. Although some additives like caseinate and lactoferrin demonstrated limited effectiveness, these findings underscore the importance of continued research into novel antioxidants to optimize fertility outcomes in equine ARTs.

### Pigs

Pigs (*Sus scrofa*) are highly valuable in antioxidant research due to their close physiological and metabolic similarities to humans. Unlike rodents, pigs naturally develop obesity, diabetes, and cardiovascular diseases, exhibiting oxidative stress patterns that mirror human conditions. Their human-like skin, omnivorous diet, and cardiovascular system make them especially useful for studying dietary antioxidants and UV-induced oxidative damage. Additionally, their large size allows for repeated clinical sampling and advanced imaging, bridging the gap between in vitro assays and human clinical trials. In swine production, oxidative stress arises from stressors such as weaning, mycotoxin exposure, environmental fluctuations, and intensive management systems. Hao et al. (2021) emphasized the detrimental effects of redox imbalance on pig health and productivity, identifying regulatory pathways such as Keap1/Nrf2, MAPK, and AMPK as central to oxidative control. Nutritional interventions using functional amino acids (e.g., cysteine, arginine), vitamins (E, A, C), trace minerals (zinc, selenium), and natural antioxidants (curcumin, resveratrol) play crucial roles in reinforcing endogenous antioxidant defenses. This was further supported by Vergauwen et al. (2016), who demonstrated the protective effects of rosmarinic acid, quercetin, gallic acid, and selenium-methionine on porcine intestinal epithelial cells under H₂O₂-induced stress. These compounds improved cell viability, decreased intracellular ROS, and maintained epithelial barrier integrity, highlighting their potential as gut-protective dietary supplements.

Phytochemicals also offer promise in enhancing antioxidant capacity in pigs. Selby-Pham et al. (2017) showed that red cabbage and grape skin extracts enhanced both enzymatic (GPx) and non-enzymatic (TEAC) antioxidant activities, while also inducing mild ROS formation in vitro—suggesting a hormetic, adaptive cellular response. Similarly, Lebret and Čandek-Potokar, (2022) and Moreno et al. (2020) reported that natural foraging conditions and dietary supplements like vitamin E and polyunsaturated oils can enhance antioxidant defenses and meat quality. However, Silva-Guillen et al. (2020) found that feeding peroxidized soybean oil to newly weaned pigs impaired growth and antioxidant status, with only partial recovery upon vitamin E or polyphenol supplementation—underscoring the importance of proactive dietary management. A meta-analysis by Semenova et al. (2020) supported the use of adaptogenic antioxidants like organic selenium and flavonoids (e.g., quercetin, dihydroquercetin) to mitigate stress-induced myopathies and improve oxidative stability in meat, though optimal dosing strategies remain under investigation. Orengo et al. (2021) demonstrated that antioxidant blends (AOX1, AOX2) can effectively compensate for low vitamin E levels without compromising growth performance. Moreover, Yun et al. (2016) showed that wild ginseng root extract restored antioxidant markers and male reproductive function in vitamin C-deficient guinea pigs, suggesting a broader application of plant-based antioxidants in reproductive health. Finally, Ortega and Szabó (2021) highlighted how heat stress disrupts gut barrier integrity through oxidative and inflammatory pathways. Supplementation with antioxidants—including vitamins A, C, D, and E, selenium, and zinc—restored tight junction proteins and intestinal morphology, although crypt recovery remained incomplete. Collectively, these studies emphasize the multifaceted role of antioxidants in supporting pig health, performance, and translational biomedical research.

### Monkeys

Monkeys (e.g., macaques, marmosets) are unparalleled in antioxidant research due to their genetic, physiological, and metabolic proximity to humans, offering superior translational relevance compared to rodents or pigs. Their complex nervous, cardiovascular, and immune systems allow for the study of age-related oxidative damage (e.g., neurodegeneration, atherosclerosis) with human-like progression. Unlike smaller animal models, monkeys spontaneously develop chronic diseases such as diabetes and Parkinson’s disease, which are strongly linked to oxidative stress, enabling direct testing of antioxidant therapies. Their longer lifespan also permits long-term intervention studies, and their shared drug metabolism improves the predictability of clinical outcomes. For example, Carvalho-Queiroz et al. (2015) investigated the potential of antioxidant enzymes—Cu–Zn SOD and GPX—as vaccine candidates against *Schistosoma mansoni* in olive baboons. Immunization was well-tolerated and significantly reduced egg loads and clinical symptoms despite not meeting WHO benchmarks for worm reduction, suggesting that antioxidant-based vaccines can reduce disease pathology and transmission by modulating immune responses. In a Parkinson’s disease (PD) model, Li et al., (2017a, b) observed progressive declines in serum antioxidant enzyme activities (SOD, GPX, GST) and increases in oxidative damage markers (e.g., MDA and GSH), accompanied by compensatory gene upregulation and dopaminergic neuronal loss. Follow-up therapeutic interventions in this model showed that levodopa and clioquinol not only improved motor symptoms but also restored antioxidant enzyme activities and reduced lipid peroxidation, suggesting that their clinical benefit may partially derive from redox modulation. Aydemir and Ulusu (2022) extended antioxidant considerations to viral infections, proposing antioxidants such as N-acetyl-L-cysteine, resveratrol, and terameprocol as potential therapeutic agents in monkeypox virus (MPXV) infection, which is known to disrupt host redox balance. In ocular research, Siegfried et al. (2017) showed that intraocular antioxidant capacity decreases following surgical interventions like vitrectomy and cataract removal in both humans and rhesus monkeys, correlating with elevated oxidative DNA damage (8-OHdG); notably, African Americans had higher 8-OHdG levels, potentially contributing to increased glaucoma risk. On a mechanistic level, Franco et al. (2019) reviewed how phytochemicals exert indirect antioxidant effects primarily by modulating signaling pathways and inducing endogenous defenses (e.g., vitagenes, detoxification enzymes), while also cautioning about pro-oxidant risks and the need for dosage optimization. In a translational application, Yin et al. (2019) developed a phenylacetic acid (PCA)-responsive gene circuit system for diabetes treatment in cynomolgus monkeys. Engineered, microencapsulated cells restored insulin secretion and glucose tolerance in diabetic monkeys, with second-generation systems enabling oral PCA-triggered GLP-1 regulation using dietary green tea as a PCA source—demonstrating a safe and non-invasive redox-sensitive therapeutic approach. Collectively, these studies—ranging from parasitic and viral infections to neurodegeneration, ocular disorders, and metabolic diseases—underscore the pivotal role of oxidative stress in disease pathophysiology and highlight the potential of antioxidant-based strategies, both direct and adjunctive. The use of non-human primate models provides unmatched translational fidelity, paving the way for clinical applications in human antioxidant therapy and precision medicine.

### Gut microbiota strains

Gut microbiota-based models offer distinct advantages in antioxidant research due to their unique metabolic interactions and ability to produce bioactive metabolites such as short-chain fatty acids (SCFAs) and polyphenol-derived compounds, which modulate oxidative stress pathways in ways that differ from traditional in vivo models relying on direct antioxidant administration. These effects are often strain-specific and influence key host signaling cascades such as Nrf2 and NF-κB, distinguishing microbiota-mediated responses from those elicited by synthetic antioxidants. Furthermore, microbiota-focused studies typically require germ-free or gnotobiotic animals to isolate microbial contributions, unlike conventional in vivo models that employ chemical or diet-induced oxidative stress. This specificity reveals the dual role of gut microbiota in both exacerbating and alleviating oxidative damage, paving the way for targeted, probiotic-based interventions. For example, Noureen et al. (2018) assessed the antioxidant properties of four *Lactobacillus brevis* strains of equine origin and found that *L. brevis* MG882402 exhibited the highest radical scavenging and lipid peroxidation inhibitory activity in both intact cells and cell-free supernatants, outperforming established commercial probiotics such as *L. acidophilus* ATCC 4356, *Bifidobacterium longum* BB536, and *L. rhamnosus* GG ATCC 53103. These results suggest strong potential for developing high-performing probiotic strains for both veterinary and human applications. In addition to probiotic effects, prebiotics like inulin have been shown to enhance antioxidant defenses by modulating microbial fermentation and SCFA production. In laying hens, inulin supplementation led to increased activity of antioxidant enzymes (SOD, CAT, GSH-Px) and reduced serum MDA, likely via SCFA-mediated pathways (Shang et al., 2018). Mechanistically, antioxidant effects may result from direct upregulation of endogenous defense enzymes (Li et al., 2015; Yu et al., 2016), or indirect modulation through gut microbiota shaping, particularly by polysaccharides and polyphenols (Shang et al., 2018). However, it is important to note that in vitro antioxidant capacity often fails to predict in vivo efficacy due to extensive metabolic transformation and host-specific interactions (Veskoukis et al., 2012; Kasote et al., 2015), emphasizing the necessity of integrated experimental models to fully understand microbiota-mediated antioxidant responses.

### Yeast (saccharomyces cerevisiae)

As a simple single-cell model, yeast is widely used in aging research due to its short lifespan and well-conserved metabolic pathways, making it particularly useful for studying oxidative stress, mitochondrial dysfunction, and genomic instability. Its cost-effectiveness also enables high-throughput screening of anti-aging compounds (Lin et al., 2023). Building on this advantage, Gosselin-Monplaisir et al. (2023) developed the Anti Oxidant Power in Yeast (AOPY) assay, a high-throughput method using *Saccharomyces cerevisiae* to quantify intracellular antioxidant activity. By optimizing parameters such as cell density (OD600 = 0.6), Thiazole Orange (TO) concentration (64 µM), and exponential-phase cultures, the assay effectively measured ROS scavenging by exogenous antioxidants (e.g., resveratrol, quercetin) and endogenous carotenoids (e.g., β-carotene) produced via synthetic pathways. Notably, the AOPY assay demonstrated a strong correlation between intracellular β-carotene levels and reduced oxidative stress, offering a rapid, in vivo alternative to traditional extraction-based methods. This versatile tool bridges synthetic biology and industrial applications, enabling real-time monitoring of antioxidant production in microbial cell factories (Gosselin-Monplaisir et al., 2023). Further extending these findings, Assalve et al. (2024) investigated the in vivo antioxidant effects of common dietary flavonoids—quercetin, apigenin, luteolin, naringenin, and genistein—using *S. cerevisiae*. Their study revealed that these flavonoids were non-toxic and significantly enhanced yeast cell viability under oxidative stress induced by H₂O₂, reducing intracellular ROS levels, decreasing protein carbonylation, and modulating antioxidant defenses. These results highlight their potential as dietary supplements or therapeutic agents for oxidative stress-related disorders. Similarly, Zahoor et al. (2022) explored the antioxidant and anti-aging potential of flavonoids extracted from medicinal plants, including quercetin analogs like morin, kaempferol, aromadendrin, and steppogenin. Their work demonstrated that these compounds protected yeast cells against oxidative stress induced by acetic acid, reducing ROS and extending chronological lifespan by 15–25%. Gene expression analysis further revealed that morin counteracted acetic acid-induced upregulation of *TOR1* and *MSN2/MSN4*, suggesting a modulatory role in stress response. Collectively, these studies underscore yeast’s utility as an efficient screening tool for identifying anti-aging compounds from natural sources while providing insights into conserved aging mechanisms and potential applications in functional foods, cosmetics, or therapeutics.

## Human clinical studies in assessing antioxidant activities

Human clinical studies are crucial for assessing antioxidant activities because they provide direct evidence of efficacy, safety, and physiological relevance in humans. While preclinical studies (in vitro or animal models) can suggest potential benefits, they cannot fully predict how antioxidants will behave in the human body. Clinical trials determine whether these compounds are effectively absorbed, metabolized, and utilized to combat oxidative stress in real-world conditions. Additionally, they establish safe dosage ranges, identify potential side effects, and validate whether antioxidant supplementation actually translates into measurable health benefits, such as reduced disease risk or improved biomarkers of oxidative damage. Without human trials, antioxidant claims remain speculative, lacking the scientific rigor required for medical and nutritional applications.

Oxidative stress biomarkers play a crucial role in clinical trials by evaluating disease progression, assessing therapeutic interventions, and monitoring redox imbalances associated with aging and chronic conditions (Marrocco et al., 2017). However, challenges persist in standardizing measurements of ROS, oxidative damage markers, enzymatic antioxidants, and TAC. To address these limitations, researchers employ integrated approaches such as composite indices (e.g., OXY-SCORE, oxidative-INDEX), which combine multiple biomarkers to provide a more comprehensive assessment of oxidative status (Marrocco et al., 2017). These tools have proven valuable in cardiovascular research, oncology, and nutrition studies, where they help correlate antioxidant interventions with improved health outcomes. Clinically useful biomarkers must demonstrate diagnostic relevance, stability in accessible biospecimens, and cost-effectiveness. Future directions include developing disease-specific biomarker panels, dynamic monitoring systems, and integration with advanced omics technologies to enhance precision medicine applications in oxidative stress management (Jeszka-Skowron et al., 2021b).

In cancer research, oxidative stress biomarkers have been extensively studied due to their role in tumorigenesis and response to therapy. Katerji et al. (2019) reviewed the impact of ROS in cancer progression, highlighting DNA damage (e.g., 8-hydroxy-2′-deoxyguanosine, 8-OHdG), lipid peroxidation (e.g., malondialdehyde, MDA), and protein oxidation (e.g., protein carbonyls) as key indicators. Clinical trials have demonstrated elevated oxidative stress and compromised antioxidant defenses (e.g., reduced SOD, CAT, and GSH) in various cancers, including breast, colorectal, and liver cancer. However, challenges such as biomarker variability and lack of standardized protocols limit their clinical translation. Future research should focus on targeted antioxidant therapies and improved validation methods for cancer diagnosis and treatment monitoring (Katerji et al., 2019).

A clinical trial by Zedan et al. (2015) investigated oxidative stress in vitiligo by measuring serum GPx activity. The results showed significantly lower GPx levels in vitiligo patients compared to healthy controls, indicating impaired antioxidant defense. Moreover, GPx activity varied with skin type, being lowest in individuals with lighter skin (type III). Although trends related to disease type and duration were observed, they did not reach statistical significance. These findings support the role of oxidative stress in vitiligo pathogenesis and highlight the potential of antioxidant-based therapies.

In a related context, a pilot study by Alzoghaibi and BaHammam (2005) examined oxidative stress and inflammation in patients with severe obstructive sleep apnea (OSA). The study measured lipid peroxidation (TBARS), SOD activity, and inflammatory markers (IL-8, GCP-2). While no significant differences were found in SOD activity (0.29 vs. 0.31 U/mL) or lipid peroxidation between OSA patients and healthy controls, suggesting a lack of major oxidative stress imbalance, levels of IL-8 and GCP-2 were significantly elevated in OSA patients (198.8 vs. 180.83 pg/mL and 383.34 vs. 218 pg/mL, respectively). These results indicate that although oxidative stress markers remained stable, OSA is associated with systemic inflammation, which may contribute to its cardiovascular comorbidities.

Recent clinical trials have investigated the antioxidant potential of bioactive compounds such as curcumin. A meta-analysis of randomized controlled trials (*n* = 308) found that curcumin supplementation (mean 645 mg/day for 67 days) significantly increased TAC (*p* = 0.045) and reduced MDA levels (*p* = 0.086), particularly in high oxidative stress populations like obese individuals and β-thalassemia patients (Jakubczyk et al., 2020). These studies employed rigorous methodologies, measuring key biomarkers (TAC, MDA, CAT, GPx) to assess therapeutic efficacy. Disease-specific benefits were observed, including cardiovascular protection (reduced MDA post-CABG, *p* < 0.001) and improved gastric mucosa in *H. pylori* infections (*p* < 0.05) when combined with conventional therapy. However, limitations such as variable bioavailability (e.g., enhanced effects with piperine co-administration) and differential responses in diabetic populations underscore the need for personalized intervention strategies (Jakubczyk et al., 2020).

A clinical experiment conducted by Altuhafi et al. (2021) investigated the role of oxidative stress in diabetic nephropathy by analyzing the activity of selenium-dependent GPx and other oxidant/antioxidant markers in diabetic patients. The case–control study included 180 participants: 60 healthy controls (G1), 60 type 2 diabetic (T2D) patients without nephropathy (G2), and 60 T2D patients with nephropathy (G3). Blood samples were analyzed for GPx activity (total, Se-dependent, and non-Se-dependent), total oxidant status (TOS), lipid peroxidation (MDA), TAC, and CAT activity. The results showed significantly reduced GPx activity in the diabetic groups (G2 and G3) compared to the controls, with the lowest levels observed in nephropathy patients (*p* < 0.05). Oxidative stress markers (TOS and MDA) were elevated, while antioxidant defenses (TAC and CAT) were diminished in diabetic patients, particularly in G3. The decline in Se-dependent GPx activity correlated with disease progression, suggesting impaired detoxification of ROS in diabetic nephropathy. The study concludes that GPx deficiency exacerbates oxidative stress in diabetes, contributing to renal damage. The findings highlight the importance of antioxidant mechanisms in mitigating diabetic complications and suggest potential therapeutic targets, such as selenium supplementation, to enhance GPx activity and reduce oxidative injury in diabetic nephropathy.

Astaxanthin, a potent carotenoid, has also been studied in clinical trials for its superior antioxidant capacity compared to vitamins like α-tocopherol. Human trials demonstrate its neuroprotective effects, improving cognitive function in age-related decline and reducing oxidative stress in neurodegenerative diseases (Donoso et al., 2021). Additionally, astaxanthin exhibits cardioprotective benefits by lowering LDL oxidation and inflammatory markers, with optimal dosing ranging from 4–12 mg/day. Its unique ability to protect both cell membranes and intracellular components makes it a promising therapeutic agent for oxidative stress-related disorders (Donoso et al., 2021).

Dietary TAC has been explored as a tool to assess the cumulative antioxidant capacity of food intake. A cross-sectional study (*n* = 266) found that higher dietary TAC was inversely associated with glycemia, lipid biomarkers (total cholesterol:HDL-c ratio, triglycerides, oxidized-LDL), and central adiposity (Hermsdorff et al., 2011). Plasma TAC also negatively correlated with ox-LDL (*r* = −0.20, *p* = 0.003), suggesting a protective role against early atherogenesis. These findings highlight the importance of antioxidant-rich diets in metabolic health (Hermsdorff et al., 2011).

Despite promising findings, challenges remain in clinical antioxidant research, including biomarker variability, inconsistent dosing, and methodological limitations. Future trials should focus on standardized protocols, larger sample sizes, and integration with multi-omics approaches to enhance precision in antioxidant therapy (Jeszka-Skowron et al., 2021a). By refining these methodologies, clinical trials can better validate antioxidant interventions and advance personalized treatment strategies for oxidative stress-related diseases.

Polidori et al. (2004) investigated oxidative stress and antioxidant status in congestive heart failure (CHF) patients, comparing 30 CHF patients (NYHA class II-III) with 30 matched controls. The researchers measured plasma levels of the lipid peroxidation marker 8, 12-isoprostane F2α-VI, antioxidants (vitamins A, C, E, carotenoids, uric acid), and antioxidant enzyme activities (SOD and GPx). Results revealed significantly higher isoprostane levels (*p* < 0.0001) and lower antioxidant levels in CHF patients, with oxidative stress markers correlating strongly with disease severity. For instance, class III patients exhibited 40% higher isoprostane levels than class II patients (*p* = 0.0012). Inverse correlations between ejection fraction and oxidative stress markers, alongside positive correlations with antioxidant levels, underscored the role of oxidative damage in CHF progression, suggesting potential benefits from antioxidant interventions.

Similarly, oxidative stress has been implicated in neurodegenerative and pregnancy-related disorders. Kamelia et al. (2020) evaluated the antioxidant effects of banana (*Musa paradisiaca* Linn.) ethanol extract (BE) on F2-isoprostane levels in a Wistar rat model of Alzheimer’s disease (AD). After inducing AD with Aβ1-42, rats treated with BE (250–1000 mg/kg BW) showed significant reductions in F2-isoprostanes (*p* < 0.05), with the highest dose yielding the most pronounced effect. In contrast, untreated AD rats exhibited escalating oxidative stress. These findings highlight BE’s potential to counteract Aβ-induced oxidative damage, likely due to its flavonoid and polyphenol content.

Further emphasizing the broad relevance of oxidative stress, Brien et al. (2017) explored its role in preeclampsia (PE) by analyzing placental F2-isoprostanes (F2-IsoPs) and phospholipase A2 (PLA2) expression. Although total F2-IsoP levels were unchanged in PE placentas (*n* = 17) compared to normotensive controls (*n* = 15), free isomers like 8-iso-PGF2α and 15(R)-PGF2α were significantly elevated (> 40%, *p* ≤ 0.033). Concurrent upregulation of PLA2 isoforms (PLA2G2A, PLA2G5) and thromboxane receptor (TBXA2R) mRNA, alongside increased thromboxane B2 (TXB2; *p* = 0.005), suggested that PLA2-mediated release of free F2-IsoPs contributes to placental oxidative injury and hypertension in PE.

## Challenges, limitations, and considerations in antioxidant assays

Understanding the strengths, limitations, and interferences of antioxidant assays is crucial for deriving accurate and biologically meaningful conclusions (Apak, 2019). Despite their essential role in assessing the health-promoting potential of natural compounds, antioxidant assays face multiple challenges—chief among them are methodological limitations, reproducibility concerns, and poor physiological relevance (Carocho & Ferreira, 2013; Pasqualetti et al., 2021). These issues become particularly pronounced when comparing in vitro and in vivo models, which, while complementary, often yield conflicting results due to differences in biological complexity, compound bioavailability, and metabolic transformation.

In vitro assays such as DPPH, FRAP, and ORAC offer rapid, reproducible, and cost-effective methods for assessing radical scavenging or reducing power in a controlled environment. These assays are excellent for initial screening, as they isolate antioxidant mechanisms to simplified chemical reactions (Alam et al., 2013). However, this simplification is also their main limitation—they fail to replicate the dynamic physiological conditions in living organisms. For example, a compound may exhibit high antioxidant capacity in ORAC but be rendered ineffective in vivo due to poor absorption, rapid metabolism, or inability to reach the target site (Niki, 2010). These factors are not captured in in vitro systems and lead to overestimations of therapeutic potential.

In contrast, in vivo assays—such as measurements of lipid peroxidation (MDA), antioxidant enzymes (SOD, CAT, GSH), and systemic oxidative stress markers (e.g., isoprostanes)—provide a more realistic evaluation of a compound’s biological effects. These methods incorporate pharmacokinetic parameters, including absorption, distribution, metabolism, and excretion (ADME), as well as cellular signaling and interactions with endogenous biomolecules. However, in vivo models are not without limitations. Species-specific differences in metabolic pathways can hinder the extrapolation of animal data to humans (Saeidnia et al., 2015). Moreover, variability in genetic background, diet, and environmental exposures can introduce inconsistency, while ethical concerns and cost limit the scalability of these studies (Loh & Lim, 2018a, b; Burić et al., 2019; Torre et al., 2019).

The discrepancy between in vitro and in vivo findings is well illustrated in studies of plant-based antioxidants. For instance, *Dodonaea viscosa* extracts showed distinct behavior across models. In vitro, flower-containing seed extracts had higher GSH levels, while leaves excelled at H₂O₂ scavenging. However, both were sensitive to metal-induced oxidative stress, demonstrating the constraints of in vitro evaluations under biologically irrelevant conditions. In in vivo rat models, oral administration of the same extracts significantly increased GSH, GST, and CAT levels in liver and kidney tissues—although less potently than vitamin C-underscoring the added value of physiological validation (Khan et al., 2023).

Similarly, *Puerariae lobatae Radix* (PV) demonstrated strong in vitro antioxidant activity in DPPH and hydroxyl radical scavenging assays. Yet its systemic effects—including the restoration of body weight, reduction of MDA levels, and enhancement of endogenous antioxidant enzymes in a diarrhea-induced rat model—were only evident through in vivo assessment (Yang et al., 2025). Cellular assays confirmed PV’s ability to suppress ROS in macrophages, while network pharmacology and mRNA/protein validation identified key molecular targets (e.g., BCL2L1, ESR1, JAK2/STAT3), revealing a broader biological impact. Such integrated approaches—combining chemical assays, cellular models, and animal studies—are essential to bridge the gap between observed radical-scavenging capacity and true therapeutic potential.

Further emphasizing this point, Hawash et al. (2022) demonstrated that while in vitro assays revealed PV’s free radical-neutralizing effects, in vivo assessments captured its modulation of endogenous defenses and protective efficacy in oxidative-stress-induced intestinal damage. These complementary findings highlight that in vitro tests serve as a starting point for candidate identification, whereas in vivo validation ensures physiological relevance, particularly when evaluating antioxidant mechanisms across cellular, tissue, and systemic levels.

Adding to the complexity is the lack of standardized protocols and reference materials across laboratories, which significantly hampers reproducibility (Apak, 2019; Ilyasov et al., 2020). Slight differences in assay conditions—pH, solvent type, incubation time, or interfering compounds—can yield vastly different results for the same antioxidant compound (Gülçın, 2020). Furthermore, assays such as TEAC and ORAC often misrepresent antioxidant potential in biological matrices due to complex interactions that these chemical assays cannot replicate (Carocho & Ferreira, 2013; Pasqualetti et al., 2021). This inconsistency is amplified by the variability in assay sensitivity and specificity. For example, FRAP measures the ferric reducing ability of plasma, while TEAC evaluates the ability to neutralize ABTS radicals—two mechanisms that may not be directly comparable or physiologically relevant (Müller et al., 2011; Shi et al., 2010).

Ex vivo and cell-based models attempt to bridge in vitro and in vivo methodologies but introduce their own challenges. For example, sample preparation techniques like freezing and thawing may alter the integrity of tissue samples, affecting antioxidant readings (Steyn et al., 2025). Moreover, reliance on cancer cell lines, which often have altered metabolic profiles, can limit generalizability to normal physiological conditions (George et al., 2014; Arouna et al., 2020). These limitations highlight the importance of carefully selecting and validating experimental models based on the research question and desired biological relevance.

## Suggestions and future perspectives

The future of antioxidant assays in in vitro, ex vivo, and in vivo research is poised to revolutionize multiple fields including personalized medicine, nutraceuticals, cosmetics, and environmental monitoring. These assays provide critical insights into antioxidant mechanisms, safety profiles, and therapeutic efficacy against oxidative stress, which underlies aging, various diseases, and environmental damage. Future advancements will focus on enhancing accuracy, reliability, and real-world applicability through technological innovation, standardization, and interdisciplinary collaboration. For in vitro assays, which currently serve as the foundational screening tool despite limitations in physiological relevance (Apak, 2019; Sharpe et al., 2013), key priorities include the development of nanotechnology-based platforms employing gold nanoparticles and nanoceria to improve sensitivity and portability (Danet, 2021a, b), while addressing challenges such as nanoparticle cytotoxicity through optimized synthesis methods (Sharpe et al., 2013). Future directions also emphasize integrating multiple antioxidant mechanisms like hydrogen atom transfer and electron transfer (Apak, 2019), implementing high-throughput screening for natural product discovery (Santonocito et al., 2020), and adopting microfluidic technologies to reduce sample volumes while increasing precision (Rahman et al., 2018). Standardization of protocols remains crucial for reproducibility (Alam et al., 2013; Apak et al., 2016), along with incorporating omics technologies to elucidate antioxidant pathways (Kasote et al., 2015; Vitale et al., 2020).

Ex vivo assays, bridging the gap between in vitro and in vivo models, require future development of advanced cell culture systems such as 3D models and organ-on-a-chip platforms to better mimic human tissue complexity (Vitale et al., 2020). Priorities include the integration of real-time monitoring through biosensors and fluorescent probes (Ye et al., 2020), simulation of gastrointestinal processes to assess bioaccessibility (Lang et al., 2024), and investigation of synergistic combinations with vitamins and minerals (Zeng et al., 2020a, b). For in vivo studies, essential for clinical translation, future work must establish standardized measurements of TAC and biomarkers (Apak et al., 2016), while advancing toward personalized approaches that consider genetic and lifestyle factors (Yang et al., 2017). The field needs to validate findings through long-term clinical trials for chronic diseases (Power et al., 2012), explore novel antioxidant sources such as marine compounds (Alencar et al., 2019), and transition toward more physiologically relevant models including organoids (Boix et al., 2020).

The integration of emerging technologies represents a critical future direction across all assay types. Microfluidic systems combined with nanomaterials like quantum dots will enhance portability and sensitivity (Llorent-Martinez et al., 2017), while artificial intelligence can optimize experimental parameters and analyze complex datasets (Vitale et al., 2020). Automated systems such as flow injection analysis will improve efficiency (Pamunuwa & Atapattu, 2023), particularly when combined with microfluidics. Future research must prioritize establishing universal standardized protocols through collaborative efforts (Apak, 2019; Brainina et al., 2019), developing physiologically relevant assay conditions (Lang et al., 2024), and implementing comprehensive approaches that combine multiple assessment methods. The expansion of omics technologies will provide deeper mechanistic insights (Vitale et al., 2020), while real-time monitoring tools will enable dynamic assessment of antioxidant activity (Ye et al., 2020). Personalized approaches tailored to individual biomarkers (Yang et al., 2017) and rigorous clinical validation through long-term studies (Power et al., 2012) will be essential for translating findings into effective therapies. Exploration of novel antioxidant sources, including marine organisms and agricultural byproducts (Alencar et al., 2019), along with the continued development of portable detection systems for industrial and clinical applications, will further advance the field. By addressing these priorities, antioxidant research can bridge the gap between laboratory findings and practical applications, ultimately improving health outcomes and product efficacy across multiple sectors.

## Conclusion

The selection and application of antioxidant assays are critical for accurately evaluating antioxidant activity, as each method has distinct principles, advantages, and limitations. A multi-faceted approach, combining in vitro*, *ex vivo, and in vivo assays, is Erythrocyte membrane protein damage by phenoxyacetic herbicides and their metabolitesessential for a comprehensive understanding of antioxidant capacity, particularly in complex matrices like food or natural extracts. Future research should prioritize standardizing methodologies, integrating multiple assays, and exploring innovative approaches, such as the incorporation of nanoparticles and the application of artificial intelligence (AI). AI has the potential to revolutionize antioxidant research by enabling the analysis of large datasets, predicting antioxidant activity, optimizing experimental conditions, and identifying novel bioactive compounds. These advancements, alongside the integration of AI-driven tools, hold significant promise for advancing research in food science, pharmacology, and nutrition, while also contributing to sustainable and effective therapeutic strategies.

## Data Availability

No datasets were generated or analysed during the current study.
